# Toxic chinese herbal medicine recognition in real-world images via multi-scale and attention-enhanced EfficientNetV2

**DOI:** 10.1371/journal.pone.0344262

**Published:** 2026-03-19

**Authors:** Guohua Zhu, Jaehan Joo, Seonghyeon Park, Suk Chan Kim

**Affiliations:** Department of Electronic Engineering, Pusan National University, Busan, Republic of Korea; The University of Texas, MD Anderson Cancer Center, UNITED STATES OF AMERICA

## Abstract

Accurate identification of toxic Chinese herbal medicines is critical for public health and clinical safety. However, real-world herbal images often exhibit complex backgrounds and small, indistinct target regions, posing substantial challenges to automated classification systems. In this study, we present a novel image dataset comprising over 4,000 samples from 47 toxic herb categories, captured under diverse environmental conditions to reflect real-world variability. We benchmark several state-of-the-art convolutional neural networks, including ResNet, ResNeXt, and EfficientNet variants, and identify EfficientNetV2 as the most effective baseline. To further enhance model robustness and discriminative capability, we propose an improved EfficientNetV2 architecture incorporating two lightweight yet effective modules: a Multi-Scale Feature Fusion (MSFF) module to integrate hierarchical features, and a Convolutional Block Attention Module (CBAM) to refine both spatial and channel-wise representations. Experimental results demonstrate that our enhanced model achieves 91.28% Top-1 accuracy, 97.52% Top-5 accuracy, and a 90.27% macro F1-score, significantly outperforming baseline methods. Ablation studies confirm the complementary benefits of MSFF and CBAM, and targeted evaluations on challenging image subsets reveal improved resilience to background clutter and small object localization. The proposed architecture offers a high-accuracy, generalizable, and computationally efficient solution for toxic herbal medicine classification and provides a valuable reference for intelligent traditional medicine recognition applications. The GitHub repository for this project is available at: https://github.com/zhuguohua1992/Toxic-chinese-herbal-medicine-recognition-via-enhanced-EfficientNetV2.

## Introduction

Herbal medicine has played a vital role in traditional healthcare systems for centuries and continues to be widely employed in modern clinical practice for disease prevention, treatment, and rehabilitation [1–3]. Among the wide range of medicinal herbs, a considerable portion of which exhibit toxic properties that can pose significant health risks if misidentified or misused. Accidental ingestion of toxic herbs may result in allergic reactions, organ failure, or even fatal outcomes [4]. Consequently, accurate and efficient identification of toxic herbal species is critical for ensuring public safety and supporting safe clinical application.

Traditionally, toxic herb identification has relied on inspection by professionals trained in botany, pharmacognosy, and traditional materia medica. However, such expertise is often unavailable, particularly in high-throughput or resource-limited environments. Furthermore, the visual similarities between certain toxic herbs and their non-toxic counterparts increase the risk of misclassification, even among specialists [5]. While early efforts to automate this task relied on handcrafted features such as color moments, texture descriptors, and shape-based metrics [6–8], these methods lacked robustness and generalizability, particularly when applied to real-world images exhibiting lighting variation, background clutter, and morphological diversity.

With the advent of deep learning, Convolutional Neural Networks (CNNs) have emerged as the dominant approach for plant and herb classification tasks [9–11]. Several studies have demonstrated the effectiveness of CNNs in recognizing a wide range of medicinal plants [12,13], achieving state-of-the-art performance in both general object recognition and fine-grained species identification [14,15]. These technologies offer scalable and accurate alternatives to manual classification, enabling broader accessibility to automated herbal identification systems.

While most existing CNN-based approaches are trained on curated datasets collected under controlled conditions—featuring clean backgrounds, uniform lighting, and centrally positioned objects [16]—they often struggle to generalize in real-world applications. In practical scenarios, where herbal images may originate from mobile devices, web platforms, or field environments, these models encounter challenges such as inconsistent lighting, occlusion, background clutter, and small target regions. These complexities severely impair feature extraction and degrade overall classification performance.

The task of toxic herb identification introduces even greater difficulties, as it requires differentiating between numerous toxic species that exhibit highly similar visual characteristics in color, texture, and morphology. These subtle inter-class variations demand models with enhanced sensitivity and discriminative capacity. Moreover, the consequences of misclassification in this context can be medically serious, highlighting the urgent need for dedicated datasets and specialized models tailored specifically for toxic herb recognition. Despite the rapid progress of deep learning in general plant classification, focused research in this high-risk subdomain remains limited.

**Fig 1:**
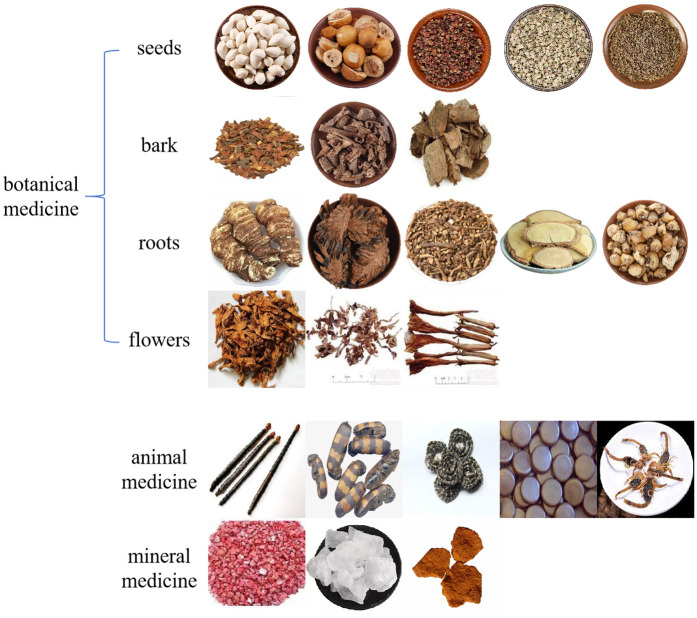
Sample images from the toxic herbal medicine dataset. The dataset was categorized based on the natural properties of the medicinal materials, which were grouped into botanical, animal, and mineral medicines. Botanical medicines included seeds (e.g., Purging Croton Seed, Ginkgo Seed), barks (e.g., Golden Larch Root, Chinese Silkvine Root), roots (e.g., Ternate Pinellia, Antifebrile Dichroa Root), and flowers (e.g., Hindu Datura Flower, Lilac Daphe Flower Bud). Animal medicines included examples such as Centipede and Dried Toad Venom, while mineral medicines included Red Orpiment and Cinnabar.

To address this gap, we curated a dedicated dataset comprising over 4,000 real-world images spanning 47 categories of toxic Chinese medicinal herbs. The images were captured under diverse environmental conditions to reflect variations in background complexity, illumination, and object scale. This dataset provides a realistic and challenging benchmark for evaluating model performance in unconstrained scenarios. Representative examples illustrating the dataset’s visual diversity are shown in Fig 1.

We conducted extensive comparative experiments evaluating several widely used CNN architectures, including ResNet [17], ResNeXt [18], EfficientNet [19], and EfficientNetV2 [20]. Among them, EfficientNetV2 achieved the best baseline performance; however, its accuracy remained suboptimal when confronted with background complexity and small target regions.

To mitigate these limitations, we propose an enhanced EfficientNetV2 model incorporating two lightweight yet effective modules: a Multi-Scale Feature Fusion (MSFF) module and a Convolutional Block Attention Module (CBAM). The MSFF module enables the network to aggregate semantic information across multiple receptive fields, thereby enhancing its ability to detect small and spatially diverse features [21]. The CBAM module refines feature representation through channel-wise and spatial attention mechanisms, helping the network focus on informative regions while suppressing irrelevant background noise [22].

We validated the proposed method through extensive experiments conducted on both the full dataset and a visually challenging subset. Ablation studies demonstrate that both MSFF and CBAM individually contribute to performance improvements, and their combination yields the highest classification accuracy. The resulting model exhibits strong robustness and generalization capability, offering a practical and effective solution for real-world toxic herb classification. Moreover, it establishes a promising foundation for the development of intelligent traditional medicine recognition systems applicable in real-world scenarios.

Recent advances in multiple instance learning (MIL) and vision foundation models have further expanded the landscape of medical and biological image analysis. MIL-based frameworks have demonstrated strong capability in handling complex visual patterns and weakly supervised settings in medical imaging and related domains [23,24]. More recently, large-scale vision foundation models have shown impressive generalization and cross-domain adaptability across diverse visual tasks [25]. Although these paradigms provide powerful alternatives, they typically require large computational resources or rely on coarse bag-level labeling, which differs from our instance-level toxic herb recognition scenario. In this work, we therefore focus on enhancing a lightweight and deployable CNN backbone while preserving strong discriminative performance in real-world herbal environments.

In summary, this study makes the following key contributions:

- We construct a novel data set of toxic medicinal herb images with diverse backgrounds and realistic imaging conditions.
- We evaluated multiple CNN architectures and identified EfficientNetV2 as a strong baseline.
- We propose two lightweight modules, MSFF and CBAM, to enhance feature extraction and attention in challenging visual conditions.
- We demonstrate, through extensive experiments and ablation studies, that our enhanced model significantly improves classification performance, especially on difficult samples.

## Materials and methods

We begin by describing the dataset construction, followed by the baseline architecture. Subsequently, we introduce two architectural enhancements: the MSFF module and the CBAM, which are integrated into the EfficientNetV2 backbone to enhance feature representation under challenging visual conditions. Finally, we outline the training strategy and implementation details used in our experiments.

## Dataset construction

### Image acquisition from online sources

To construct a diverse and representative dataset of toxic Chinese herbal medicines, we collected a large number of images from publicly accessible online sources, including herbal medicine databases, digital encyclopedias, educational platforms, and open-source image repositories. For each herb category, a range of search terms—including Latin binomials, Chinese pinyin, and commonly used vernacular names—was employed to retrieve a broad and inclusive set of candidate images.

To ensure compliance with ethical and legal requirements, only images explicitly designated for non-commercial or educational use were retained, and all data collection complied with the terms and conditions of the respective data sources. This web-based acquisition strategy enabled the compilation of a visually diverse dataset, capturing variations in camera angles, backgrounds, lighting conditions, and presentation formats.

### Data cleaning, annotation, and class labeling

The raw image set underwent a rigorous multi-stage cleaning process to ensure data quality and reliability. Each image was manually reviewed by annotators with expertise in pharmacognosy and traditional Chinese medicine to confirm botanical accuracy. Images that were blurry, mislabeled, overly repetitive, or irrelevant—such as those depicting packaging, unrelated plant species, or empty backgrounds—were removed.

To enhance dataset diversity and avoid redundancy, visually similar or nearly identical images were manually screened and eliminated. Each remaining image was labeled with one of 47 toxic herb categories. After this curation process, over 4,000 high-quality images were retained, with category sizes ranging from approximately 50–150 images. The final dataset was randomly split into training, validation, and test sets in a ratio of 3:1:1.

### Diversity and complexity of real-world imagery

In contrast to existing herbal datasets collected under idealized laboratory conditions, our dataset deliberately preserves a high degree of real-world variability to better simulate practical usage scenarios. The images exhibit substantial differences in visual composition, featuring diverse and often cluttered backgrounds such as natural environments, office spaces, and printed materials. Lighting conditions vary widely, ranging from well-lit outdoor scenes to dimly lit indoor settings with shadows or uneven exposure. The herbs are photographed from multiple viewpoints—including top-down, side, and oblique angles—often resulting in partial occlusions and perspective distortions. Furthermore, the dataset encompasses a wide array of physical forms, including dried slices, fresh roots, bundled specimens, and powdered substances.

Notably, in many images, the target herb occupies only a small portion of the frame and is surrounded by visually dominant or distracting elements. This variability introduces substantial intra-class diversity and lowers the signal-to-noise ratio, thereby increasing the difficulty of accurate classification. By retaining this complexity, the dataset provides a realistic and challenging benchmark for assessing the robustness and generalization ability of deep learning models in unconstrained environments.

### Construction of the small-target challenge subset

**Fig 2:**
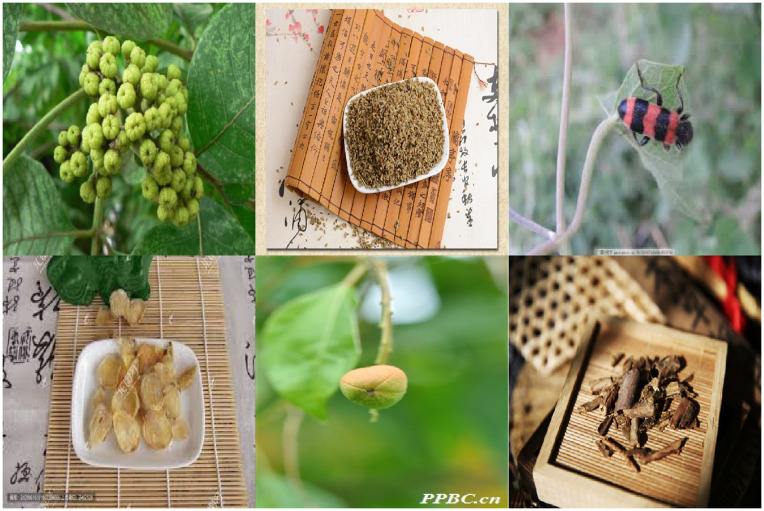
Sample images from a test subset of toxic herbal medicine data. The images exhibit diverse backgrounds, lighting conditions, and small object regions, posing challenges for robust classification and detection.

To evaluate model performance under visually challenging conditions, we curated a dedicated Small-Target and Complex-Background Challenge Set from the test dataset. This subset comprises 462 images in which the target herb appears either as a small object or within a cluttered and complex background, often accompanied by occlusion and varying lighting conditions. These characteristics closely resemble real-world usage scenarios where accurate herb identification is particularly difficult. Representative examples are shown in Fig 2.

Selection criteria for this subset were based on manual inspection and visual saliency estimates generated by preliminary baseline models. The subset contains numerous edge-case examples that resemble those commonly found in user-generated content or field-acquired data. It was used exclusively during the evaluation phase to assess the robustness and localization sensitivity of the proposed model enhancements, namely the MSFF and CBAM.

### Ethics statement

This study did not involve clinical trials, patient records, biological specimens, or the collection of personal or identifiable data. However, a group of human annotators participated in the manual review and labeling of images during dataset construction. All annotators were adults with expertise in pharmacognosy or traditional Chinese medicine and voluntarily participated in the annotation activity.

Written informed consent was obtained from all annotators prior to their participation, and consent forms are securely stored by the corresponding author. No minors were involved in this study. Since no identifiable or sensitive personal information was collected and the annotators’ role was limited to technical review and labeling, formal institutional ethics approval was not required.

## Architecture

### Baseline architecture

As a starting point, we adopted the EfficientNetV2 architecture as our baseline classifier due to its favorable balance between accuracy and computational efficiency. EfficientNetV2 combines MBConv and fused-MBConv blocks to accelerate training while maintaining rich representational capacity [20]. In addition, its compound scaling strategy uniformly adjusts network depth, width, and resolution, yielding consistent performance gains across different model sizes.

While EfficientNetV2 has achieved state-of-the-art results in general image classification tasks, it is not specifically tailored to handle domain-specific challenges commonly encountered in herbal image classification—such as background clutter, small target objects, and subtle inter-class visual similarities. To overcome these limitations, we introduce two architectural modules designed to enhance the model’s ability to extract fine-grained and localized features.

### Multi-scale feature fusion

The MSFF module is introduced to enhance the model’s ability to capture features across multiple semantic levels. In deep convolutional networks, shallow layers typically retain fine-grained spatial details, whereas deeper layers encode more abstract semantic information. By fusing these complementary features, the model becomes more robust in scenarios involving small, partially occluded, or visually ambiguous target regions.

**Fig 3:**
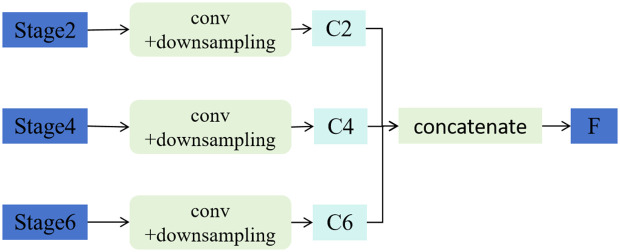
Illustration of the MSFF module. Features from different stages (Stage2, Stage4, and Stage6) are first processed by convolution and downsampling operations to generate consistent channel representations (C2, C4, C6), which are then concatenated to form the fused feature map *F*.

In our implementation, three intermediate feature maps were extracted from the EfficientNetV2 backbone. Each map was first processed with a 1×1 convolution to unify channel dimensions. Spatial alignment was then achieved by either bilinear upsampling or strided downsampling, depending on the resolution of the input feature map. The aligned features were concatenated along the channel axis and subsequently passed through a 3×3 convolution, followed by batch normalization and ReLU activation, to generate the fused multi-scale representation. This fused output encodes both local detail and global context, which is essential for accurately recognizing small and morphologically subtle herbal structures. An overview of the MSFF architecture is illustrated in Figure 3.

### Convolutional block attention module

To further enhance the model’s discriminative capacity, we integrated the CBAM after the MSFF output. CBAM applies attention in two sequential stages: channel attention and spatial attention. In the channel attention stage, global average pooling and max pooling are independently applied across the spatial dimensions, and the resulting descriptors are passed through shared multi-layer perceptrons followed by a sigmoid activation to produce a channel-wise attention map. This map is then multiplied element-wise with the input feature map, allowing the model to emphasize informative feature channels.

The spatial attention stage follows by refining the output of the channel attention. It compresses the feature map along the channel axis using both average and max pooling, then applies a 7×7 convolution followed by a sigmoid activation to generate a spatial attention map. This spatial mask highlights relevant regions and suppresses background noise. The final CBAM output is obtained by sequentially applying both attention maps to the input features via element-wise multiplication.

**Fig 4:**
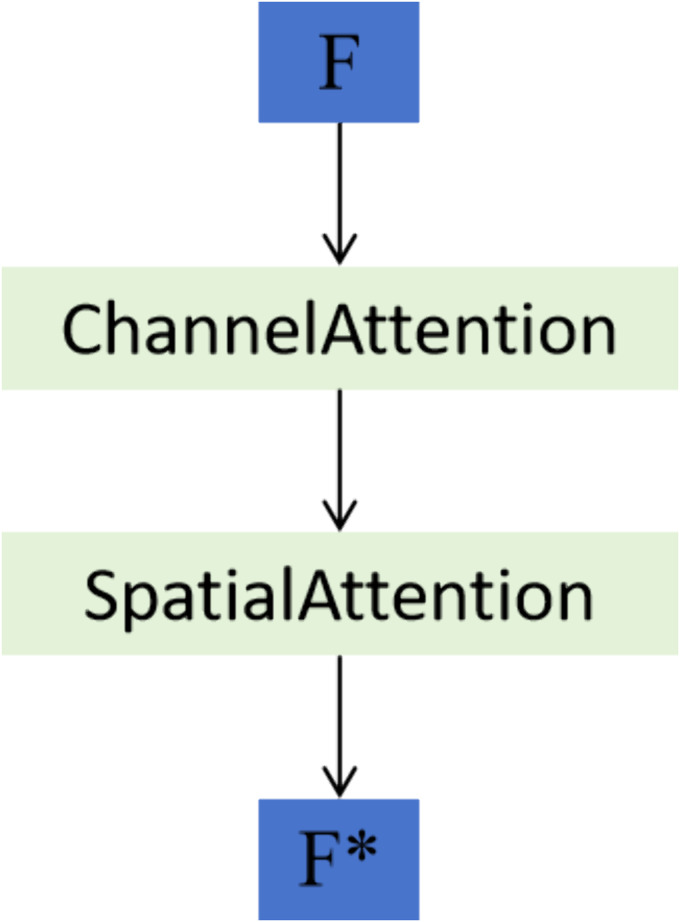
Structure of the CBAM. The fused feature map *F* is first refined by the Channel Attention mechanism, followed by the Spatial Attention mechanism, resulting in the enhanced feature representation $F^{*}\;$.

By placing CBAM after MSFF, the network is guided to focus more precisely on salient herbal features, even in the presence of complex or cluttered backgrounds. An overview of the CBAM integration is illustrated in Figure 4.

### Integrated network architecture

The overall architecture comprises three key components: the EfficientNetV2 backbone, the MSFF module, and the CBAM. Feature maps from selected intermediate layers of the backbone are first aggregated by the MSFF module to produce a unified multi-scale representation. This representation is subsequently refined by the CBAM, which directs attention toward salient herbal regions while suppressing irrelevant background noise.

**Fig 5:**
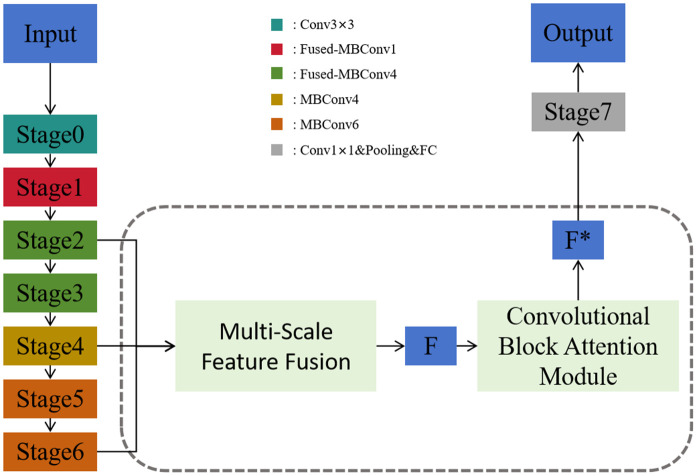
The overall architecture of the improved EfficientNetV2-based classification model. Features from multiple stages (Stage0 to Stage6) are fused via a MSFF module, followed by a CBAM to enhance salient regions. The final refined feature map $F^{*}\;$ is fed into the classifier for prediction. This design aims to improve recognition performance under complex backgrounds and varying object scales.

The attention-enhanced features are then passed through a global average pooling layer followed by a fully connected classification head to produce the final class logits. This design maintains the lightweight and efficient nature of EfficientNetV2 while significantly boosting robustness and accuracy under visually challenging conditions. An overview of the complete architecture is illustrated in Fig 5.

### Training strategy

The network was trained to minimize the cross-entropy loss across 47 toxic herb categories. We employed the Stochastic Gradient Descent (SGD) optimizer with a momentum of 0.9 to ensure stable convergence. The initial learning rate was set to 0.001 and was gradually reduced using a cosine annealing schedule throughout the training process. Each model was trained for 100 epochs with a batch size of 16. All input images were resized to 224×224 pixels to comply with the input size requirements of the EfficientNetV2 backbone.

To mitigate overfitting and enhance generalization, we applied a range of data augmentation techniques, including random cropping, horizontal flipping, and color jittering. All experiments were implemented using the PyTorch framework and executed on NVIDIA GPUs.

## Results

In this section, we present a comprehensive series of experiments to evaluate the effectiveness of the proposed toxic Chinese herbal medicine classification model. We begin by introducing the evaluation metrics and experimental setup. Next, we compare the performance of our enhanced EfficientNetV2 architecture against several baseline CNN models. We then conduct ablation studies to examine the individual and combined contributions of the MSFF and CBAM modules. Finally, we carry out targeted evaluations on visually challenging samples to assess the model’s robustness and generalization ability under realistic conditions.

### Evaluation metrics and experimental setup

To comprehensively assess classification performance, we employ five evaluation metrics: Top-1 Accuracy, Top-5 Accuracy, Mean Precision, Mean Recall, and Macro F1 Score. Top-1 Accuracy quantifies the percentage of samples for which the top predicted label matches the ground truth. Top-5 Accuracy considers a prediction correct if the ground truth label appears within the top five predicted classes. Mean Precision and Mean Recall represent the unweighted average of per-class precision and recall, providing insight into the consistency of predictions across all categories. The Macro F1 Score combines these measures into a single indicator, offering a robust assessment of model performance, especially in imbalanced multi-class scenarios.

All models were trained and evaluated using the dataset split described in Section 2.1.2. The best-performing checkpoint on the validation set was selected for final testing. All experiments were implemented in PyTorch and executed on NVIDIA RTX GPUs. To ensure a fair comparison, an identical training strategy and hyperparameter configuration were applied across all evaluated models.

### Baseline model comparison

We first compared the classification performance of our enhanced EfficientNetV2 model against several widely-used CNN architectures, including ResNet-18, ResNet-50, ResNet-152, ResNeXt-50, ResNeXt-101, ResNeXt-152, EfficientNet, and the vanilla EfficientNetV2 model. All models were trained from scratch on our dataset using identical preprocessing and augmentation pipelines.

Table 1 presents a comparative evaluation of several CNN-based models on the toxic Chinese herbal image dataset. The results indicate that deeper models generally achieve higher accuracy and robustness. Among all evaluated architectures, EfficientNetV2 achieved the best overall performance, with a Top-1 Accuracy of 89.19%, Mean Precision of 88.89%, Mean Recall of 88.17%, and a Macro F1 Score of 88.21%, demonstrating its strong capability in classifying toxic herbal images under complex visual conditions.

**Table 1:** Performance comparison of various CNN architectures on toxic herbal image classification. EfficientNetV2 achieves the highest performance across all metrics.

Method	Top-1 Acc (%)	Top-5 Acc (%)	Mean Precision (%)	Mean Recall (%)	Mean F1 Score (%)
ResNet18	79.04	96.22	77.90	77.08	77.04
ResNet34	79.56	95.05	79.20	77.53	77.73
ResNet50	82.68	95.70	82.06	81.24	81.23
ResNet101	83.46	97.01	83.98	81.75	82.21
ResNet152	85.55	97.53	84.77	84.38	84.11
ResNeXt50	87.11	97.27	86.83	85.49	85.74
ResNeXt101	87.89	97.40	87.74	86.39	86.54
ResNeXt152	86.84	97.27	86.84	85.84	85.80
EfficientNet	87.37	97.40	87.39	86.13	86.36
EfficientNetV2	**89.19**	**97.43**	**88.89**	**88.17**	**88.21**
Vision Transformer	36.85	66.93	39.47	35.39	35.28
Swin Transformer	30.99	64.45	29.93	28.59	27.34

To provide a more comprehensive and fair baseline comparison and to assess the potential of modern transformer architectures for toxic herbal image classification, we additionally evaluated two transformer-based models, including Vision Transformer (ViT) [26] and Swin Transformer [27], trained from scratch under the same settings. As shown in Table 1, both models exhibited significantly lower performance compared with CNN-based architectures. In particular, ViT and Swin Transformer achieved Top-1 accuracies of only 36.85% and 30.99%, respectively, confirming that transformer models without large-scale pretraining struggle to learn robust representations for fine-grained herbal classification.

**Fig 6:**
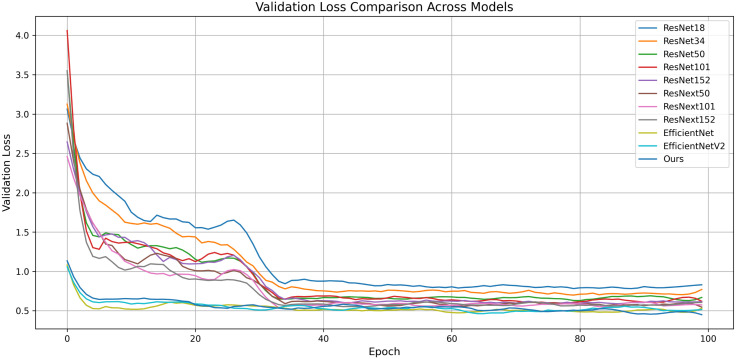
Validation Loss Comparison Across Models. This figure compares the validation loss curves of our proposed model with several state-of-the-art convolutional neural networks.

Figure 6 illustrates the validation loss curves of several backbone models, including the ResNet family (ResNet-18, ResNet-34, ResNet-50, ResNet-101, ResNet-152), ResNeXt variants (ResNeXt-50, ResNeXt-101, ResNeXt-152), EfficientNet, EfficientNetV2, and our proposed model (“Ours”). The x-axis represents the number of training epochs, while the y-axis denotes the corresponding validation loss. All models exhibit a steep decline in validation loss during the initial training phases, followed by gradual convergence.

### Ablation study

To quantify the individual contributions of the MSFF and CBAM modules, we performed an ablation study by incrementally incorporating each component into the baseline EfficientNetV2 architecture.

Table 2 summarizes the results. While the inclusion of either MSFF or CBAM alone leads to noticeable performance improvements, CBAM demonstrates a slightly greater impact. The combination of both modules yields the highest Top-1 Accuracy and Macro F1 Score, highlighting their complementary strengths in enhancing toxic herbal image classification.

**Table 2:** Ablation study of MSFF and CBAM modules on EfficientNetV2. The combination of both modules achieves the best performance across all evaluation metrics.

Method	MSFF	CBAM	Top-1 Acc (%)	Top-5 Acc (%)	Mean Precision (%)	Mean Recall (%)	Mean F1 Score (%)
EfficientNetV2 (Baseline)	✗	✗	89.19	97.43	88.89	88.17	88.21
EfficientNetV2 + MSFF	✓	✗	88.67	97.12	89.44	87.28	87.82
EfficientNetV2 + CBAM	✗	✓	90.10	97.38	90.57	88.72	89.18
EfficientNetV2 + MSFF + CBAM	✓	✓	**91.28**	**97.52**	**91.20**	**90.17**	**90.27**

In addition to classification accuracy, the joint integration of MSFF and CBAM further improves Mean Precision and Mean Recall, indicating enhanced model capability in both identifying relevant features and capturing class completeness under real-world variability. These findings validate the effectiveness of multi-scale feature aggregation and attention refinement in tackling the challenges posed by small targets and complex backgrounds.

Notably, the proposed model consistently maintains lower validation loss across all epochs compared to the baseline architectures, indicating superior generalization and robustness on unseen data. These results validate the effectiveness of the architectural enhancements introduced in our network for toxic Chinese herbal medicine classification.

Based on these findings, EfficientNetV2 was selected as the backbone architecture for integrating the proposed MSFF and CBAM modules.

### Performance on visually challenging samples

To further assess the model’s robustness under real-world conditions, we curated a subset of approximately 462 test images featuring small target occupancy and complex backgrounds (e.g., cluttered scenes, embedded text, or human-related elements). This subset captures visual challenges that are often absent in standard benchmarks.

Table 3 presents the ablation results on this challenging subset. Both MSFF and CBAM independently contribute to improved performance, while their combined integration leads to substantial gains across all evaluation metrics. These results underscore the effectiveness of multi-scale feature fusion and attention mechanisms in addressing difficult classification scenarios. Notably, the observed improvements in Mean Precision and Mean Recall indicate that the proposed modules effectively mitigate performance degradation caused by background noise and partial object occlusion.

**Table 3:** Ablation study on a challenging subset with complex backgrounds and small herbal targets. MSFF and CBAM modules improve classification performance significantly, especially when combined.

Method	MSFF	CBAM	Top-1 Acc (%)	Top-5 Acc (%)	Mean Precision (%)	Mean Recall (%)	Mean F1 Score (%)
EfficientNetV2 (Baseline)	✗	✗	89.20	97.88	89.29	87.87	88.13
EfficientNetV2 + MSFF	✓	✗	90.62	97.88	90.38	88.76	89.21
EfficientNetV2 + CBAM	✗	✓	90.44	97.70	90.07	88.92	89.05
EfficientNetV2 + MSFF + CBAM	✓	✓	**92.92**	**98.21**	**93.39**	**91.58**	**91.93**

Our proposed model exhibits substantial improvements over the baseline in this challenging scenario, underscoring its enhanced capability to detect and classify small, visually occluded herbal targets in the presence of background clutter.

## Discussion

### Feature visualization and interpretability analysis

To gain deeper insights into the internal mechanisms of our enhanced network, we performed a feature visualization analysis by comparing intermediate activation maps before and after the integration of the MSFF and CBAM modules. This intra-model comparison provides concrete evidence of how these architectural enhancements improve spatial focus and semantic representation, thereby contributing to superior classification performance under visually complex conditions.

**Fig 7:**
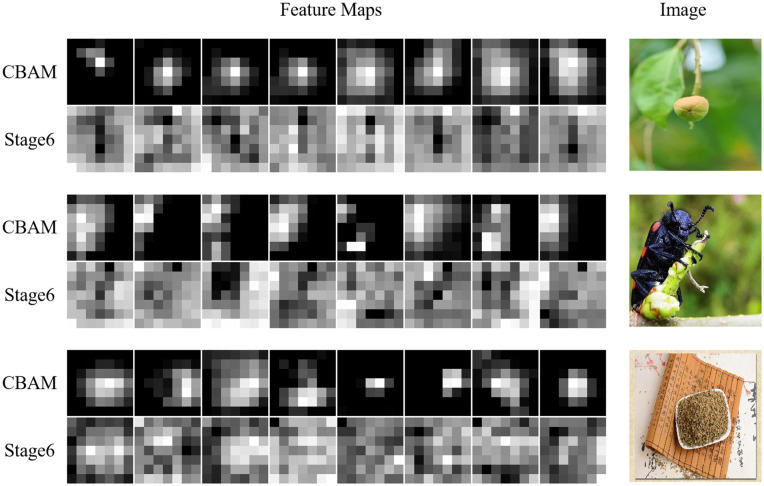
Visualization of feature maps before and after the MSFF and CBAM modules. Top row: feature maps after MSFF and CBAM show focused, high-response activations aligned with the herbal target regions, even in complex backgrounds. Bottom row: feature maps extracted prior to the enhancement modules exhibit diffuse attention with weak localization.

As shown in Figure 7, the feature maps extracted from the final backbone layer—prior to MSFF integration—exhibit diffuse and ambiguous activation patterns. Although these maps contain relevant semantic information, the attention is often dispersed across both target and non-target regions, particularly in cases where the herbal object occupies a small portion of the image or is surrounded by substantial background noise. Such distributed focus can reduce the model’s ability to accurately localize and classify the herbal target.

In contrast, after applying the MSFF and CBAM modules, the resulting activation maps demonstrate significantly improved spatial concentration around the relevant object regions. The MSFF module enriches the representation by fusing multi-scale features from different backbone depths, effectively combining fine-grained details with high-level semantic context. Subsequently, the CBAM module refines the output further by attenuating irrelevant background responses and selectively enhancing the most discriminative channels and spatial locations. Together, these modules guide the network to focus more precisely on the target herb, even under challenging visual conditions.

This focused attention is especially pronounced in scenes characterized by small targets or visual clutter, where the post-CBAM activation maps exhibit high-intensity responses precisely over the herbal regions, while irrelevant background areas are substantially suppressed. The transition from diffuse to sharply localized activations highlights the effectiveness of the proposed modules in steering the network’s focus toward task-relevant features, thereby improving both robustness and interpretability.

These qualitative observations are consistent with our quantitative results, where the integration of MSFF and CBAM yielded notable improvements in Top-1 accuracy and Macro F1-score. Importantly, they reveal that the observed performance gains are not merely numerical artifacts but are grounded in meaningful alterations to the model’s internal representations—demonstrating an enhanced ability to perceive, discriminate, and process visual information in complex scenarios.

### Comparison with transformer-based baselines

In addition to the qualitative evidence above, we further compared our enhanced CNN-based architecture with two transformer-based baselines, Vision Transformer (ViT) and Swin Transformer, trained from scratch under identical settings. Both models demonstrated substantially lower performance compared with convolutional architectures, achieving Top-1 accuracies of only 36.85% and 30.99%, respectively, far below the 89.19% obtained by our enhanced EfficientNetV2 model.

This performance gap is aligned with findings in recent literature that transformer-only models typically require large-scale pretraining to establish robust visual representation capabilities. Unlike CNNs, which inherently incorporate inductive biases such as spatial locality and translation equivariance, transformers rely heavily on data-driven learning and therefore struggle when trained from scratch on relatively small datasets. Furthermore, the patch embedding strategy employed by ViT and the window-based self-attention used in Swin Transformer limit their ability to effectively capture small herbal structures and long-range contextual dependencies in cluttered real-world scenes. Consequently, attention regions become unstable and diffuse, leading to substantial underfitting and degraded classification performance.

These observations collectively indicate that while transformer architectures possess strong potential in large-scale or pretrained regimes, CNN-based backbones remain more suitable for fine-grained toxic herbal image recognition under limited data conditions. Future work will explore fine-tuning large pretrained transformer models and multimodal fusion strategies to evaluate whether pretrained knowledge can overcome current limitations.

### Relation to recent MIL and foundation-model approaches

Recent literature has explored more advanced learning paradigms, including multiple instance learning (MIL) for complex medical image interpretation [23,24] and large-scale vision foundation models with strong cross-domain generalization capabilities [25]. MIL-based systems are particularly effective when only coarse bag-level annotations are available or when the target must be inferred by aggregating multiple image regions, which differs from our setting where each herbal image carries explicit instance-level labels. Vision foundation models, while powerful, typically require extensive computational resources and large-scale multimodal datasets, making them less suitable for lightweight deployment in clinical or pharmacy environments.

Nevertheless, these directions provide promising avenues for future research. Incorporating MIL strategies may benefit scenarios involving multi-view herb specimens or uncertain labels, while foundation-model adaptation (e.g., parameter-efficient fine-tuning) could further enhance generalization once larger real-scene herbal datasets become available. We view our MSFF- and CBAM-enhanced EfficientNetV2 as a practical intermediate solution that balances accuracy, efficiency, and deployability for toxic herb identification in real-world settings.

## Conclusion

In this study, we proposed a comprehensive framework for the classification of toxic Chinese herbal medicines in real-world conditions. To address the lack of publicly available and realistically complex datasets, we constructed a novel image dataset comprising over 4,000 samples across 47 toxic herb categories. The dataset features diverse backgrounds, lighting conditions, and object scales, offering a valuable benchmark for evaluating the robustness of deep learning models in practical scenarios.

We conducted a systematic comparison of several state-of-the-art CNN architectures and identified EfficientNetV2 as the most suitable baseline. To enhance its capacity for handling small objects and background complexity, we introduced two lightweight yet effective architectural modules, the MSFF module and CBAM. When integrated into the EfficientNetV2 backbone, these modules significantly improved classification accuracy and generalization capability.

Extensive experiments demonstrated that the enhanced model consistently outperformed existing baselines across multiple evaluation metrics. Notably, the model achieved the greatest performance gains on a curated subset of visually challenging samples, confirming its robustness in complex environments. By balancing high accuracy, computational efficiency, and real-world applicability, the proposed approach offers a promising solution for the intelligent identification of toxic herbal medicines. Furthermore, consistent improvements in both Mean Precision and Mean Recall across standard and difficult datasets underscore the model’s effectiveness in maintaining high-quality predictions across all categories.

Future work will explore deployment of the model on mobile devices, integration of multimodal features such as chemical structures or textual metadata, and expansion of the dataset to include additional herb species and clinically sourced samples. In addition, we plan to investigate multiple instance learning frameworks and lightweight adaptations of vision foundation models [23–25], which may further enhance generalization to more diverse clinical and laboratory scenarios.

## Supporting information

S1 Fig Confusion matrix. Confusion matrix illustrating per-class classification accuracy across all 47 toxic herbal categories, with diagonal intensities indicating correct predictions and scattered off-diagonal values highlighting misclassification patterns among visually similar herbs.
(PNG)

S2 Fig Coverage accuracy curve. Coverage–accuracy trade-off curve illustrating performance improvement via confidence thresholding.
(PNG)

S3 Fig Background hue hist. Histogram of background hue values showing diverse real-world color distributions.
(PNG)

S4 Fig Illumination hist. Histogram of image brightness showing a wide illumination range across real-world samples.
(PNG)

S5 Fig Quality scatter. Scatter plot showing wide variation in image quality, with diverse contrast and sharpness levels across real-world samples.
(PNG)

S6 File Results and training logs. Numerical data underlying all figures and reported metrics, including complete training logs, evaluation results, per-class performance values, and confusion matrices.
(ZIP)

S1 Table Per class metrics. Confusion matrix of the 47-class toxic herbal medicine classification results.
(CSV)

S2 Table Coverage accuracy. Coverage–accuracy trade-off curve with confidence thresholding.
(CSV)
