# Supplementary figures and images for: Toxic chinese herbal medicine recognition in real-world images via multi-scale and attention-enhanced EfficientNetV2 (part 1 of 2)
Source: PLoS One. 2026 Mar 19;21(3):e0344262. doi: 10.1371/journal.pone.0344262 (PMC13002182; doi:10.1371/journal.pone.0344262)

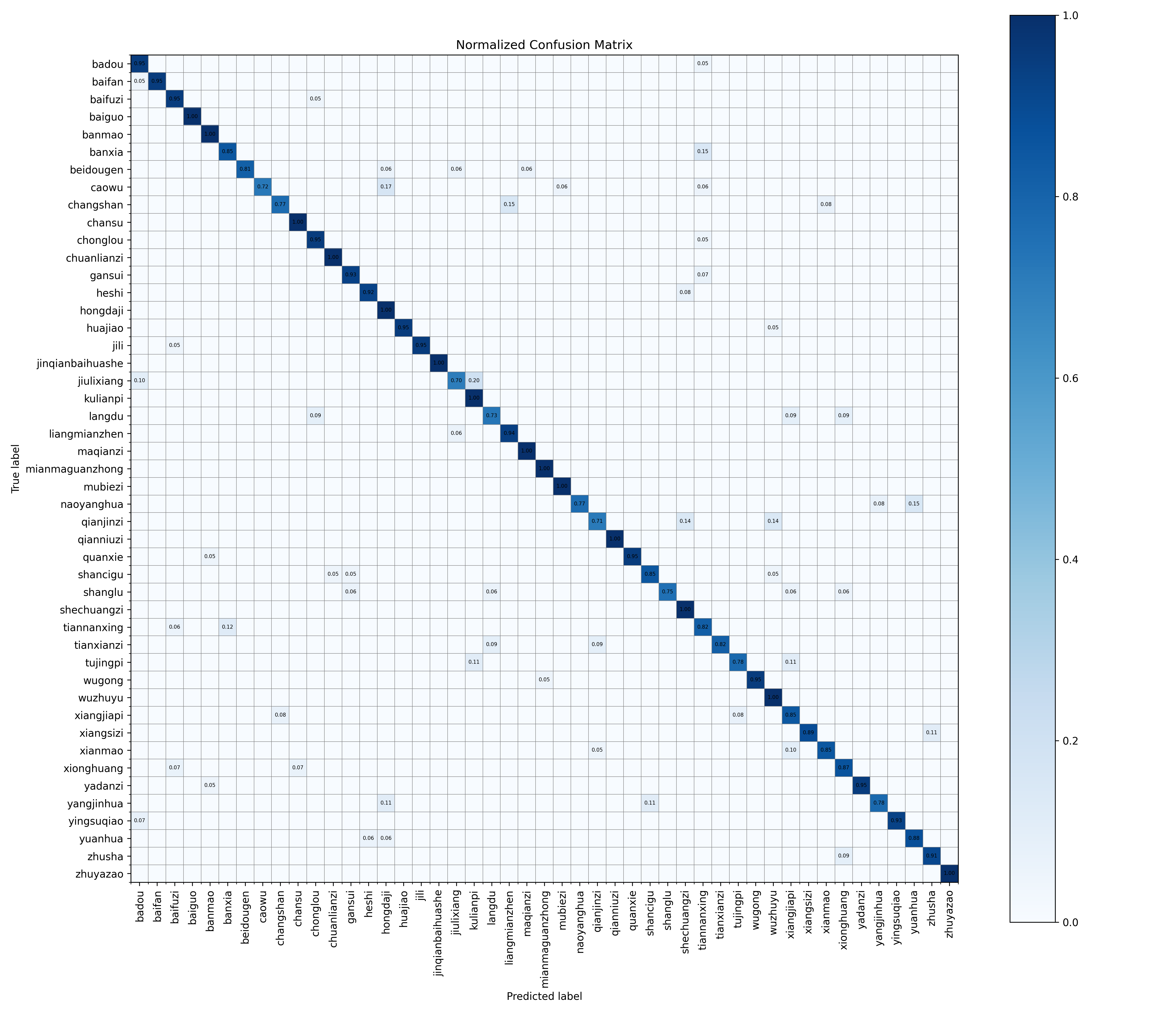

Supplement: S1 Fig — Confusion matrix illustrating per-class classification accuracy across all 47 toxic herbal categories, with diagonal intensities indicating correct predictions and scattered off-diagonal values highlighting misclassification patterns among visually similar herbs. (PNG) [file pone.0344262.s001.png]

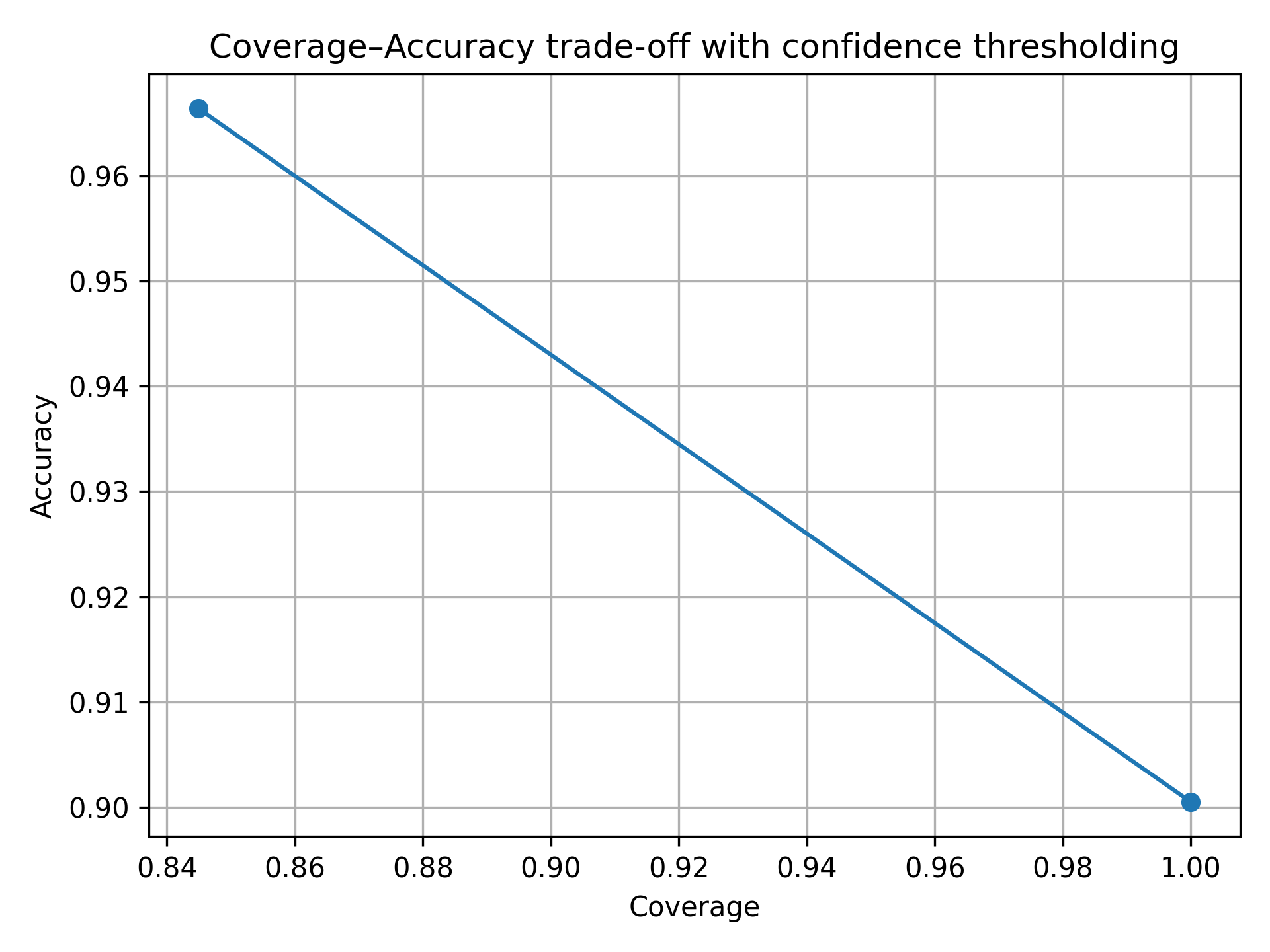

Supplement: S2 Fig — Coverage–accuracy trade-off curve illustrating performance improvement via confidence thresholding. (PNG) [file pone.0344262.s002.png]

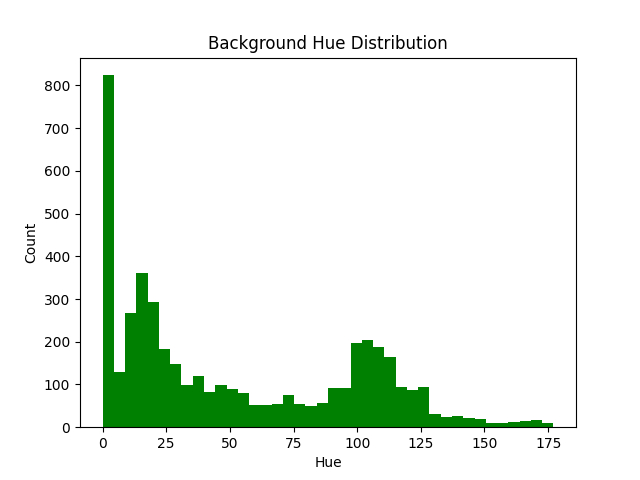

Supplement: S3 Fig — Histogram of background hue values showing diverse real-world color distributions. (PNG) [file pone.0344262.s003.png]

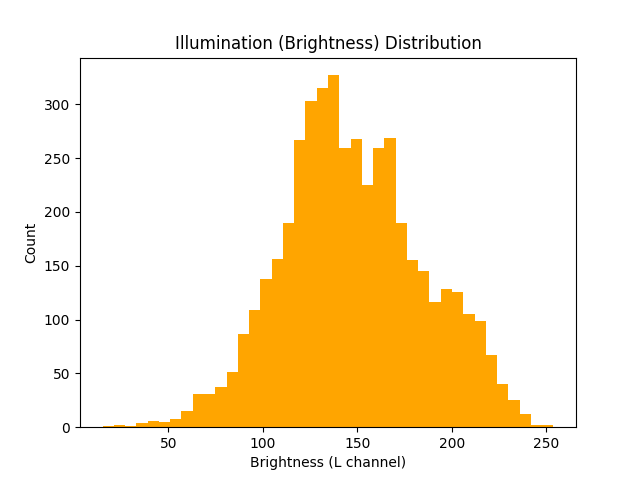

Supplement: S4 Fig — Histogram of image brightness showing a wide illumination range across real-world samples. (PNG) [file pone.0344262.s004.png]

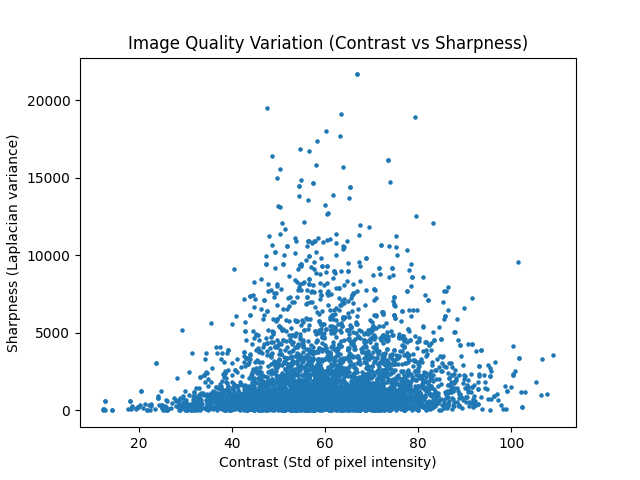

Supplement: S5 Fig — Scatter plot showing wide variation in image quality, with diverse contrast and sharpness levels across real-world samples. (PNG) [file pone.0344262.s005.png]

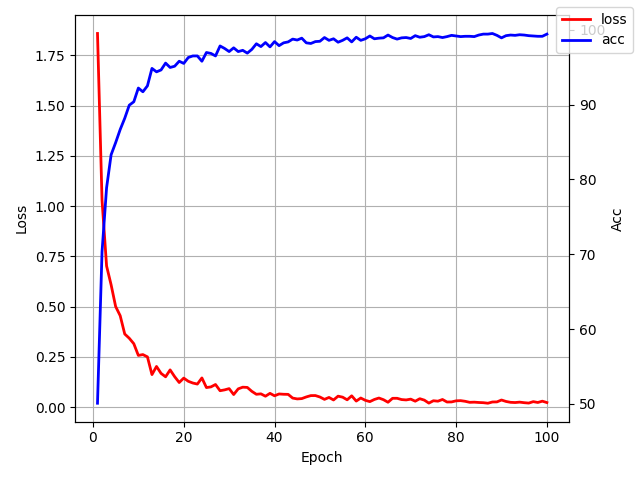

Supplement: S6 File — Numerical data underlying all figures and reported metrics, including complete training logs, evaluation results, per-class performance values, and confusion matrices. (ZIP) [file pone.0344262.s006.zip › Dataset/results/EfficientNetV2/train_loss-acc.png]

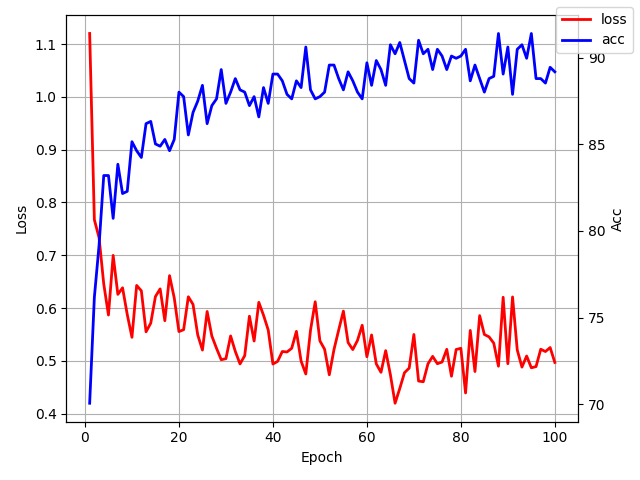

Supplement: S6 File — Numerical data underlying all figures and reported metrics, including complete training logs, evaluation results, per-class performance values, and confusion matrices. (ZIP) [file pone.0344262.s006.zip › Dataset/results/EfficientNetV2/val_loss-acc.png]

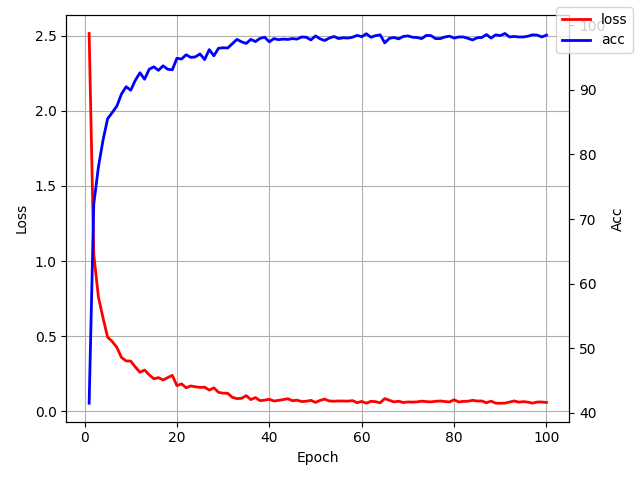

Supplement: S6 File — Numerical data underlying all figures and reported metrics, including complete training logs, evaluation results, per-class performance values, and confusion matrices. (ZIP) [file pone.0344262.s006.zip › Dataset/results/EfficientNet/train_loss-acc.png]

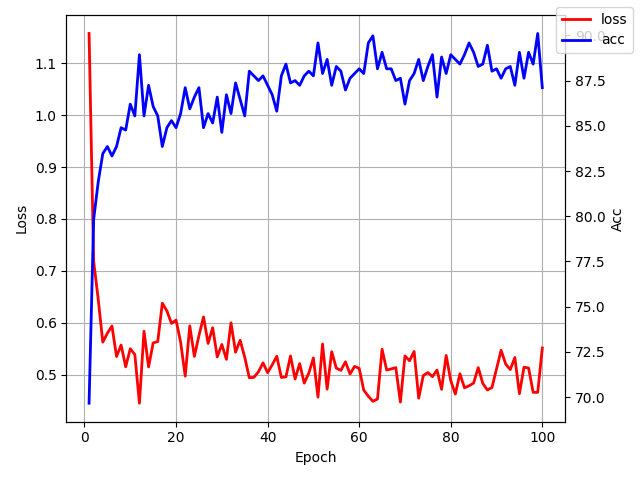

Supplement: S6 File — Numerical data underlying all figures and reported metrics, including complete training logs, evaluation results, per-class performance values, and confusion matrices. (ZIP) [file pone.0344262.s006.zip › Dataset/results/EfficientNet/val_loss-acc.png]

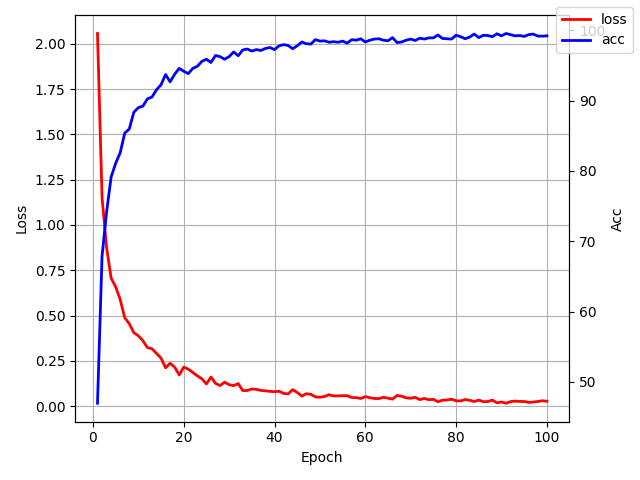

Supplement: S6 File — Numerical data underlying all figures and reported metrics, including complete training logs, evaluation results, per-class performance values, and confusion matrices. (ZIP) [file pone.0344262.s006.zip › Dataset/results/Ours/train_loss-acc.png]

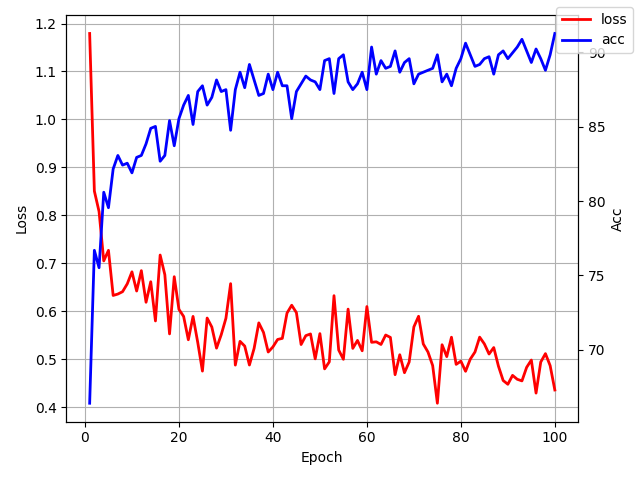

Supplement: S6 File — Numerical data underlying all figures and reported metrics, including complete training logs, evaluation results, per-class performance values, and confusion matrices. (ZIP) [file pone.0344262.s006.zip › Dataset/results/Ours/val_loss-acc.png]

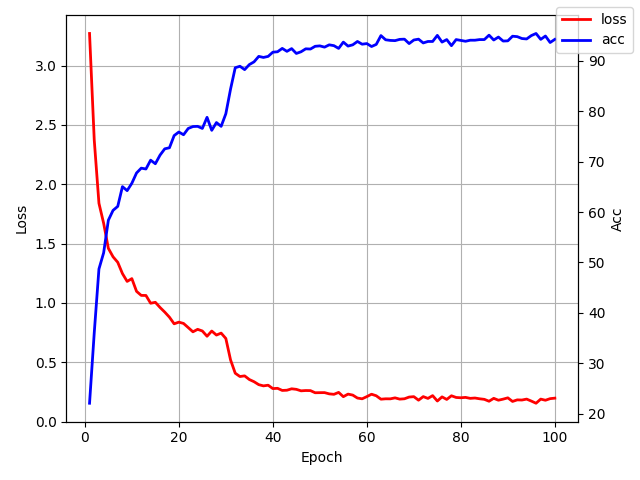

Supplement: S6 File — Numerical data underlying all figures and reported metrics, including complete training logs, evaluation results, per-class performance values, and confusion matrices. (ZIP) [file pone.0344262.s006.zip › Dataset/results/ResNet101/train_loss-acc.png]

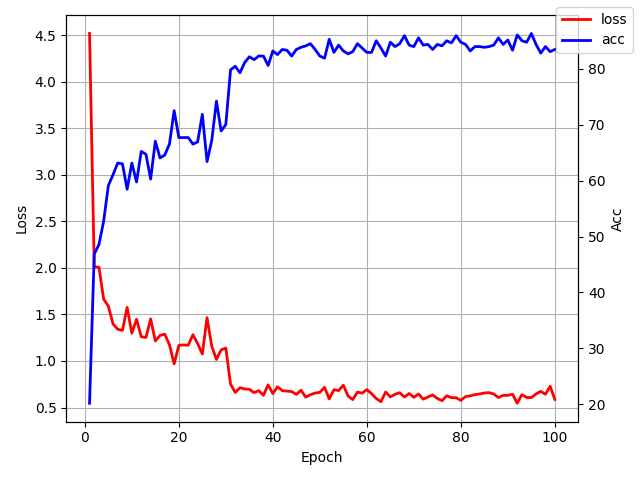

Supplement: S6 File — Numerical data underlying all figures and reported metrics, including complete training logs, evaluation results, per-class performance values, and confusion matrices. (ZIP) [file pone.0344262.s006.zip › Dataset/results/ResNet101/val_loss-acc.png]

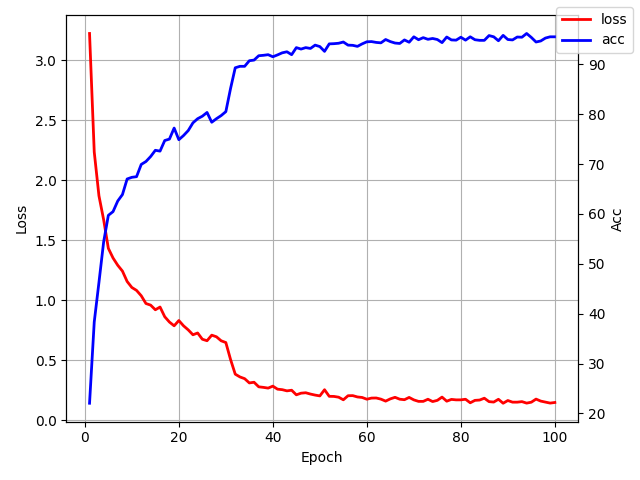

Supplement: S6 File — Numerical data underlying all figures and reported metrics, including complete training logs, evaluation results, per-class performance values, and confusion matrices. (ZIP) [file pone.0344262.s006.zip › Dataset/results/ResNet152/train_loss-acc.png]

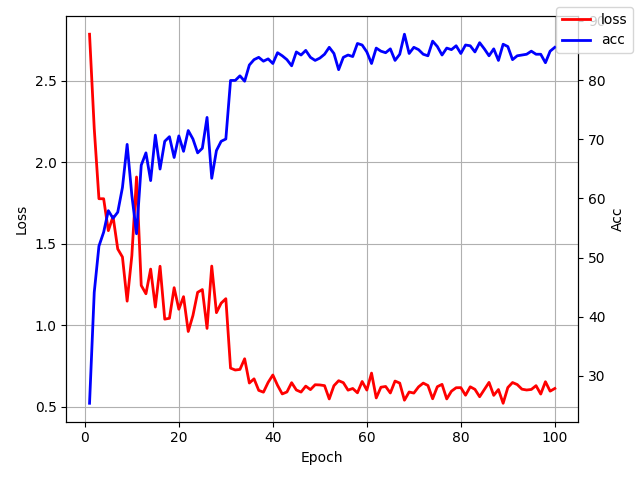

Supplement: S6 File — Numerical data underlying all figures and reported metrics, including complete training logs, evaluation results, per-class performance values, and confusion matrices. (ZIP) [file pone.0344262.s006.zip › Dataset/results/ResNet152/val_loss-acc.png]

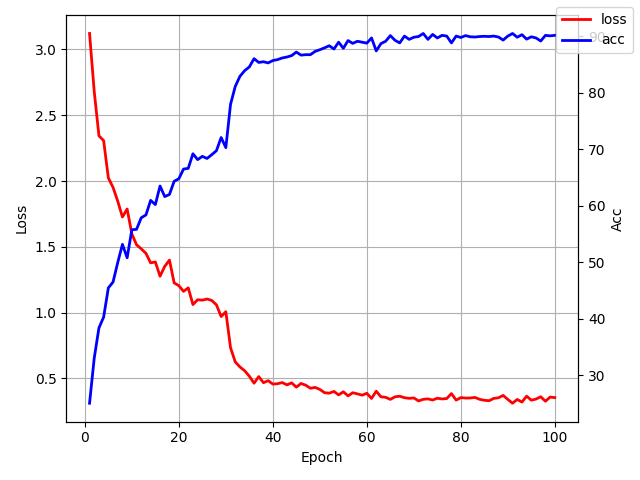

Supplement: S6 File — Numerical data underlying all figures and reported metrics, including complete training logs, evaluation results, per-class performance values, and confusion matrices. (ZIP) [file pone.0344262.s006.zip › Dataset/results/ResNet18/train_loss-acc.png]

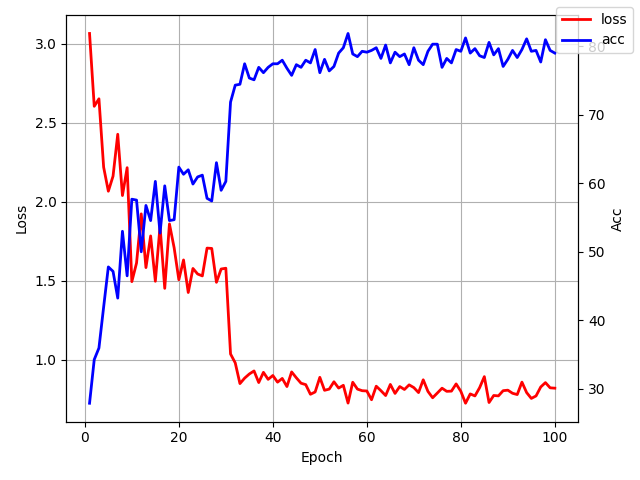

Supplement: S6 File — Numerical data underlying all figures and reported metrics, including complete training logs, evaluation results, per-class performance values, and confusion matrices. (ZIP) [file pone.0344262.s006.zip › Dataset/results/ResNet18/val_loss-acc.png]

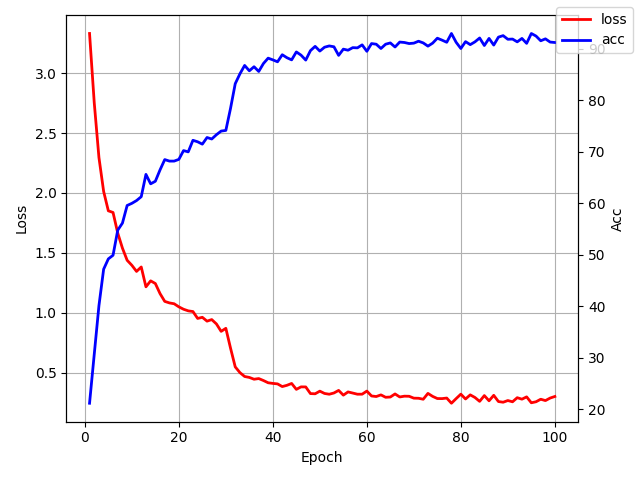

Supplement: S6 File — Numerical data underlying all figures and reported metrics, including complete training logs, evaluation results, per-class performance values, and confusion matrices. (ZIP) [file pone.0344262.s006.zip › Dataset/results/ResNet34/train_loss-acc.png]

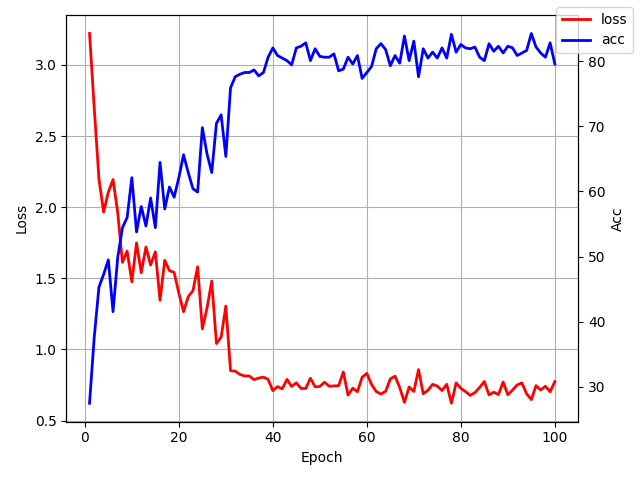

Supplement: S6 File — Numerical data underlying all figures and reported metrics, including complete training logs, evaluation results, per-class performance values, and confusion matrices. (ZIP) [file pone.0344262.s006.zip › Dataset/results/ResNet34/val_loss-acc.png]

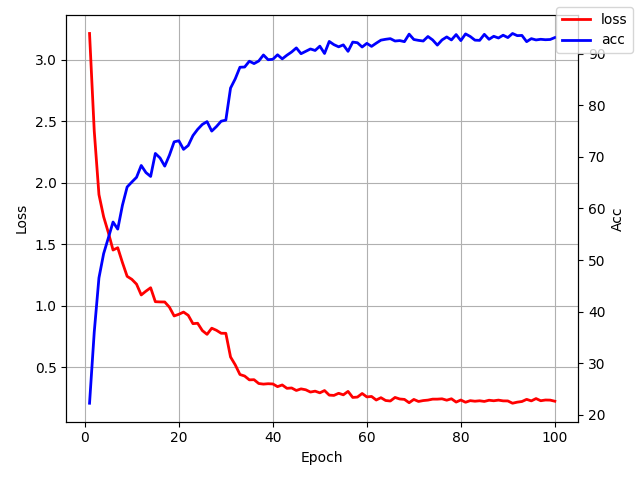

Supplement: S6 File — Numerical data underlying all figures and reported metrics, including complete training logs, evaluation results, per-class performance values, and confusion matrices. (ZIP) [file pone.0344262.s006.zip › Dataset/results/ResNet50/train_loss-acc.png]

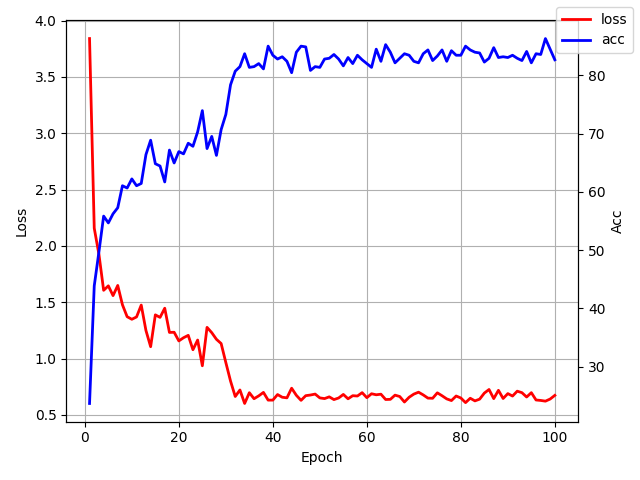

Supplement: S6 File — Numerical data underlying all figures and reported metrics, including complete training logs, evaluation results, per-class performance values, and confusion matrices. (ZIP) [file pone.0344262.s006.zip › Dataset/results/ResNet50/val_loss-acc.png]

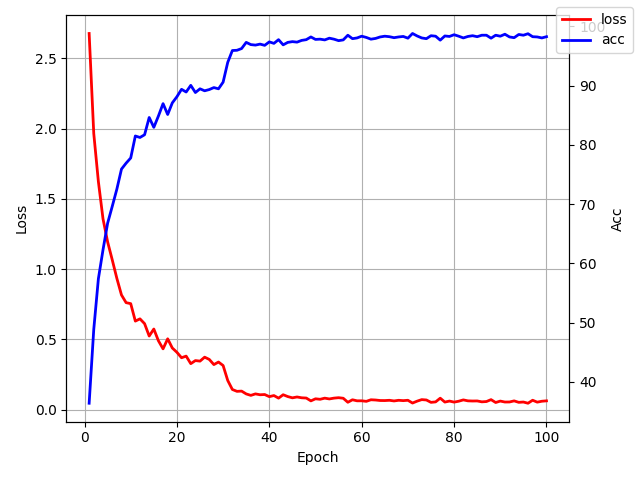

Supplement: S6 File — Numerical data underlying all figures and reported metrics, including complete training logs, evaluation results, per-class performance values, and confusion matrices. (ZIP) [file pone.0344262.s006.zip › Dataset/results/ResNext101/train_loss-acc.png]

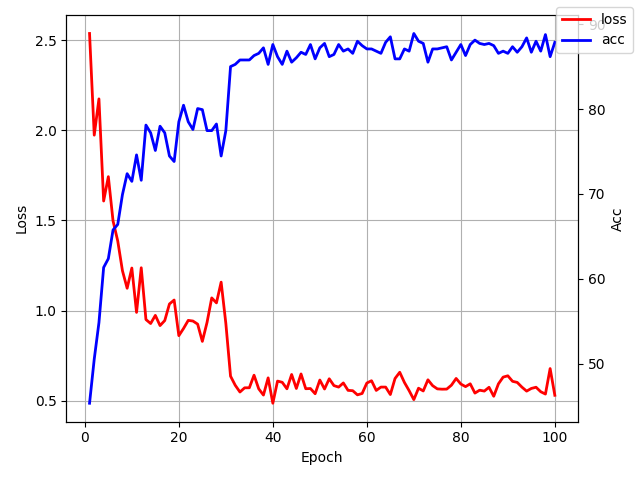

Supplement: S6 File — Numerical data underlying all figures and reported metrics, including complete training logs, evaluation results, per-class performance values, and confusion matrices. (ZIP) [file pone.0344262.s006.zip › Dataset/results/ResNext101/val_loss-acc.png]

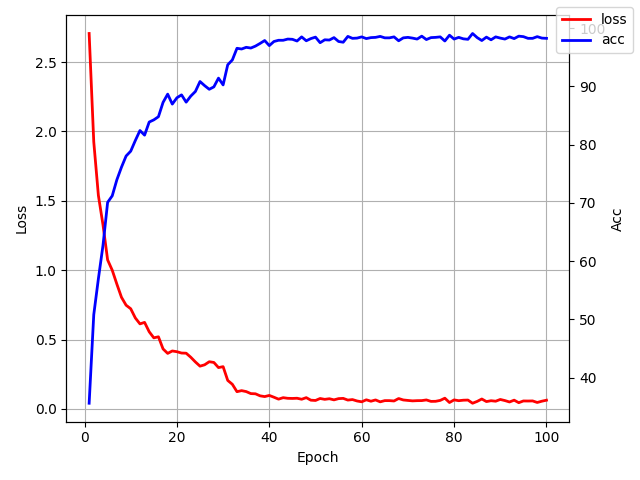

Supplement: S6 File — Numerical data underlying all figures and reported metrics, including complete training logs, evaluation results, per-class performance values, and confusion matrices. (ZIP) [file pone.0344262.s006.zip › Dataset/results/ResNext152/train_loss-acc.png]

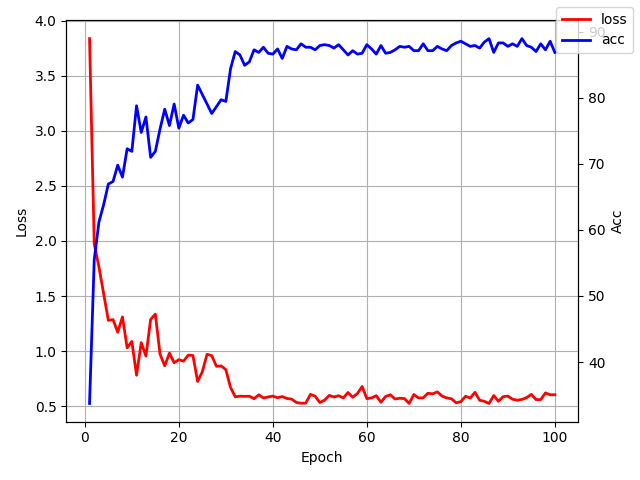

Supplement: S6 File — Numerical data underlying all figures and reported metrics, including complete training logs, evaluation results, per-class performance values, and confusion matrices. (ZIP) [file pone.0344262.s006.zip › Dataset/results/ResNext152/val_loss-acc.png]

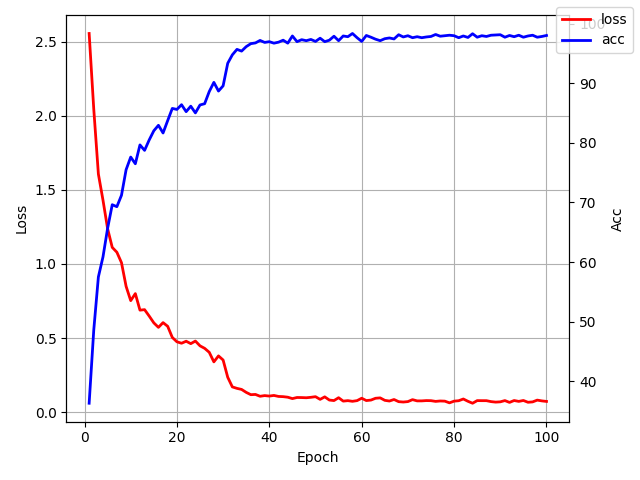

Supplement: S6 File — Numerical data underlying all figures and reported metrics, including complete training logs, evaluation results, per-class performance values, and confusion matrices. (ZIP) [file pone.0344262.s006.zip › Dataset/results/ResNext50/train_loss-acc.png]

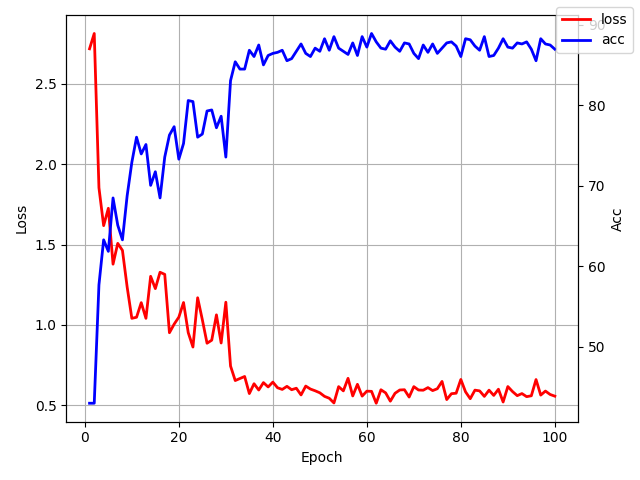

Supplement: S6 File — Numerical data underlying all figures and reported metrics, including complete training logs, evaluation results, per-class performance values, and confusion matrices. (ZIP) [file pone.0344262.s006.zip › Dataset/results/ResNext50/val_loss-acc.png]

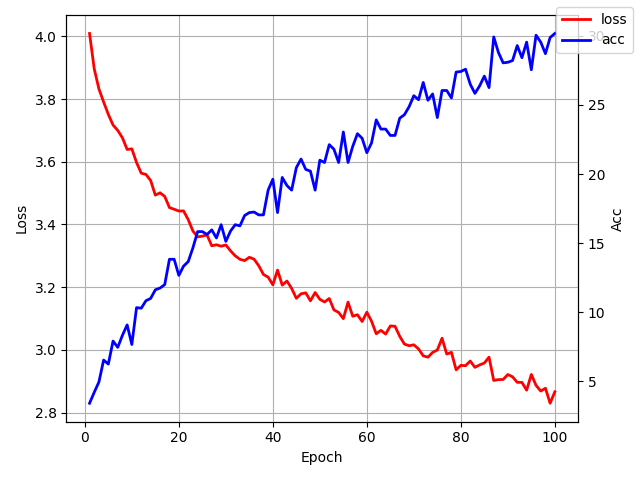

Supplement: S6 File — Numerical data underlying all figures and reported metrics, including complete training logs, evaluation results, per-class performance values, and confusion matrices. (ZIP) [file pone.0344262.s006.zip › Dataset/results/SwinTransformer/train_loss-acc.png]

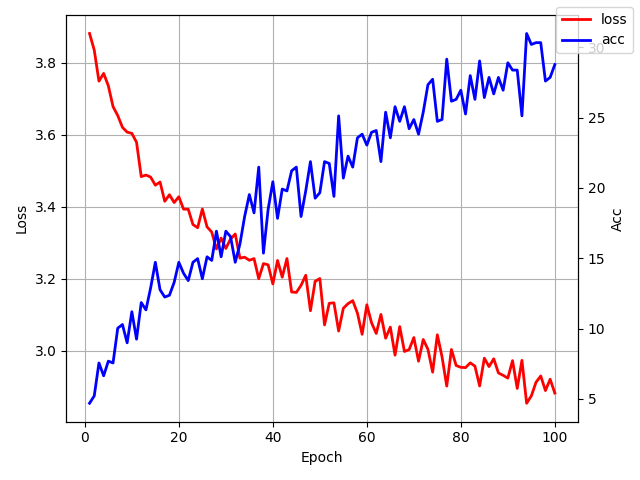

Supplement: S6 File — Numerical data underlying all figures and reported metrics, including complete training logs, evaluation results, per-class performance values, and confusion matrices. (ZIP) [file pone.0344262.s006.zip › Dataset/results/SwinTransformer/val_loss-acc.png]

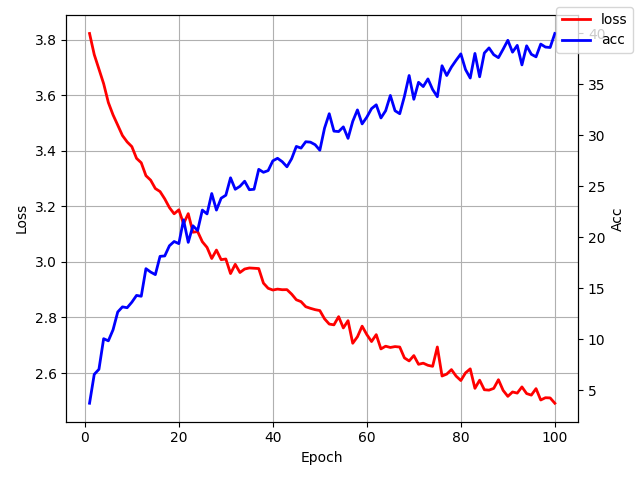

Supplement: S6 File — Numerical data underlying all figures and reported metrics, including complete training logs, evaluation results, per-class performance values, and confusion matrices. (ZIP) [file pone.0344262.s006.zip › Dataset/results/VisionTransformer/train_loss-acc.png]

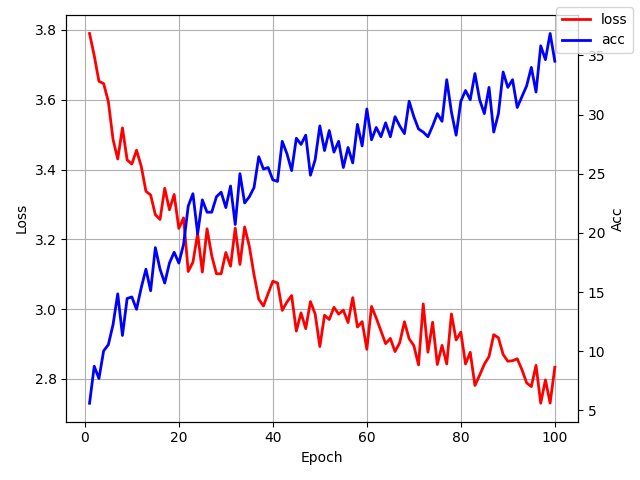

Supplement: S6 File — Numerical data underlying all figures and reported metrics, including complete training logs, evaluation results, per-class performance values, and confusion matrices. (ZIP) [file pone.0344262.s006.zip › Dataset/results/VisionTransformer/val_loss-acc.png]

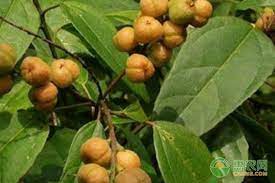

Supplement: S6 File — Numerical data underlying all figures and reported metrics, including complete training logs, evaluation results, per-class performance values, and confusion matrices. (ZIP) [file pone.0344262.s006.zip › Dataset/sample_images/badou/(2).jpg]

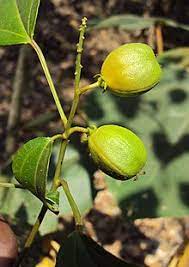

Supplement: S6 File — Numerical data underlying all figures and reported metrics, including complete training logs, evaluation results, per-class performance values, and confusion matrices. (ZIP) [file pone.0344262.s006.zip › Dataset/sample_images/badou/(3).jpg]

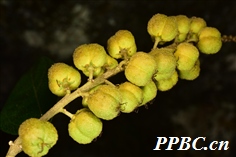

Supplement: S6 File — Numerical data underlying all figures and reported metrics, including complete training logs, evaluation results, per-class performance values, and confusion matrices. (ZIP) [file pone.0344262.s006.zip › Dataset/sample_images/badou/10926397.jpg]

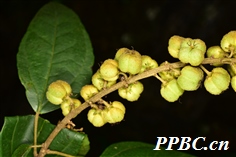

Supplement: S6 File — Numerical data underlying all figures and reported metrics, including complete training logs, evaluation results, per-class performance values, and confusion matrices. (ZIP) [file pone.0344262.s006.zip › Dataset/sample_images/badou/10926402.jpg]

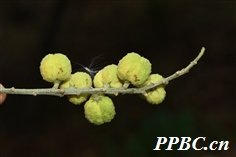

Supplement: S6 File — Numerical data underlying all figures and reported metrics, including complete training logs, evaluation results, per-class performance values, and confusion matrices. (ZIP) [file pone.0344262.s006.zip › Dataset/sample_images/badou/11513321.jpg]

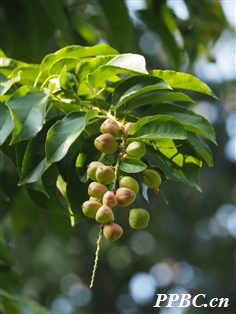

Supplement: S6 File — Numerical data underlying all figures and reported metrics, including complete training logs, evaluation results, per-class performance values, and confusion matrices. (ZIP) [file pone.0344262.s006.zip › Dataset/sample_images/badou/11705961.jpg]

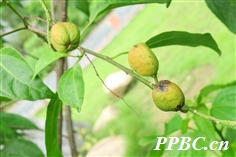

Supplement: S6 File — Numerical data underlying all figures and reported metrics, including complete training logs, evaluation results, per-class performance values, and confusion matrices. (ZIP) [file pone.0344262.s006.zip › Dataset/sample_images/badou/2851934.jpg]

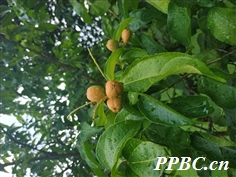

Supplement: S6 File — Numerical data underlying all figures and reported metrics, including complete training logs, evaluation results, per-class performance values, and confusion matrices. (ZIP) [file pone.0344262.s006.zip › Dataset/sample_images/badou/5476877.jpg]

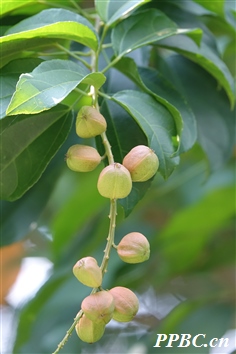

Supplement: S6 File — Numerical data underlying all figures and reported metrics, including complete training logs, evaluation results, per-class performance values, and confusion matrices. (ZIP) [file pone.0344262.s006.zip › Dataset/sample_images/badou/5777199.jpg]

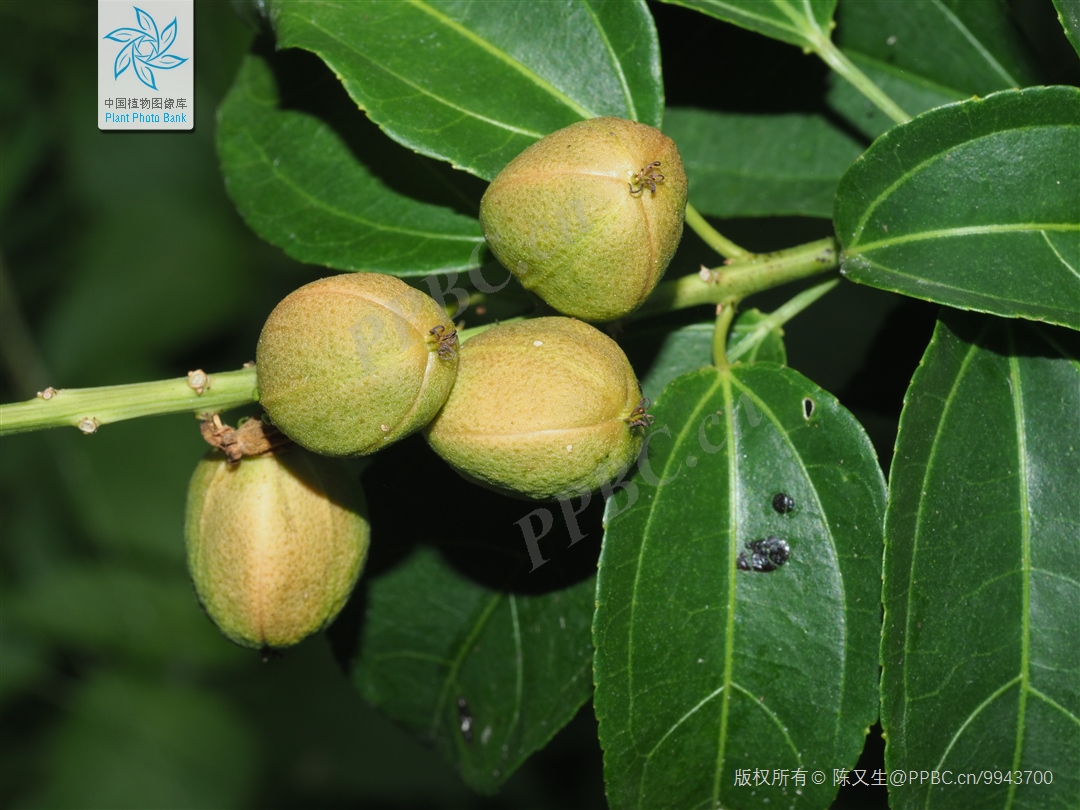

Supplement: S6 File — Numerical data underlying all figures and reported metrics, including complete training logs, evaluation results, per-class performance values, and confusion matrices. (ZIP) [file pone.0344262.s006.zip › Dataset/sample_images/badou/9943700.jpg]

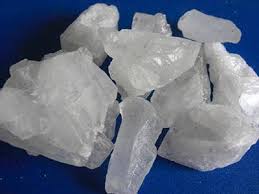

Supplement: S6 File — Numerical data underlying all figures and reported metrics, including complete training logs, evaluation results, per-class performance values, and confusion matrices. (ZIP) [file pone.0344262.s006.zip › Dataset/sample_images/baifan/25.jpg]

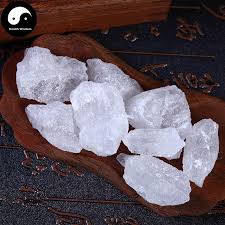

Supplement: S6 File — Numerical data underlying all figures and reported metrics, including complete training logs, evaluation results, per-class performance values, and confusion matrices. (ZIP) [file pone.0344262.s006.zip › Dataset/sample_images/baifan/32.jpg]

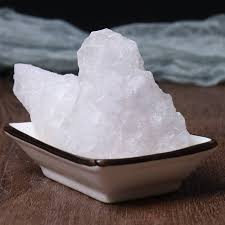

Supplement: S6 File — Numerical data underlying all figures and reported metrics, including complete training logs, evaluation results, per-class performance values, and confusion matrices. (ZIP) [file pone.0344262.s006.zip › Dataset/sample_images/baifan/35.jpg]

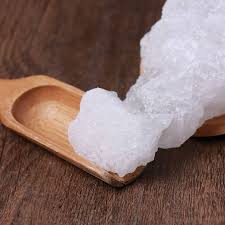

Supplement: S6 File — Numerical data underlying all figures and reported metrics, including complete training logs, evaluation results, per-class performance values, and confusion matrices. (ZIP) [file pone.0344262.s006.zip › Dataset/sample_images/baifan/39.jpg]

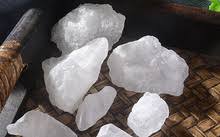

Supplement: S6 File — Numerical data underlying all figures and reported metrics, including complete training logs, evaluation results, per-class performance values, and confusion matrices. (ZIP) [file pone.0344262.s006.zip › Dataset/sample_images/baifan/images (11).jpg]

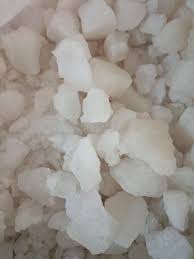

Supplement: S6 File — Numerical data underlying all figures and reported metrics, including complete training logs, evaluation results, per-class performance values, and confusion matrices. (ZIP) [file pone.0344262.s006.zip › Dataset/sample_images/baifan/images (15).jpg]

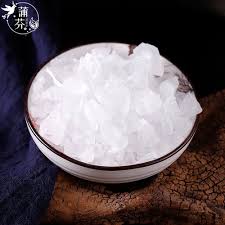

Supplement: S6 File — Numerical data underlying all figures and reported metrics, including complete training logs, evaluation results, per-class performance values, and confusion matrices. (ZIP) [file pone.0344262.s006.zip › Dataset/sample_images/baifan/images (7).jpg]

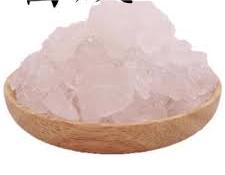

Supplement: S6 File — Numerical data underlying all figures and reported metrics, including complete training logs, evaluation results, per-class performance values, and confusion matrices. (ZIP) [file pone.0344262.s006.zip › Dataset/sample_images/baifan/images (9).jpg]

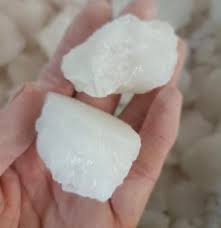

Supplement: S6 File — Numerical data underlying all figures and reported metrics, including complete training logs, evaluation results, per-class performance values, and confusion matrices. (ZIP) [file pone.0344262.s006.zip › Dataset/sample_images/baifan/images3.jpg]

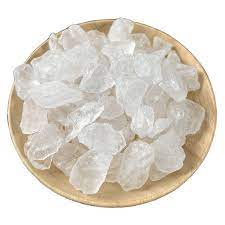

Supplement: S6 File — Numerical data underlying all figures and reported metrics, including complete training logs, evaluation results, per-class performance values, and confusion matrices. (ZIP) [file pone.0344262.s006.zip › Dataset/sample_images/baifan/images6.jpg]

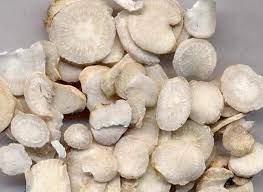

Supplement: S6 File — Numerical data underlying all figures and reported metrics, including complete training logs, evaluation results, per-class performance values, and confusion matrices. (ZIP) [file pone.0344262.s006.zip › Dataset/sample_images/baifuzi/1.jpg]

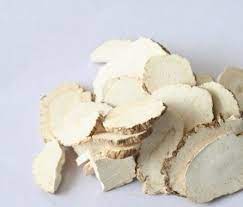

Supplement: S6 File — Numerical data underlying all figures and reported metrics, including complete training logs, evaluation results, per-class performance values, and confusion matrices. (ZIP) [file pone.0344262.s006.zip › Dataset/sample_images/baifuzi/10.jpg]

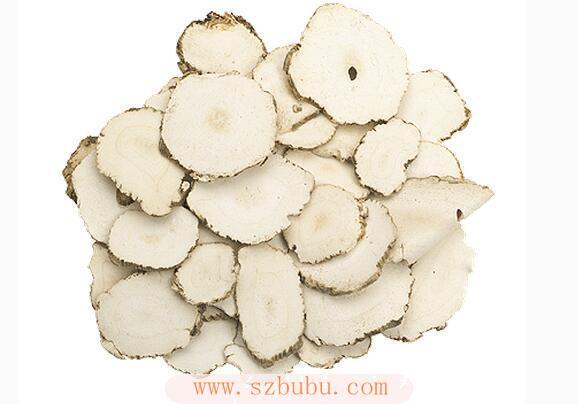

Supplement: S6 File — Numerical data underlying all figures and reported metrics, including complete training logs, evaluation results, per-class performance values, and confusion matrices. (ZIP) [file pone.0344262.s006.zip › Dataset/sample_images/baifuzi/1b6eb97e59cdcf06027f4fdc0f82bb72.jpeg]

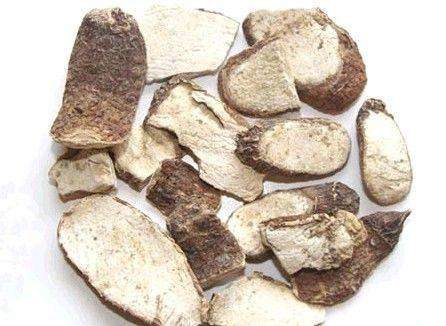

Supplement: S6 File — Numerical data underlying all figures and reported metrics, including complete training logs, evaluation results, per-class performance values, and confusion matrices. (ZIP) [file pone.0344262.s006.zip › Dataset/sample_images/baifuzi/286849436fac872a6c4847e7535b24bc.jpeg]

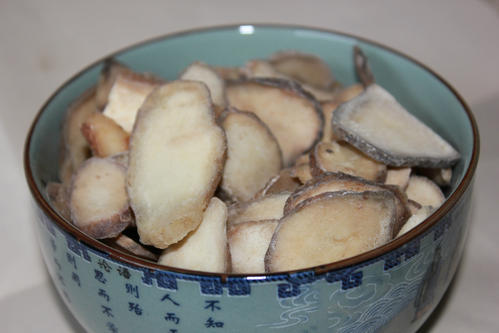

Supplement: S6 File — Numerical data underlying all figures and reported metrics, including complete training logs, evaluation results, per-class performance values, and confusion matrices. (ZIP) [file pone.0344262.s006.zip › Dataset/sample_images/baifuzi/b84500b63537fd5eca176bb83bb98c94.jpg]

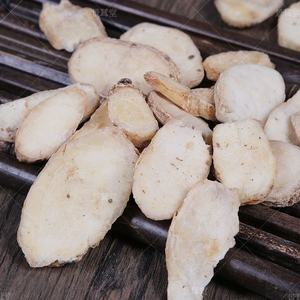

Supplement: S6 File — Numerical data underlying all figures and reported metrics, including complete training logs, evaluation results, per-class performance values, and confusion matrices. (ZIP) [file pone.0344262.s006.zip › Dataset/sample_images/baifuzi/daba3371049a262237ff4bd79ac1e906.jpeg]

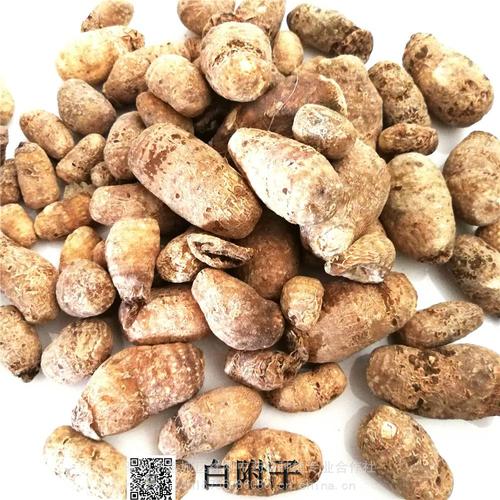

Supplement: S6 File — Numerical data underlying all figures and reported metrics, including complete training logs, evaluation results, per-class performance values, and confusion matrices. (ZIP) [file pone.0344262.s006.zip › Dataset/sample_images/baifuzi/e3ad0febcaac8eea16cfa9ec27fc0062.jpeg]

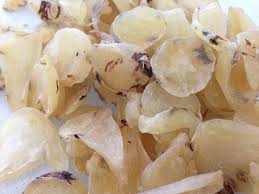

Supplement: S6 File — Numerical data underlying all figures and reported metrics, including complete training logs, evaluation results, per-class performance values, and confusion matrices. (ZIP) [file pone.0344262.s006.zip › Dataset/sample_images/baifuzi/images (1).jpg]

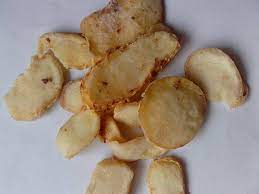

Supplement: S6 File — Numerical data underlying all figures and reported metrics, including complete training logs, evaluation results, per-class performance values, and confusion matrices. (ZIP) [file pone.0344262.s006.zip › Dataset/sample_images/baifuzi/images (10).jpg]

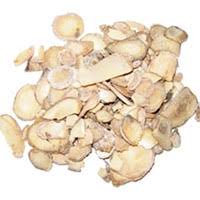

Supplement: S6 File — Numerical data underlying all figures and reported metrics, including complete training logs, evaluation results, per-class performance values, and confusion matrices. (ZIP) [file pone.0344262.s006.zip › Dataset/sample_images/baifuzi/images (5).jpg]

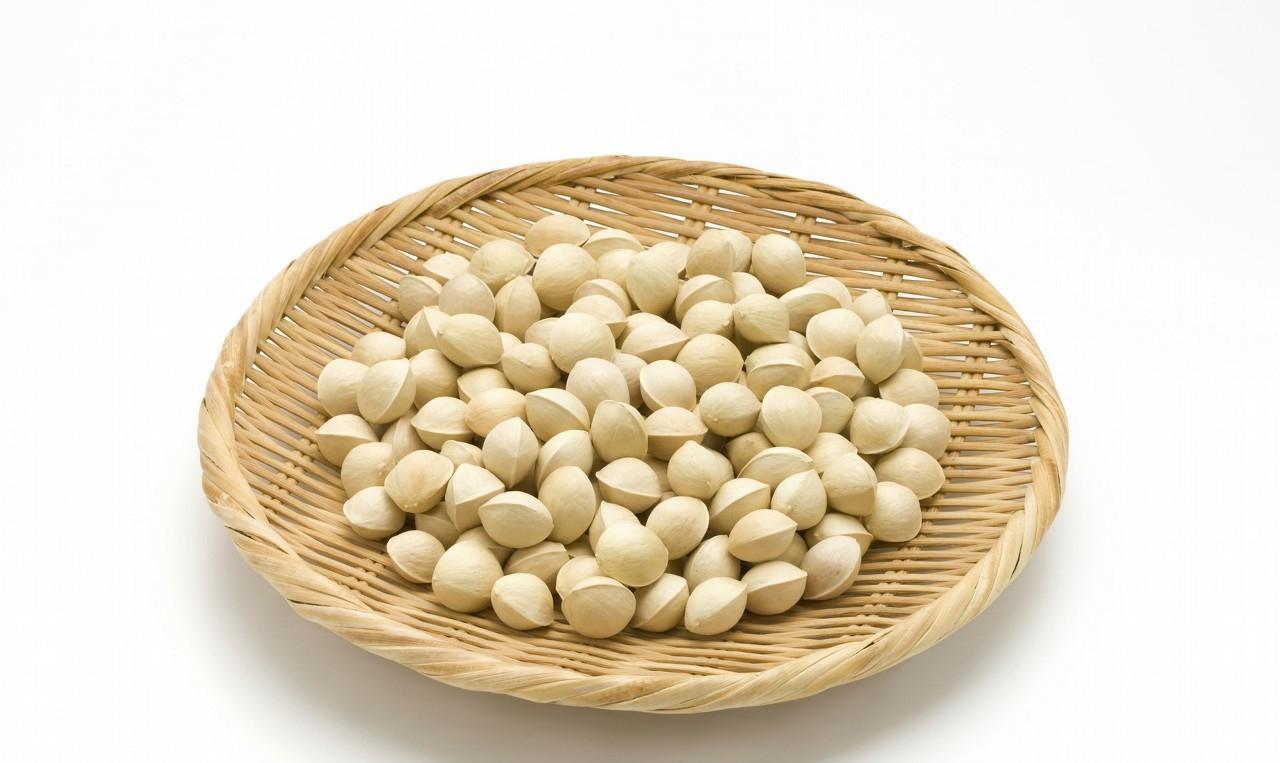

Supplement: S6 File — Numerical data underlying all figures and reported metrics, including complete training logs, evaluation results, per-class performance values, and confusion matrices. (ZIP) [file pone.0344262.s006.zip › Dataset/sample_images/baiguo/1d09b95048237c1dc1beeb729c5ca932.jpg]

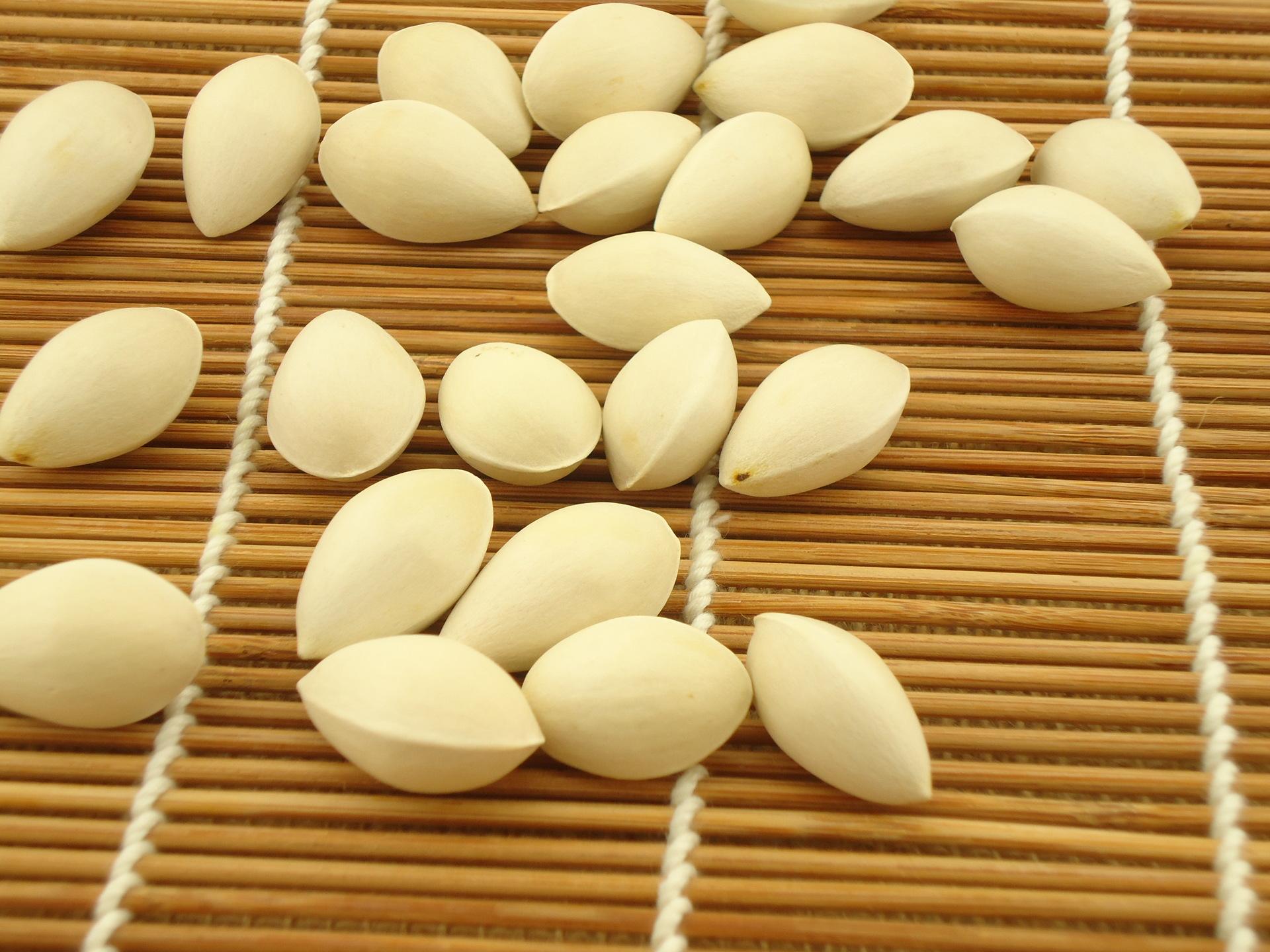

Supplement: S6 File — Numerical data underlying all figures and reported metrics, including complete training logs, evaluation results, per-class performance values, and confusion matrices. (ZIP) [file pone.0344262.s006.zip › Dataset/sample_images/baiguo/383966d296e93ed9daa67cb4291ac428.jpeg]

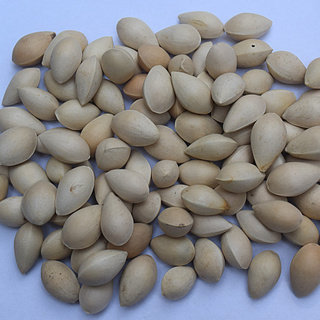

Supplement: S6 File — Numerical data underlying all figures and reported metrics, including complete training logs, evaluation results, per-class performance values, and confusion matrices. (ZIP) [file pone.0344262.s006.zip › Dataset/sample_images/baiguo/6044dd99e8e02ae110305fd468962045.jpg]

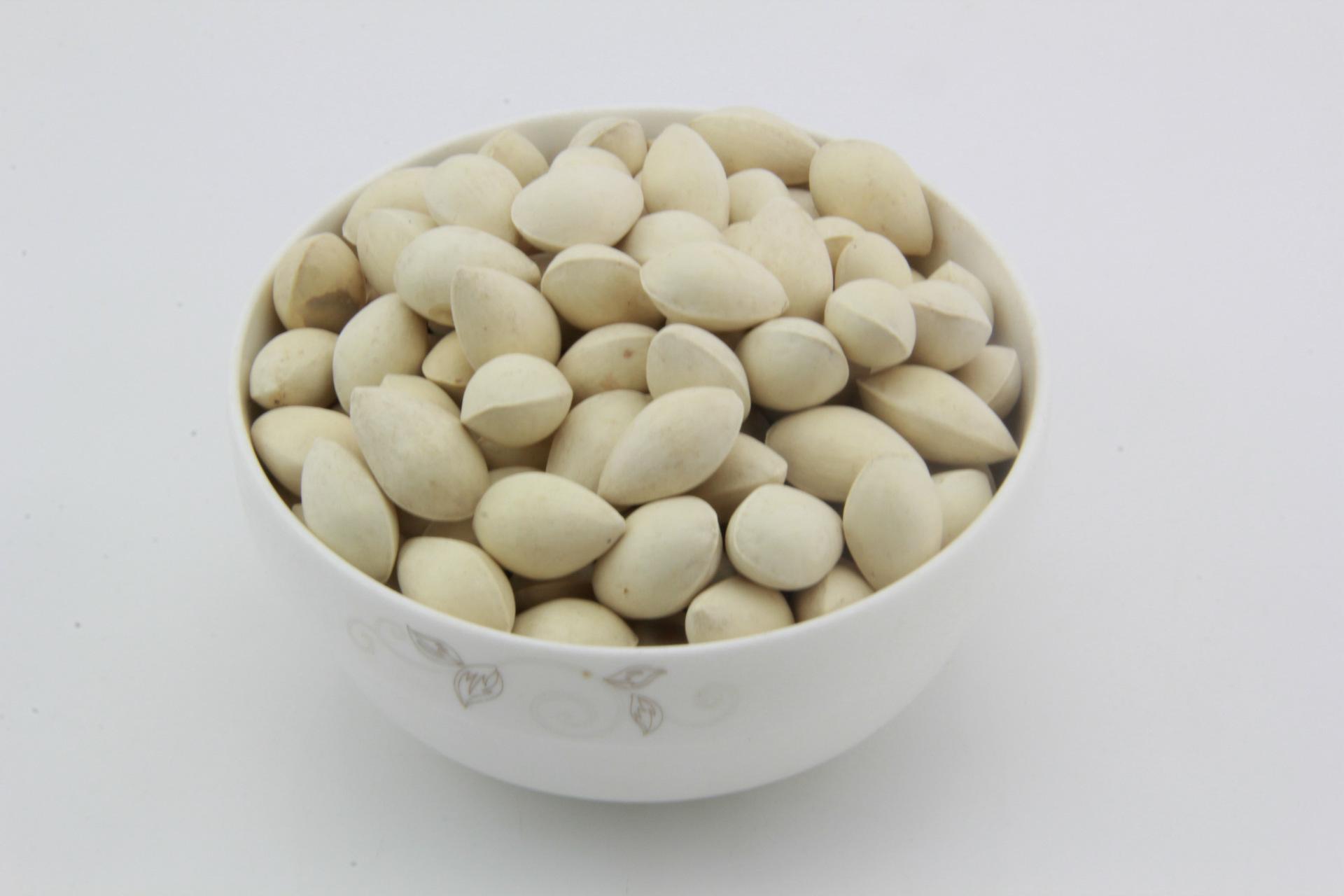

Supplement: S6 File — Numerical data underlying all figures and reported metrics, including complete training logs, evaluation results, per-class performance values, and confusion matrices. (ZIP) [file pone.0344262.s006.zip › Dataset/sample_images/baiguo/67903d69f495755516a741bbed9f7410.jpeg]

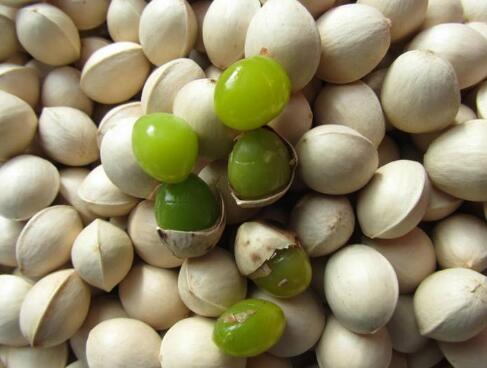

Supplement: S6 File — Numerical data underlying all figures and reported metrics, including complete training logs, evaluation results, per-class performance values, and confusion matrices. (ZIP) [file pone.0344262.s006.zip › Dataset/sample_images/baiguo/9100468eeb239f16e6ea8dff496a40bf.jpg]

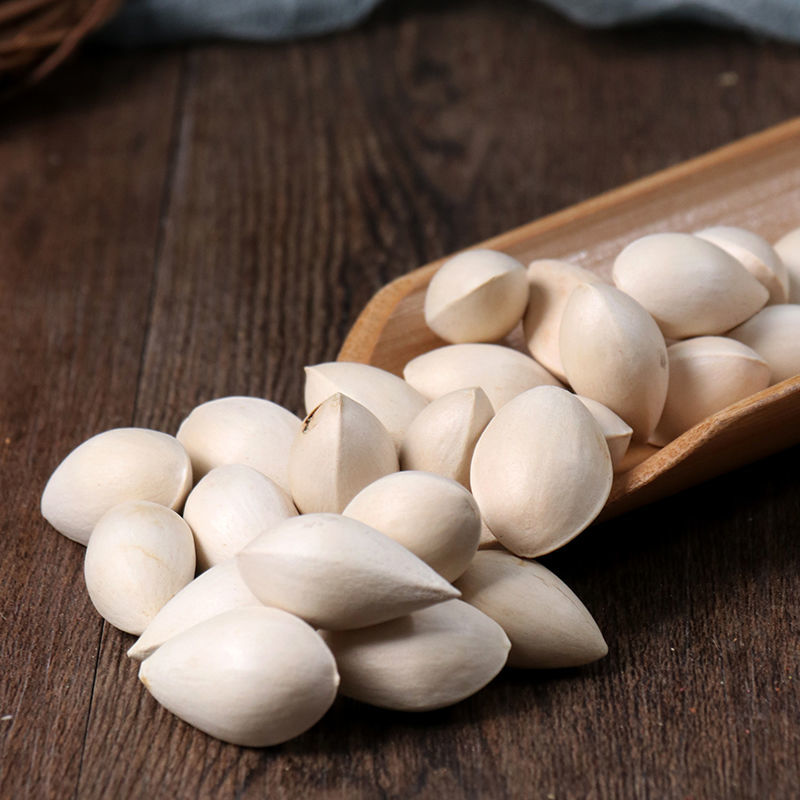

Supplement: S6 File — Numerical data underlying all figures and reported metrics, including complete training logs, evaluation results, per-class performance values, and confusion matrices. (ZIP) [file pone.0344262.s006.zip › Dataset/sample_images/baiguo/9f955e4dea0ff4de5a64c77f5bc38c82.jpg]

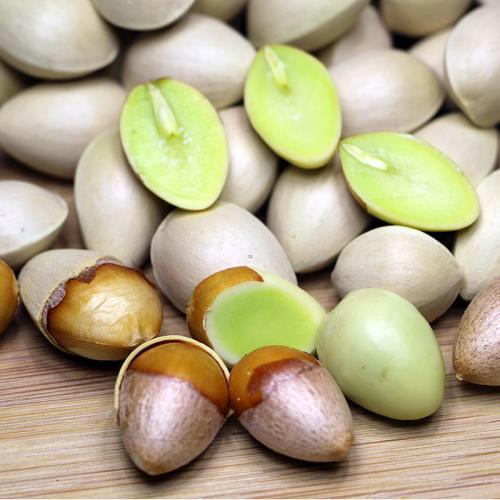

Supplement: S6 File — Numerical data underlying all figures and reported metrics, including complete training logs, evaluation results, per-class performance values, and confusion matrices. (ZIP) [file pone.0344262.s006.zip › Dataset/sample_images/baiguo/a2703a4283ae31463222dc9f15279dff.jpeg]

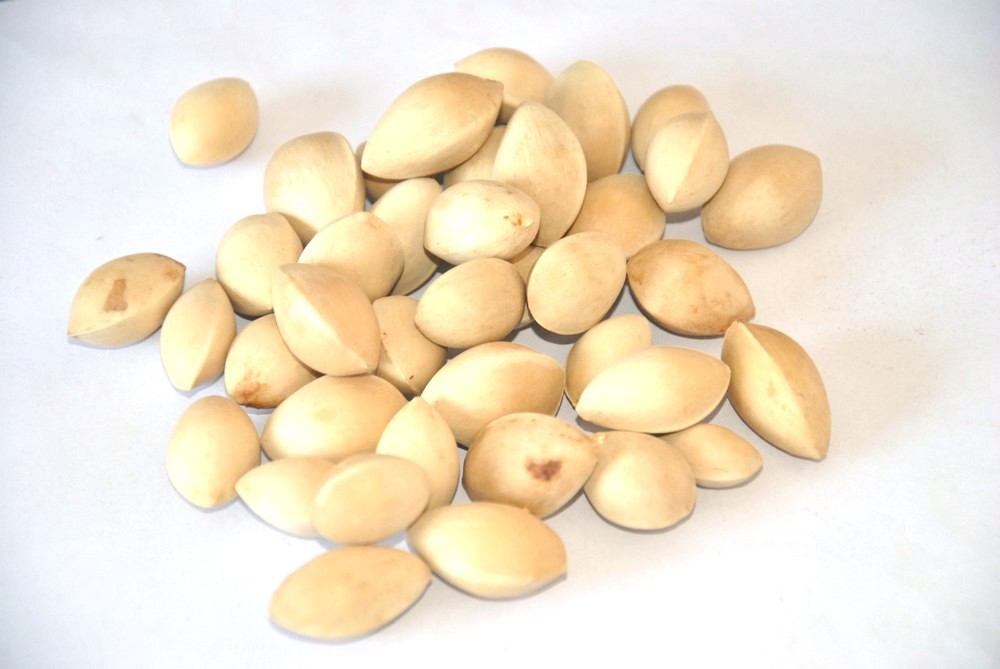

Supplement: S6 File — Numerical data underlying all figures and reported metrics, including complete training logs, evaluation results, per-class performance values, and confusion matrices. (ZIP) [file pone.0344262.s006.zip › Dataset/sample_images/baiguo/af52c137d82d194c7f03fac6c59655b5.jpg]

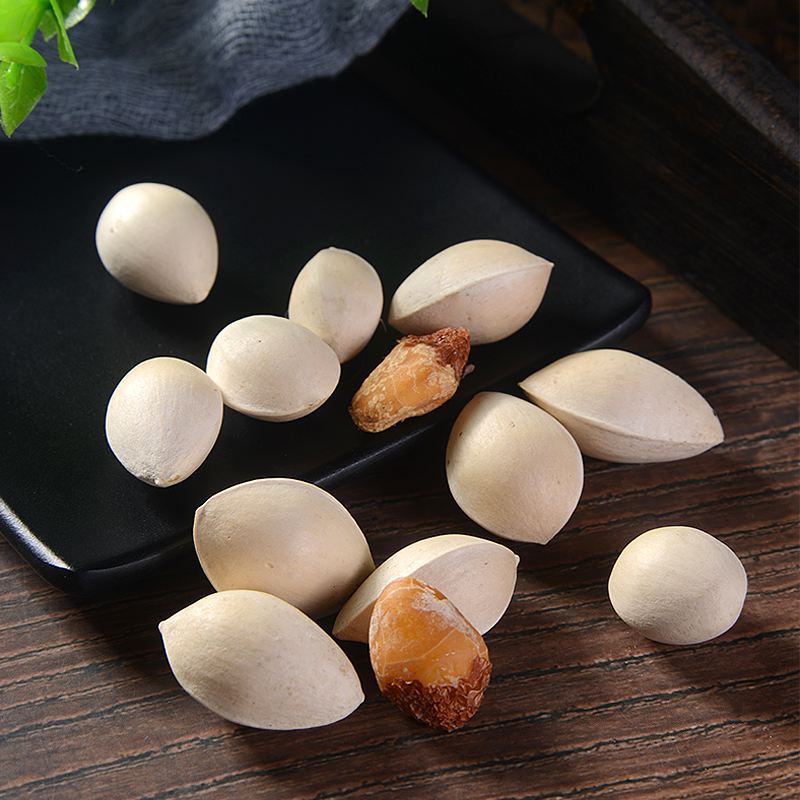

Supplement: S6 File — Numerical data underlying all figures and reported metrics, including complete training logs, evaluation results, per-class performance values, and confusion matrices. (ZIP) [file pone.0344262.s006.zip › Dataset/sample_images/baiguo/c124392e49835008786177bd5302ad5f.jpeg]

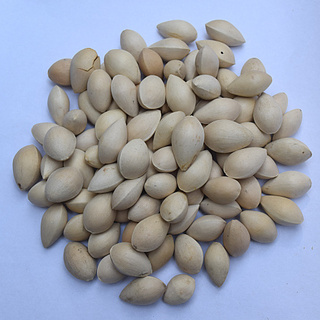

Supplement: S6 File — Numerical data underlying all figures and reported metrics, including complete training logs, evaluation results, per-class performance values, and confusion matrices. (ZIP) [file pone.0344262.s006.zip › Dataset/sample_images/baiguo/c92758bf33904006c9f057dbf7187dc7.jpg]

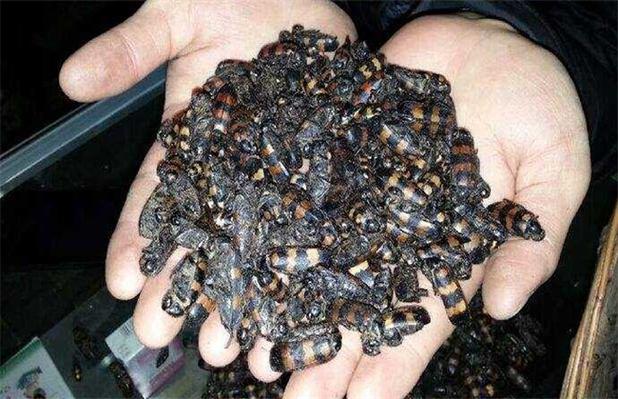

Supplement: S6 File — Numerical data underlying all figures and reported metrics, including complete training logs, evaluation results, per-class performance values, and confusion matrices. (ZIP) [file pone.0344262.s006.zip › Dataset/sample_images/banmao/0b3ed9fceb384440085a1b7dc891e129.jpeg]

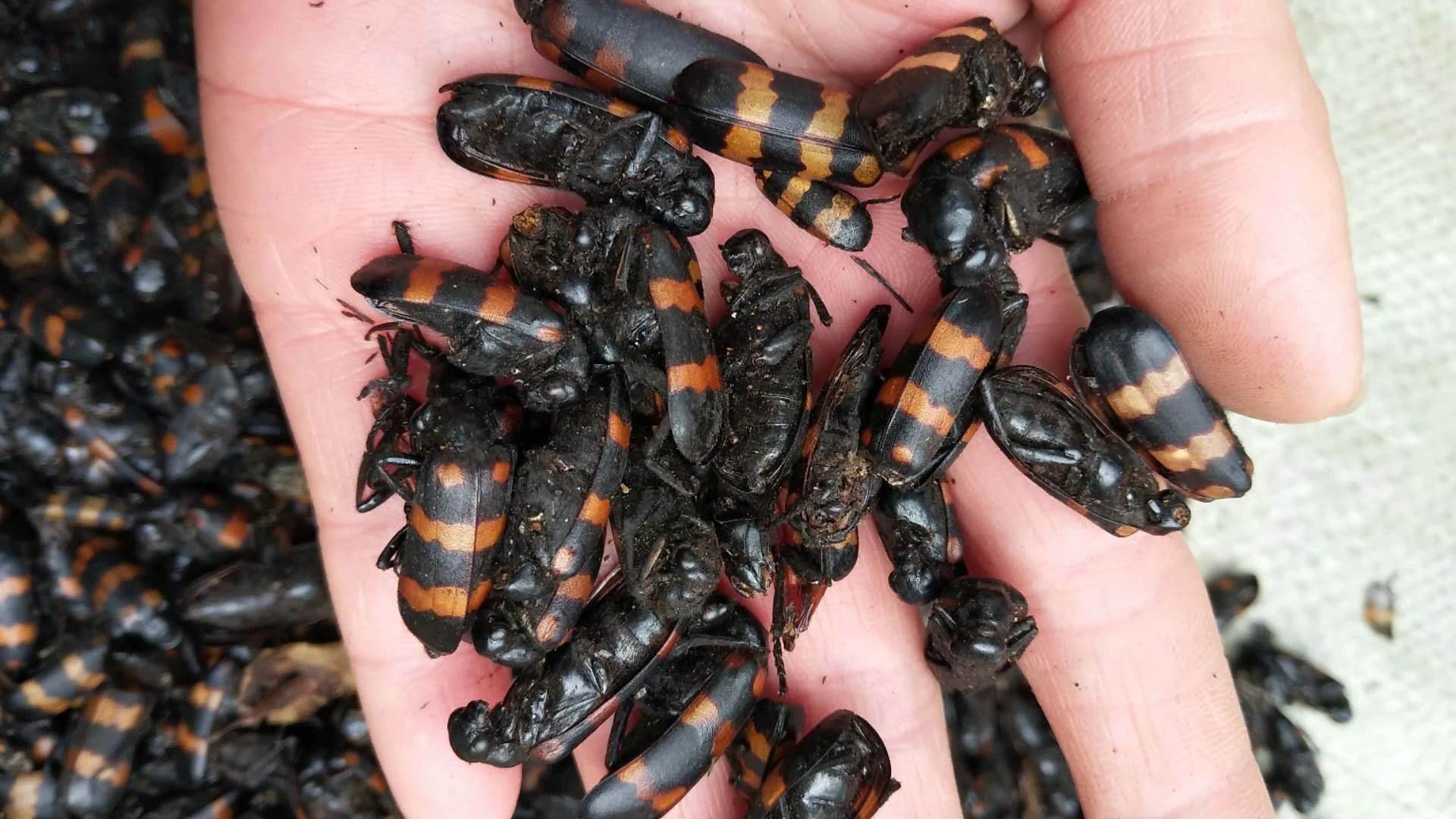

Supplement: S6 File — Numerical data underlying all figures and reported metrics, including complete training logs, evaluation results, per-class performance values, and confusion matrices. (ZIP) [file pone.0344262.s006.zip › Dataset/sample_images/banmao/25fbe725e12177522da3e40740f9c523.jpg]

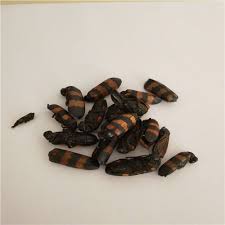

Supplement: S6 File — Numerical data underlying all figures and reported metrics, including complete training logs, evaluation results, per-class performance values, and confusion matrices. (ZIP) [file pone.0344262.s006.zip › Dataset/sample_images/banmao/29.jpg]

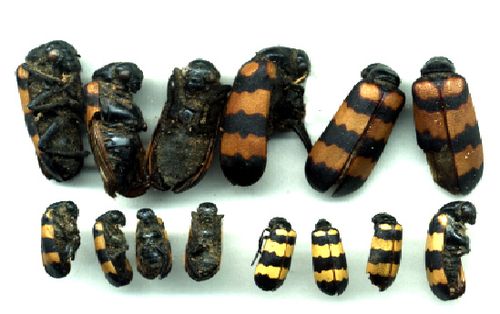

Supplement: S6 File — Numerical data underlying all figures and reported metrics, including complete training logs, evaluation results, per-class performance values, and confusion matrices. (ZIP) [file pone.0344262.s006.zip › Dataset/sample_images/banmao/2fb3a83ddb7a6cf22f85f07b3e2b9c63.jpeg]

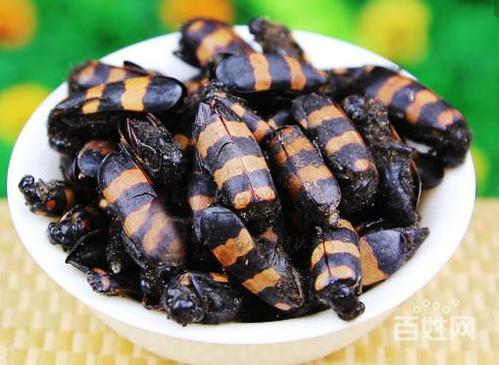

Supplement: S6 File — Numerical data underlying all figures and reported metrics, including complete training logs, evaluation results, per-class performance values, and confusion matrices. (ZIP) [file pone.0344262.s006.zip › Dataset/sample_images/banmao/330efe7fca873eab7b5d6f8762964f1c.jpeg]

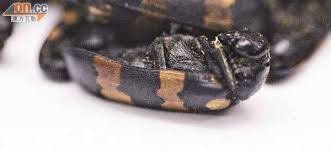

Supplement: S6 File — Numerical data underlying all figures and reported metrics, including complete training logs, evaluation results, per-class performance values, and confusion matrices. (ZIP) [file pone.0344262.s006.zip › Dataset/sample_images/banmao/38.jpg]

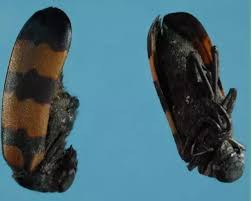

Supplement: S6 File — Numerical data underlying all figures and reported metrics, including complete training logs, evaluation results, per-class performance values, and confusion matrices. (ZIP) [file pone.0344262.s006.zip › Dataset/sample_images/banmao/56.jpg]

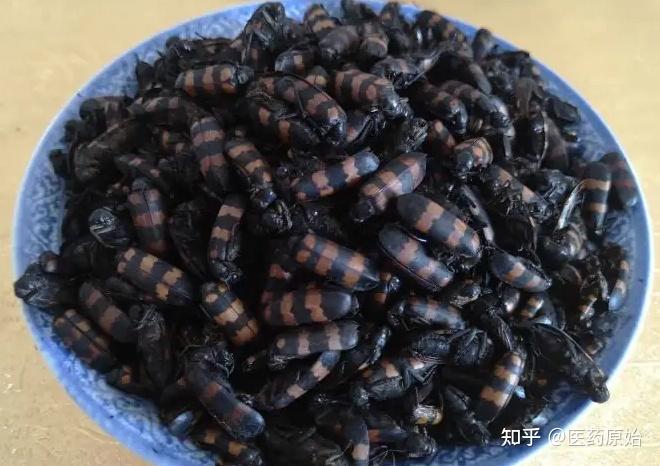

Supplement: S6 File — Numerical data underlying all figures and reported metrics, including complete training logs, evaluation results, per-class performance values, and confusion matrices. (ZIP) [file pone.0344262.s006.zip › Dataset/sample_images/banmao/8536fdfe7c0b1365e087b7c63897ad00.jpg]

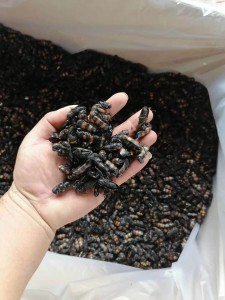

Supplement: S6 File — Numerical data underlying all figures and reported metrics, including complete training logs, evaluation results, per-class performance values, and confusion matrices. (ZIP) [file pone.0344262.s006.zip › Dataset/sample_images/banmao/bb33e5a06da1874946183833149400dd.jpeg]

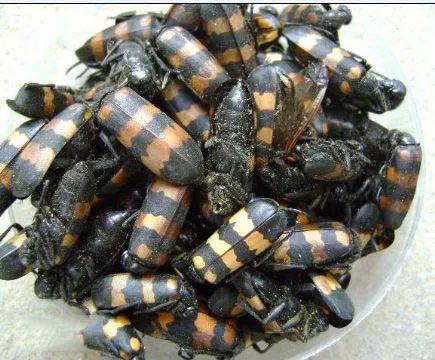

Supplement: S6 File — Numerical data underlying all figures and reported metrics, including complete training logs, evaluation results, per-class performance values, and confusion matrices. (ZIP) [file pone.0344262.s006.zip › Dataset/sample_images/banmao/eda6d56d24679c3222cb97a2f8bbda6f.jpeg]

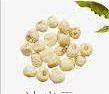

Supplement: S6 File — Numerical data underlying all figures and reported metrics, including complete training logs, evaluation results, per-class performance values, and confusion matrices. (ZIP) [file pone.0344262.s006.zip › Dataset/sample_images/banxia/10.jpg]

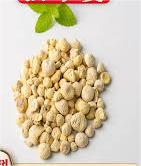

Supplement: S6 File — Numerical data underlying all figures and reported metrics, including complete training logs, evaluation results, per-class performance values, and confusion matrices. (ZIP) [file pone.0344262.s006.zip › Dataset/sample_images/banxia/24.jpg]

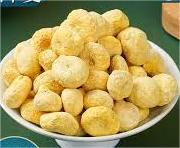

Supplement: S6 File — Numerical data underlying all figures and reported metrics, including complete training logs, evaluation results, per-class performance values, and confusion matrices. (ZIP) [file pone.0344262.s006.zip › Dataset/sample_images/banxia/25.jpg]

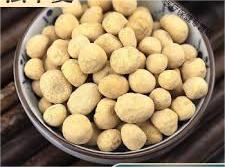

Supplement: S6 File — Numerical data underlying all figures and reported metrics, including complete training logs, evaluation results, per-class performance values, and confusion matrices. (ZIP) [file pone.0344262.s006.zip › Dataset/sample_images/banxia/33.jpg]

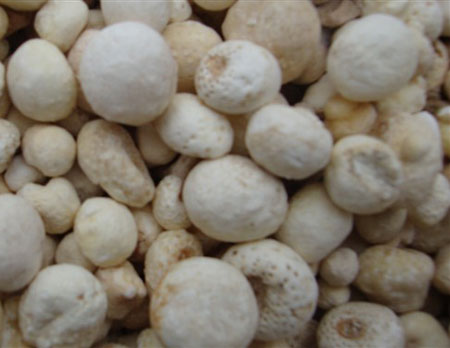

Supplement: S6 File — Numerical data underlying all figures and reported metrics, including complete training logs, evaluation results, per-class performance values, and confusion matrices. (ZIP) [file pone.0344262.s006.zip › Dataset/sample_images/banxia/34e0001cbaf9c083a29f6a3a0541817b.jpeg]

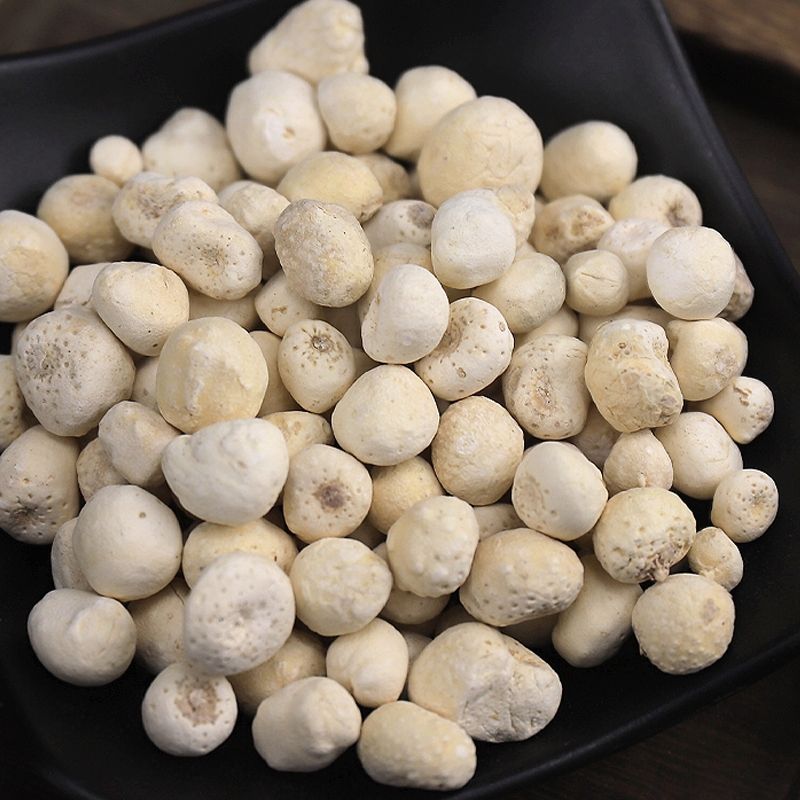

Supplement: S6 File — Numerical data underlying all figures and reported metrics, including complete training logs, evaluation results, per-class performance values, and confusion matrices. (ZIP) [file pone.0344262.s006.zip › Dataset/sample_images/banxia/38d37a216173b72d37ee8fedb8b10d4e.jpeg]

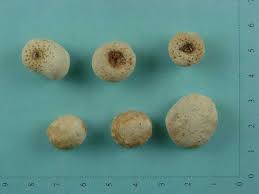

Supplement: S6 File — Numerical data underlying all figures and reported metrics, including complete training logs, evaluation results, per-class performance values, and confusion matrices. (ZIP) [file pone.0344262.s006.zip › Dataset/sample_images/banxia/41.jpg]

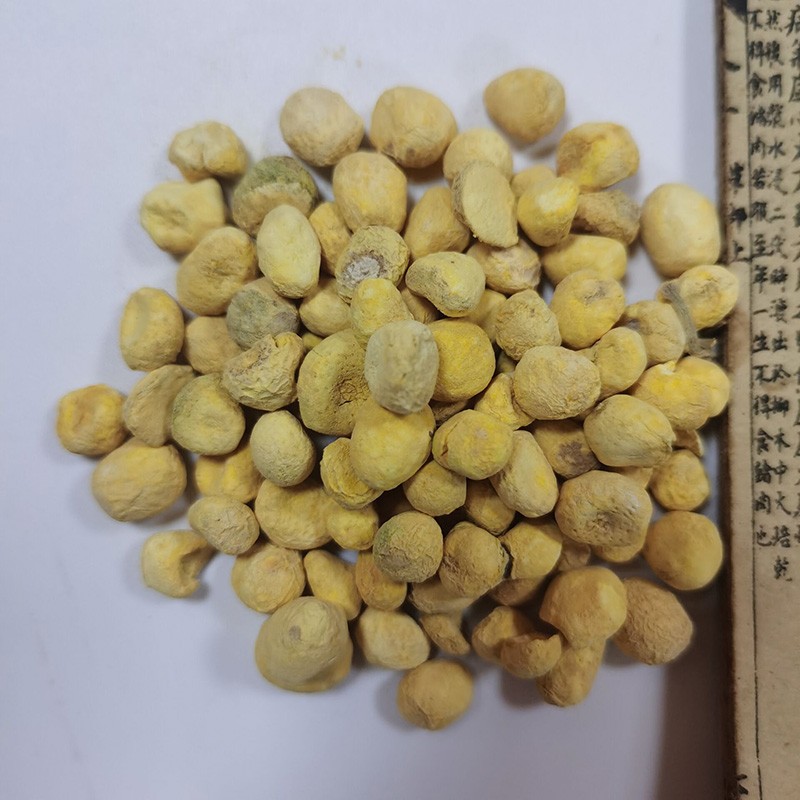

Supplement: S6 File — Numerical data underlying all figures and reported metrics, including complete training logs, evaluation results, per-class performance values, and confusion matrices. (ZIP) [file pone.0344262.s006.zip › Dataset/sample_images/banxia/5753008f15efb1335370d12c8401a979.jpg]

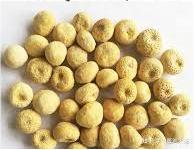

Supplement: S6 File — Numerical data underlying all figures and reported metrics, including complete training logs, evaluation results, per-class performance values, and confusion matrices. (ZIP) [file pone.0344262.s006.zip › Dataset/sample_images/banxia/9.jpg]

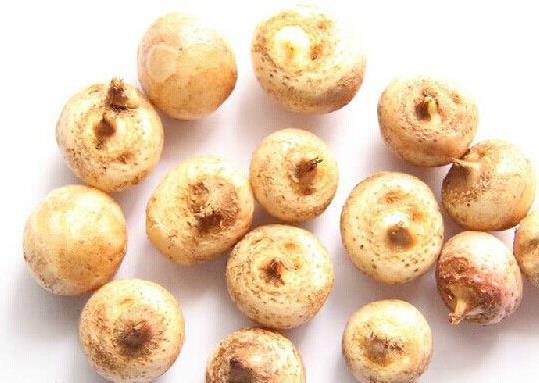

Supplement: S6 File — Numerical data underlying all figures and reported metrics, including complete training logs, evaluation results, per-class performance values, and confusion matrices. (ZIP) [file pone.0344262.s006.zip › Dataset/sample_images/banxia/fd6b2bb6a6fd68ab97d2d8c698311975.jpg]

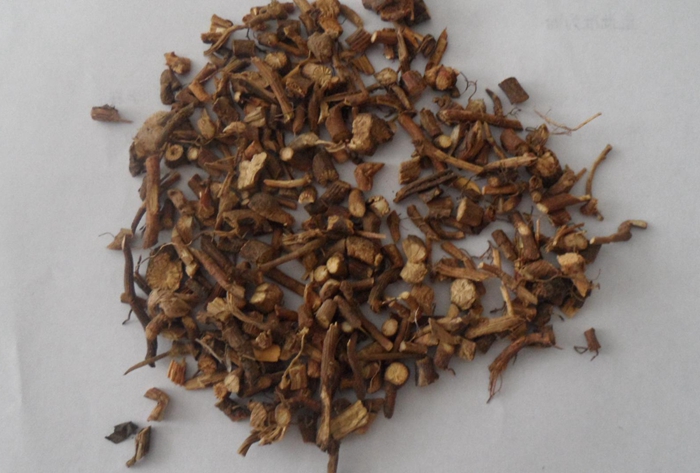

Supplement: S6 File — Numerical data underlying all figures and reported metrics, including complete training logs, evaluation results, per-class performance values, and confusion matrices. (ZIP) [file pone.0344262.s006.zip › Dataset/sample_images/beidougen/4057d2b56ee9c0f5efba3bef4f41dcb8.jpeg]

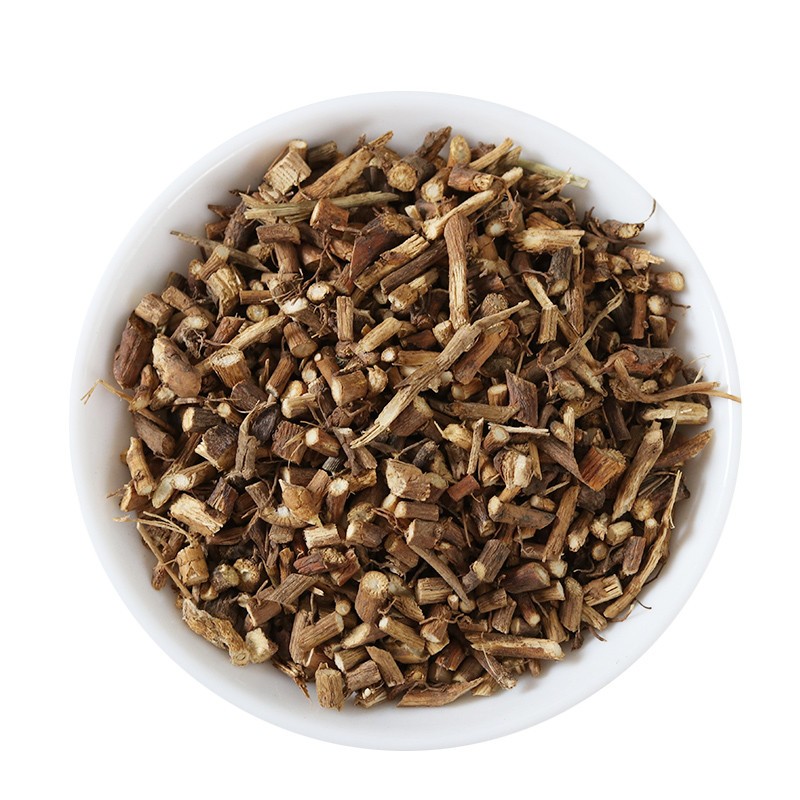

Supplement: S6 File — Numerical data underlying all figures and reported metrics, including complete training logs, evaluation results, per-class performance values, and confusion matrices. (ZIP) [file pone.0344262.s006.zip › Dataset/sample_images/beidougen/4cbd2e2abdbec4f06fab1f7b5a541a51.jpg]

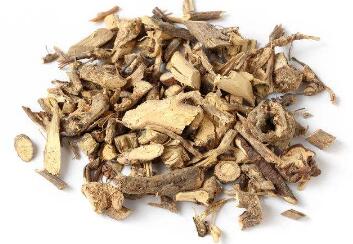

Supplement: S6 File — Numerical data underlying all figures and reported metrics, including complete training logs, evaluation results, per-class performance values, and confusion matrices. (ZIP) [file pone.0344262.s006.zip › Dataset/sample_images/beidougen/5d772dbe9d81612f862979da90f11b5e.jpg]

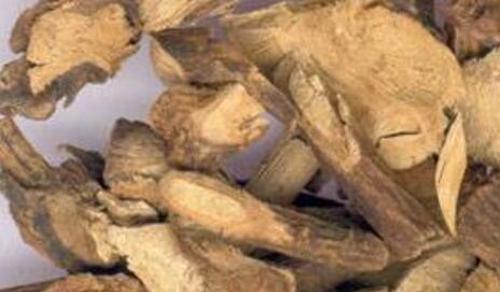

Supplement: S6 File — Numerical data underlying all figures and reported metrics, including complete training logs, evaluation results, per-class performance values, and confusion matrices. (ZIP) [file pone.0344262.s006.zip › Dataset/sample_images/beidougen/5f8c8ecd6dc517981ad8b3ba548ede02.jpg]

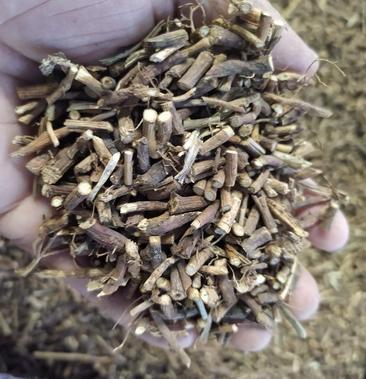

Supplement: S6 File — Numerical data underlying all figures and reported metrics, including complete training logs, evaluation results, per-class performance values, and confusion matrices. (ZIP) [file pone.0344262.s006.zip › Dataset/sample_images/beidougen/82b057e54f5c5435af0af69b9728d8fa.jpeg]

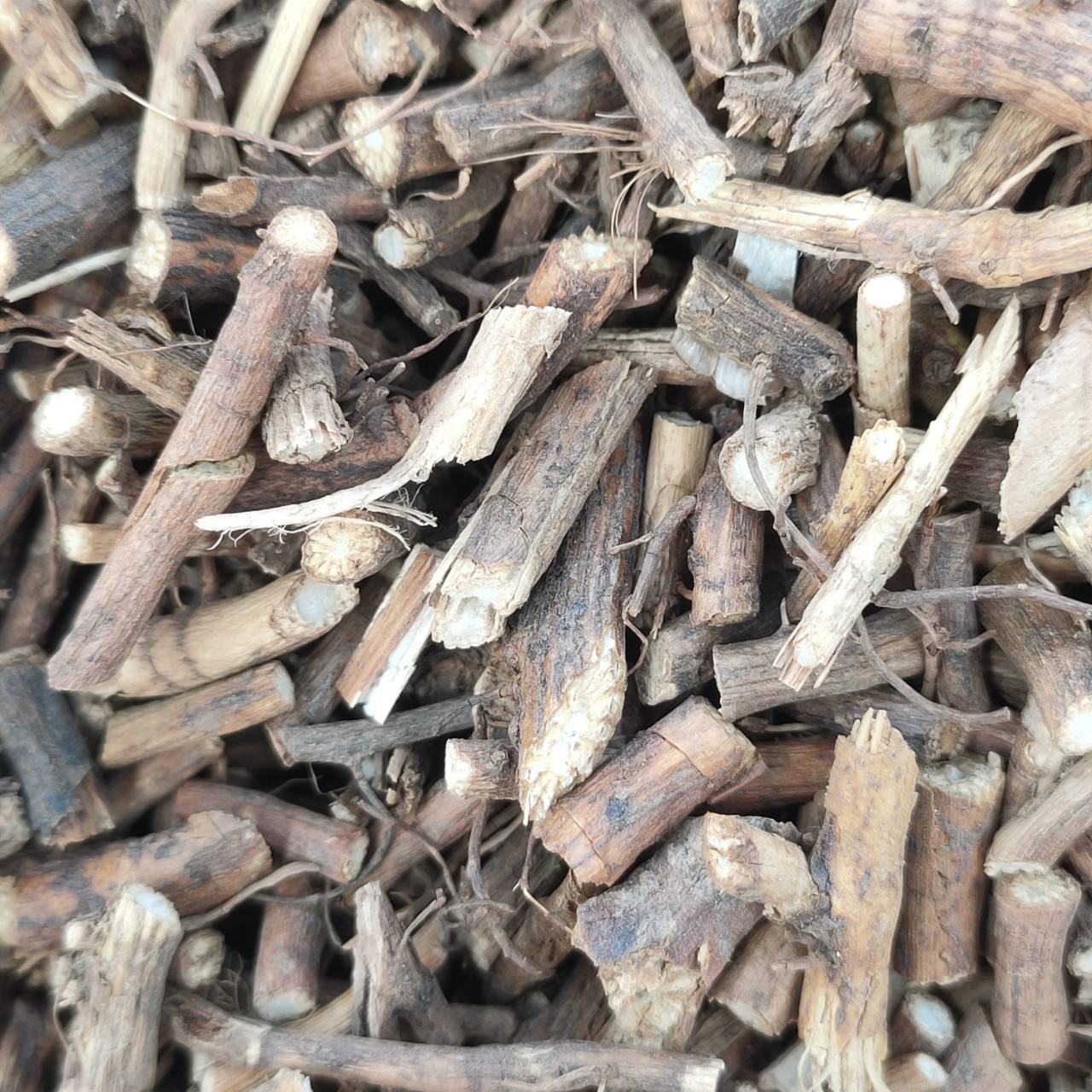

Supplement: S6 File — Numerical data underlying all figures and reported metrics, including complete training logs, evaluation results, per-class performance values, and confusion matrices. (ZIP) [file pone.0344262.s006.zip › Dataset/sample_images/beidougen/8eded7c3c5df7058c34053e99d827ae9.jpeg]

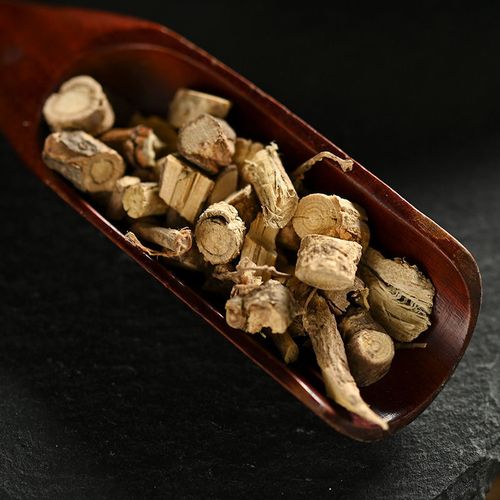

Supplement: S6 File — Numerical data underlying all figures and reported metrics, including complete training logs, evaluation results, per-class performance values, and confusion matrices. (ZIP) [file pone.0344262.s006.zip › Dataset/sample_images/beidougen/91ad12ce558751797fd137979cf7bcc4.jpeg]

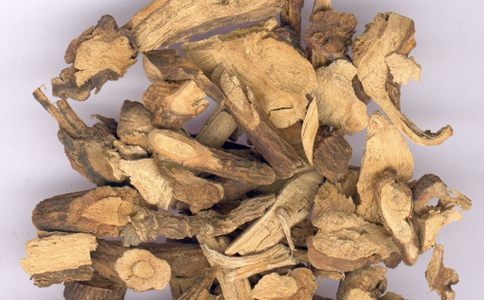

Supplement: S6 File — Numerical data underlying all figures and reported metrics, including complete training logs, evaluation results, per-class performance values, and confusion matrices. (ZIP) [file pone.0344262.s006.zip › Dataset/sample_images/beidougen/9bd5eb8c33739a0ae5e33667a874b85b.jpg]

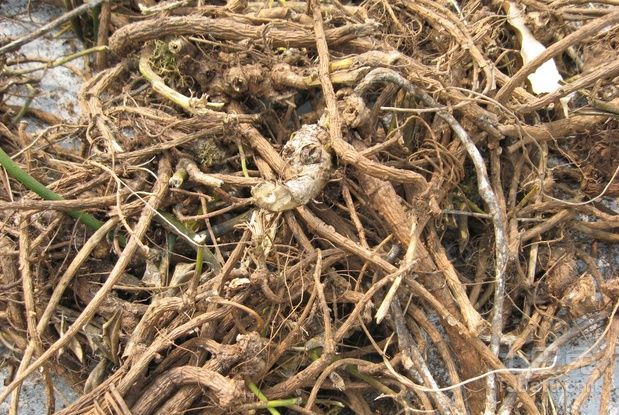

Supplement: S6 File — Numerical data underlying all figures and reported metrics, including complete training logs, evaluation results, per-class performance values, and confusion matrices. (ZIP) [file pone.0344262.s006.zip › Dataset/sample_images/beidougen/b0b27ae3c898bcbf3d2653cd5acd527d.jpg]
