# Supplementary figures and images for: Toxic chinese herbal medicine recognition in real-world images via multi-scale and attention-enhanced EfficientNetV2 (part 2 of 2)
Source: PLoS One. 2026 Mar 19;21(3):e0344262. doi: 10.1371/journal.pone.0344262 (PMC13002182; doi:10.1371/journal.pone.0344262)

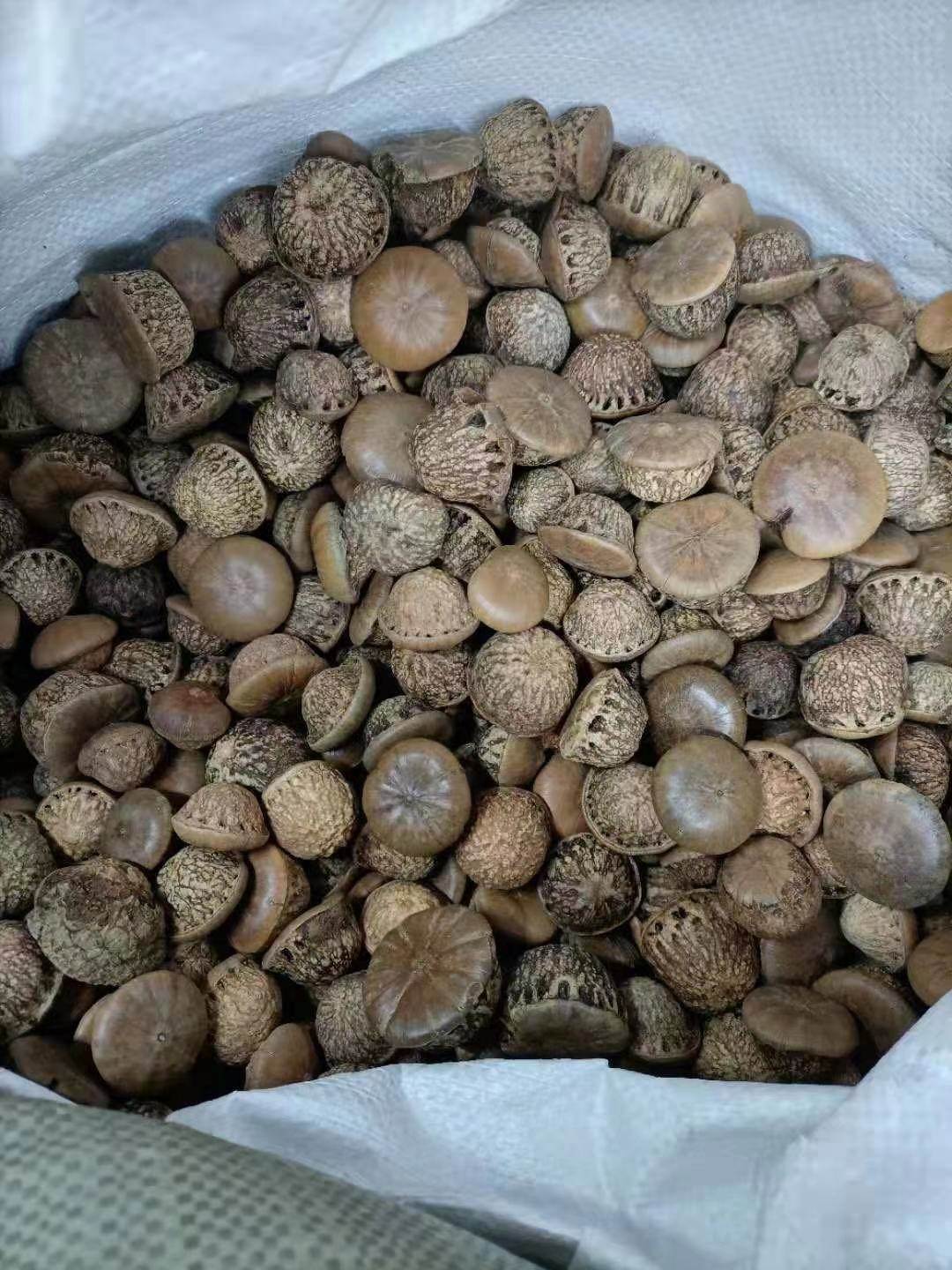

Supplement: S6 File — Numerical data underlying all figures and reported metrics, including complete training logs, evaluation results, per-class performance values, and confusion matrices. (ZIP) [file pone.0344262.s006.zip › Dataset/sample_images/beidougen/bb92c69264601abbe52ca93e1eb1b2db.jpg]

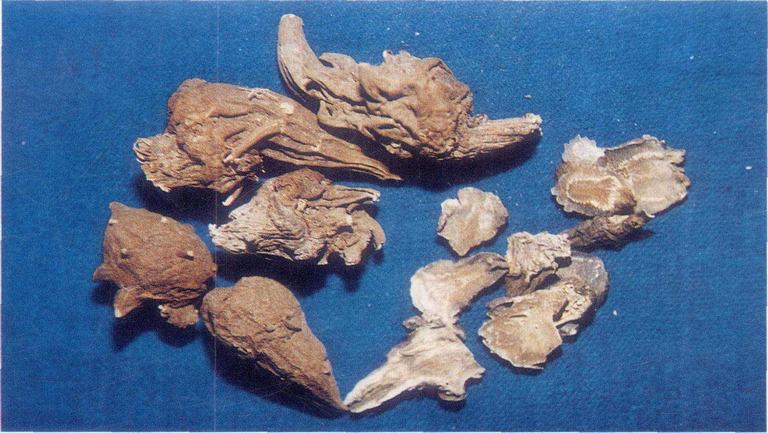

Supplement: S6 File — Numerical data underlying all figures and reported metrics, including complete training logs, evaluation results, per-class performance values, and confusion matrices. (ZIP) [file pone.0344262.s006.zip › Dataset/sample_images/caowu/28cdd0cddae04011b05c7c89a08f0042.jpeg]

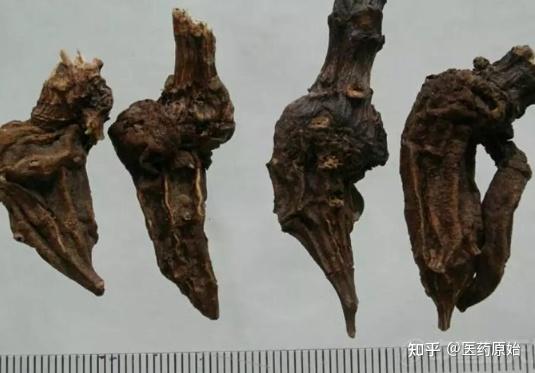

Supplement: S6 File — Numerical data underlying all figures and reported metrics, including complete training logs, evaluation results, per-class performance values, and confusion matrices. (ZIP) [file pone.0344262.s006.zip › Dataset/sample_images/caowu/30.jpg]

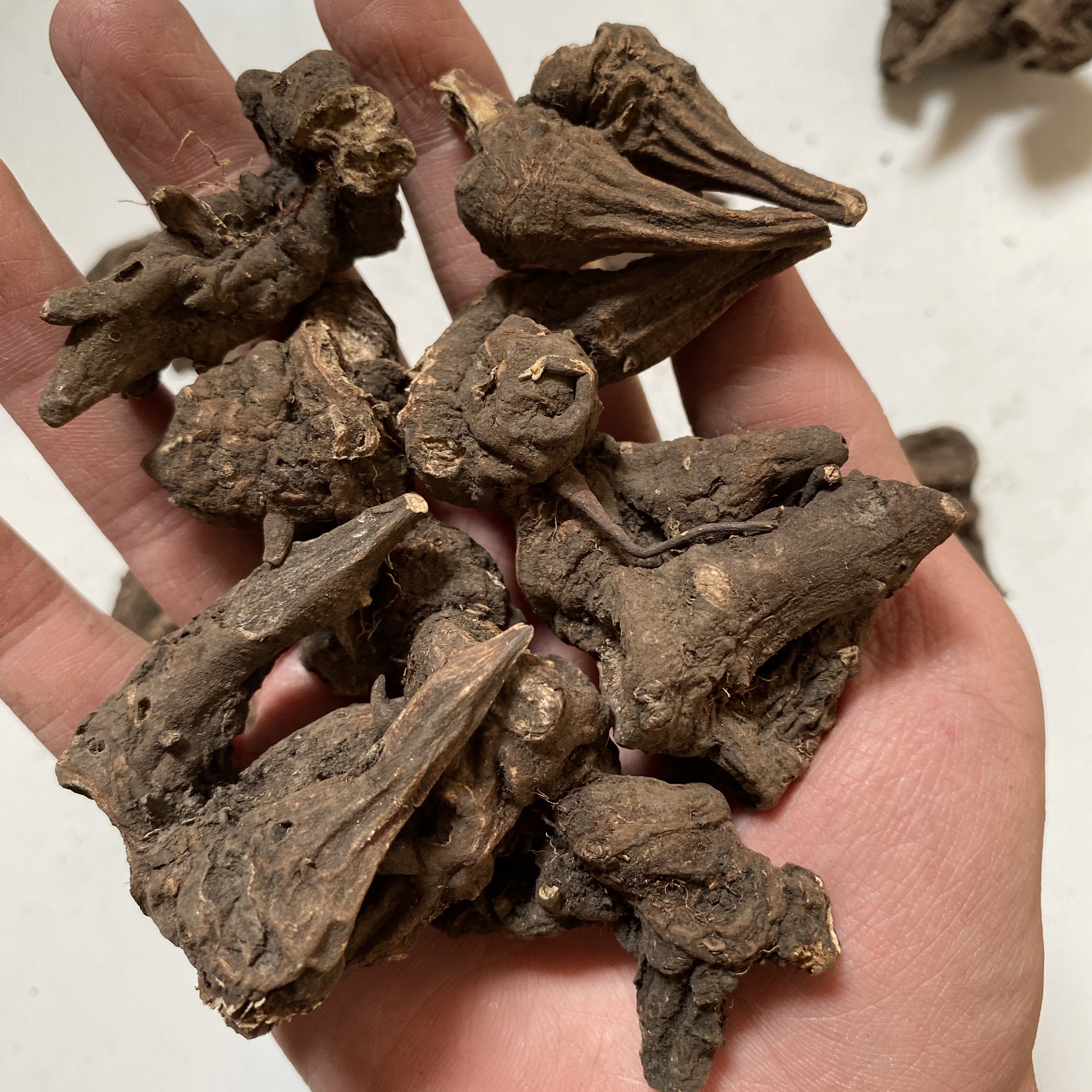

Supplement: S6 File — Numerical data underlying all figures and reported metrics, including complete training logs, evaluation results, per-class performance values, and confusion matrices. (ZIP) [file pone.0344262.s006.zip › Dataset/sample_images/caowu/3963eab20e3f1328857456549e01e634.jpg]

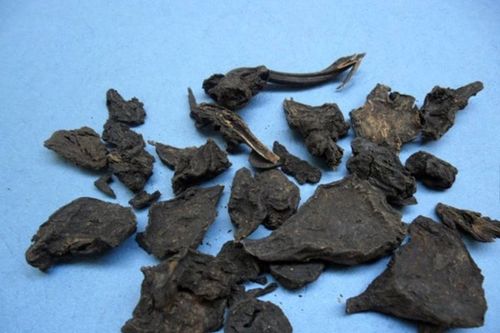

Supplement: S6 File — Numerical data underlying all figures and reported metrics, including complete training logs, evaluation results, per-class performance values, and confusion matrices. (ZIP) [file pone.0344262.s006.zip › Dataset/sample_images/caowu/54b02f3a44fa68db5f43194815a9c77e.jpeg]

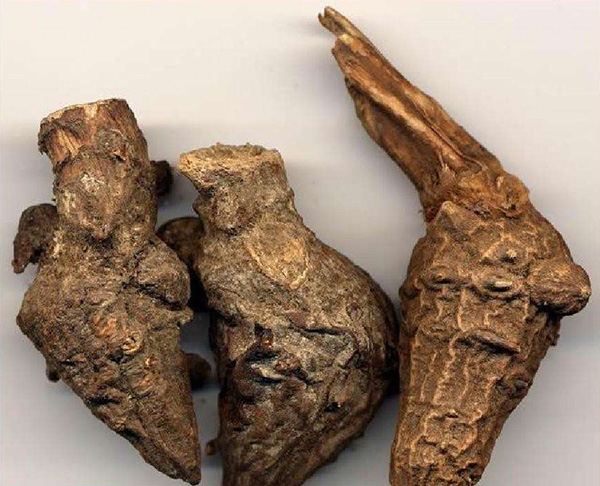

Supplement: S6 File — Numerical data underlying all figures and reported metrics, including complete training logs, evaluation results, per-class performance values, and confusion matrices. (ZIP) [file pone.0344262.s006.zip › Dataset/sample_images/caowu/557314684c02ba75f76d86c37156e4e7.jpg]

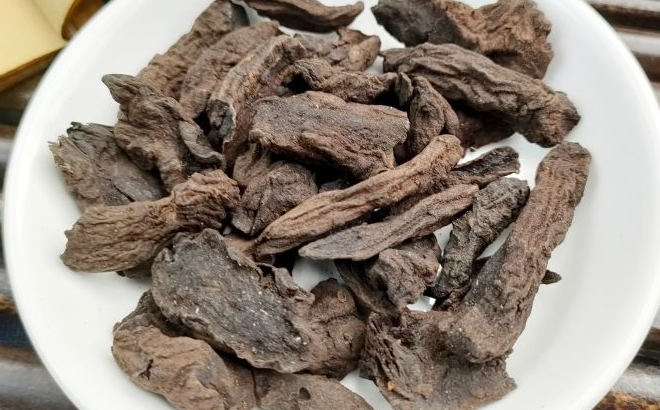

Supplement: S6 File — Numerical data underlying all figures and reported metrics, including complete training logs, evaluation results, per-class performance values, and confusion matrices. (ZIP) [file pone.0344262.s006.zip › Dataset/sample_images/caowu/591d1c41484927693a68e9306cf112f9.jpg]

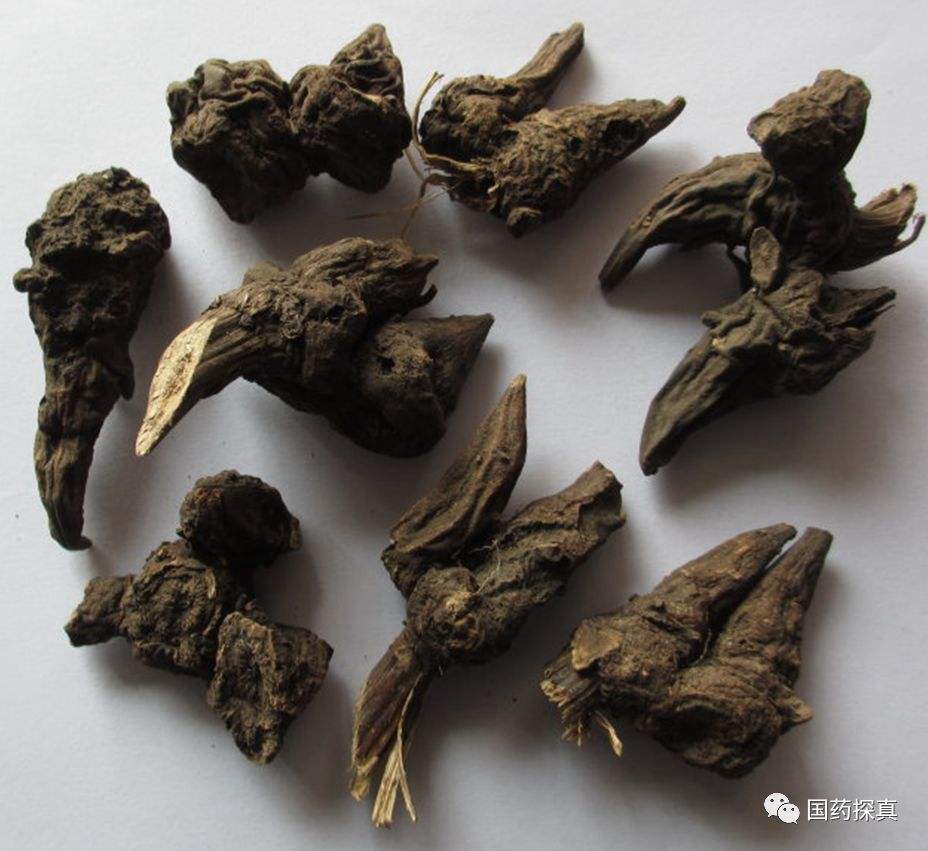

Supplement: S6 File — Numerical data underlying all figures and reported metrics, including complete training logs, evaluation results, per-class performance values, and confusion matrices. (ZIP) [file pone.0344262.s006.zip › Dataset/sample_images/caowu/6370de618ad66a3ada5bace896898328.jpeg]

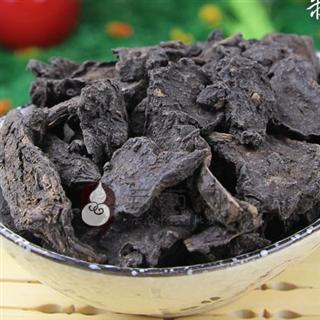

Supplement: S6 File — Numerical data underlying all figures and reported metrics, including complete training logs, evaluation results, per-class performance values, and confusion matrices. (ZIP) [file pone.0344262.s006.zip › Dataset/sample_images/caowu/6df7fc146123b9943a5e6c5f529bd7a4.jpeg]

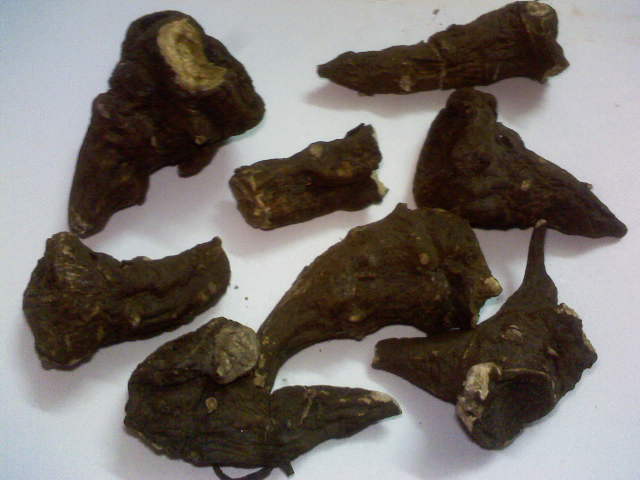

Supplement: S6 File — Numerical data underlying all figures and reported metrics, including complete training logs, evaluation results, per-class performance values, and confusion matrices. (ZIP) [file pone.0344262.s006.zip › Dataset/sample_images/caowu/7d04afe860925777d149469817cceafc.jpeg]

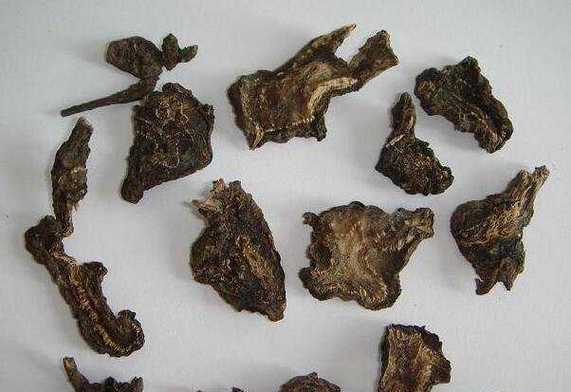

Supplement: S6 File — Numerical data underlying all figures and reported metrics, including complete training logs, evaluation results, per-class performance values, and confusion matrices. (ZIP) [file pone.0344262.s006.zip › Dataset/sample_images/caowu/b1969c23102dc25559a1af559945cb21.jpeg]

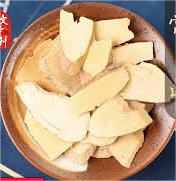

Supplement: S6 File — Numerical data underlying all figures and reported metrics, including complete training logs, evaluation results, per-class performance values, and confusion matrices. (ZIP) [file pone.0344262.s006.zip › Dataset/sample_images/changshan/10.jpg]

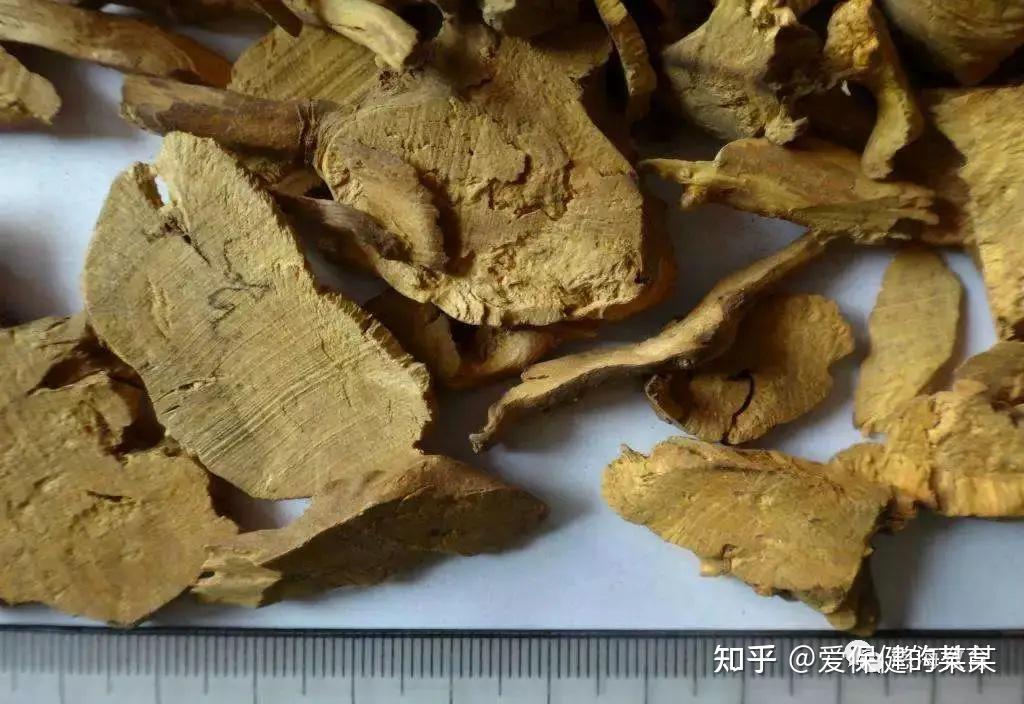

Supplement: S6 File — Numerical data underlying all figures and reported metrics, including complete training logs, evaluation results, per-class performance values, and confusion matrices. (ZIP) [file pone.0344262.s006.zip › Dataset/sample_images/changshan/13.jpg]

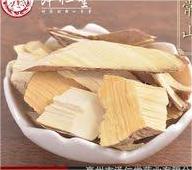

Supplement: S6 File — Numerical data underlying all figures and reported metrics, including complete training logs, evaluation results, per-class performance values, and confusion matrices. (ZIP) [file pone.0344262.s006.zip › Dataset/sample_images/changshan/19.jpg]

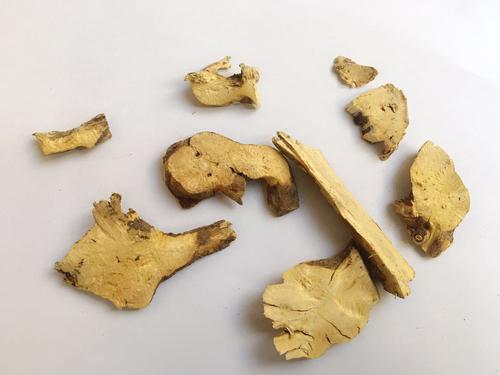

Supplement: S6 File — Numerical data underlying all figures and reported metrics, including complete training logs, evaluation results, per-class performance values, and confusion matrices. (ZIP) [file pone.0344262.s006.zip › Dataset/sample_images/changshan/2d8c6940fb7ad555e16a38278b1d12de.jpg]

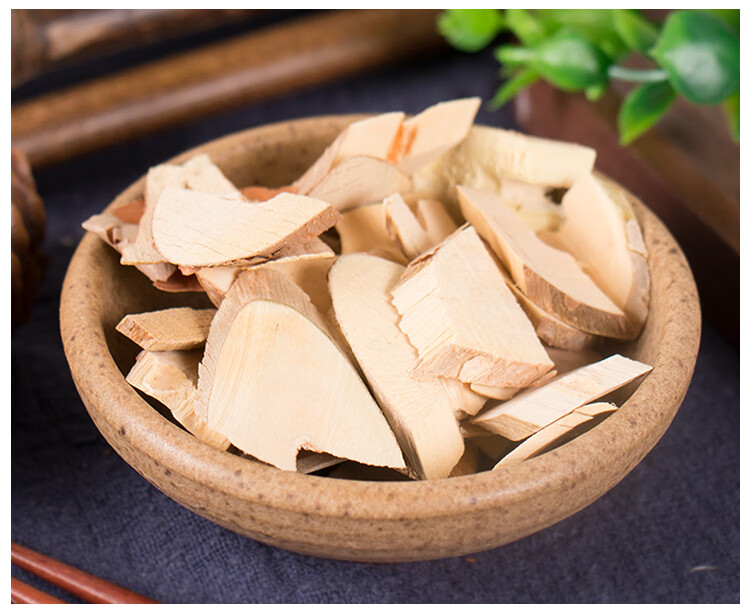

Supplement: S6 File — Numerical data underlying all figures and reported metrics, including complete training logs, evaluation results, per-class performance values, and confusion matrices. (ZIP) [file pone.0344262.s006.zip › Dataset/sample_images/changshan/461ee29b456998378d8e72f964e3f9ce.jpg]

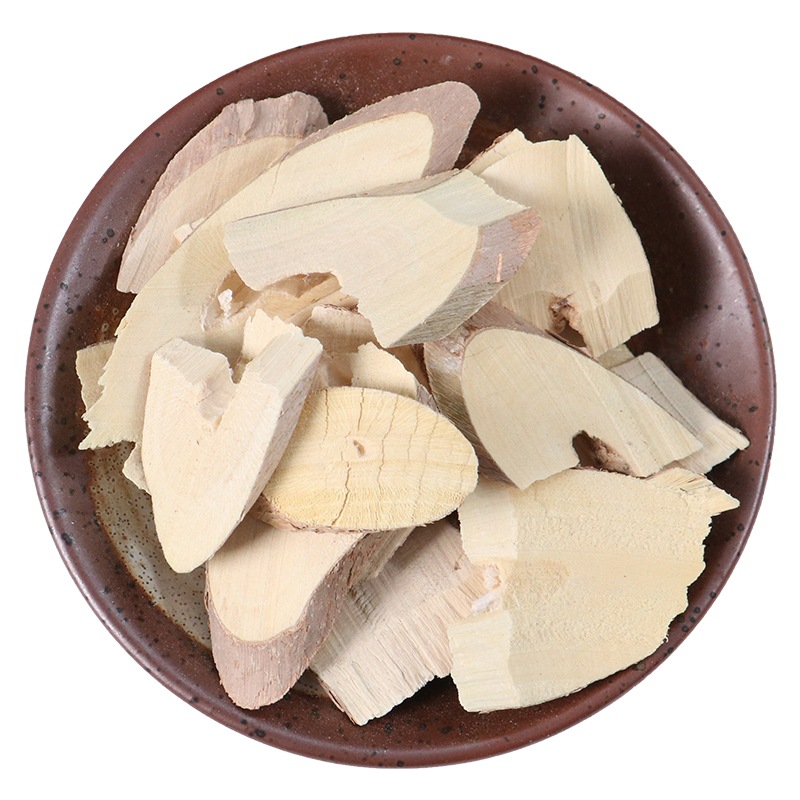

Supplement: S6 File — Numerical data underlying all figures and reported metrics, including complete training logs, evaluation results, per-class performance values, and confusion matrices. (ZIP) [file pone.0344262.s006.zip › Dataset/sample_images/changshan/64889f3a883dad20c351785fc4911e15.jpg]

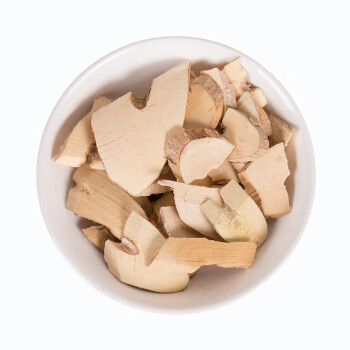

Supplement: S6 File — Numerical data underlying all figures and reported metrics, including complete training logs, evaluation results, per-class performance values, and confusion matrices. (ZIP) [file pone.0344262.s006.zip › Dataset/sample_images/changshan/698bd8b426395ad24ee300fb9c5de70a.jpg]

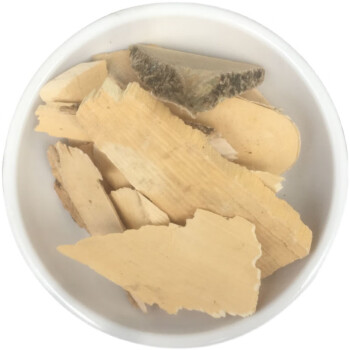

Supplement: S6 File — Numerical data underlying all figures and reported metrics, including complete training logs, evaluation results, per-class performance values, and confusion matrices. (ZIP) [file pone.0344262.s006.zip › Dataset/sample_images/changshan/7f43a82588763d590be82f3a152937db.jpg]

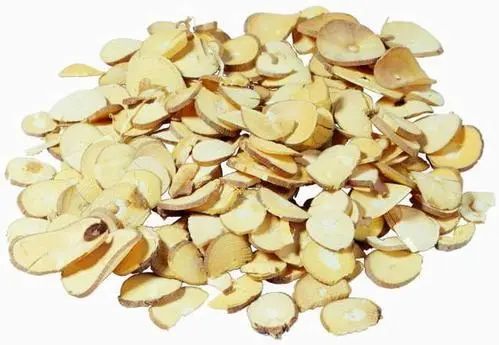

Supplement: S6 File — Numerical data underlying all figures and reported metrics, including complete training logs, evaluation results, per-class performance values, and confusion matrices. (ZIP) [file pone.0344262.s006.zip › Dataset/sample_images/changshan/b640cbfdf3202276d37569119f9f38b4.jpeg]

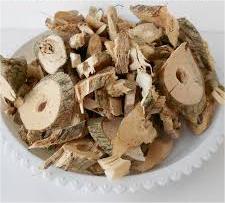

Supplement: S6 File — Numerical data underlying all figures and reported metrics, including complete training logs, evaluation results, per-class performance values, and confusion matrices. (ZIP) [file pone.0344262.s006.zip › Dataset/sample_images/changshan/images.jpg]

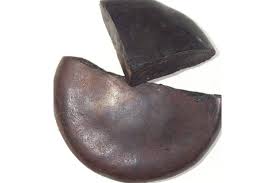

Supplement: S6 File — Numerical data underlying all figures and reported metrics, including complete training logs, evaluation results, per-class performance values, and confusion matrices. (ZIP) [file pone.0344262.s006.zip › Dataset/sample_images/chansu/1.jpg]

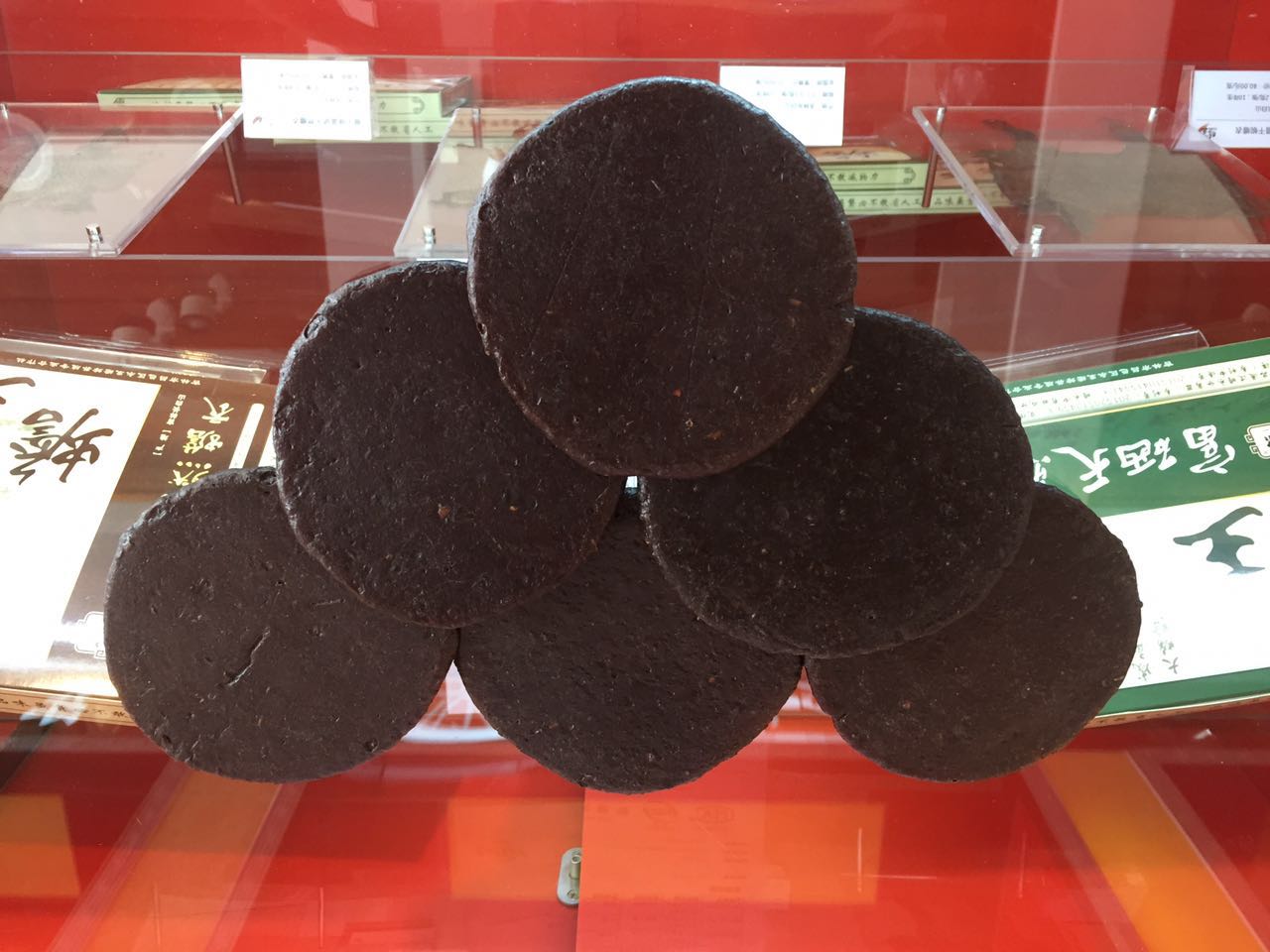

Supplement: S6 File — Numerical data underlying all figures and reported metrics, including complete training logs, evaluation results, per-class performance values, and confusion matrices. (ZIP) [file pone.0344262.s006.zip › Dataset/sample_images/chansu/12ec75c80cc96ef216dfff2ea12e44a9.jpeg]

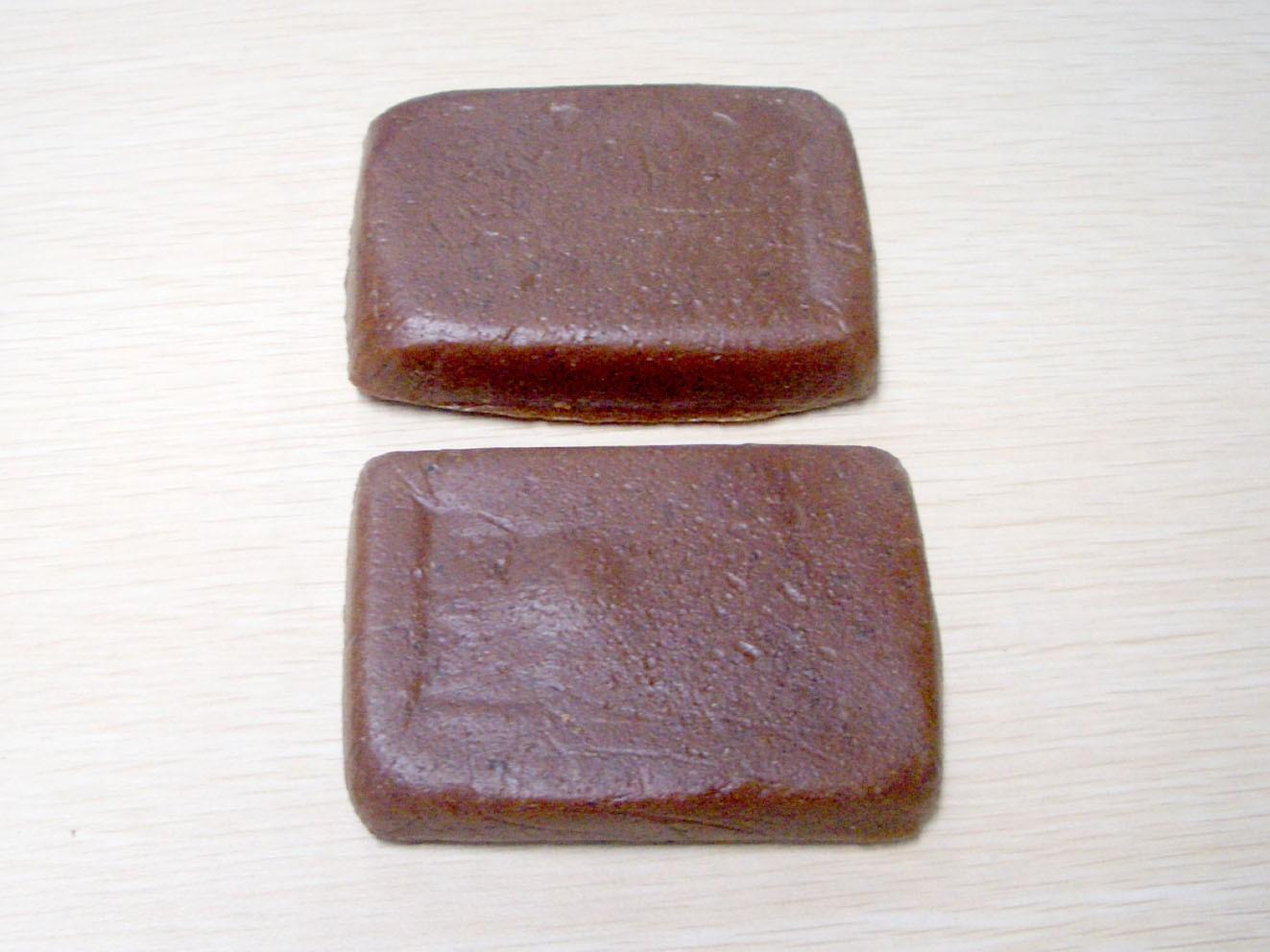

Supplement: S6 File — Numerical data underlying all figures and reported metrics, including complete training logs, evaluation results, per-class performance values, and confusion matrices. (ZIP) [file pone.0344262.s006.zip › Dataset/sample_images/chansu/18ee7fa643f65ad21155e1d17789c6c4.jpg]

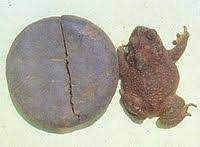

Supplement: S6 File — Numerical data underlying all figures and reported metrics, including complete training logs, evaluation results, per-class performance values, and confusion matrices. (ZIP) [file pone.0344262.s006.zip › Dataset/sample_images/chansu/2.jpg]

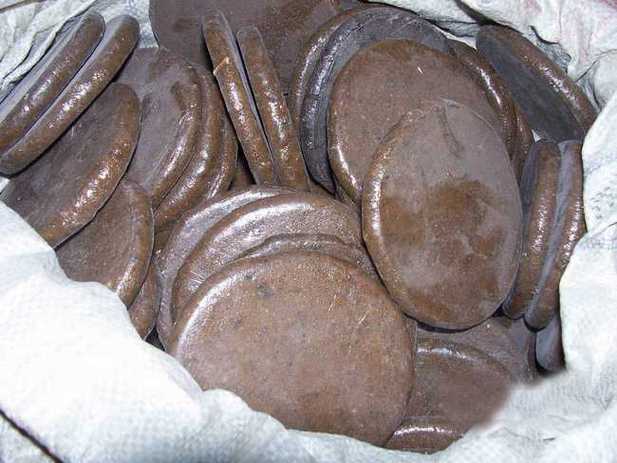

Supplement: S6 File — Numerical data underlying all figures and reported metrics, including complete training logs, evaluation results, per-class performance values, and confusion matrices. (ZIP) [file pone.0344262.s006.zip › Dataset/sample_images/chansu/3564ffb8ddb1c6830cd18abb6727f079.jpeg]

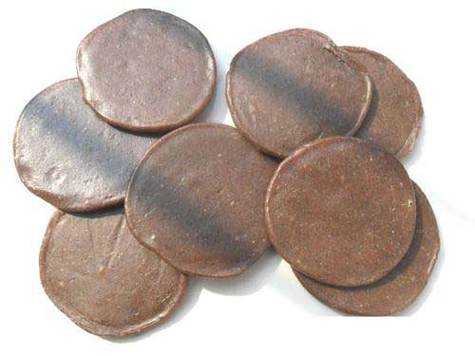

Supplement: S6 File — Numerical data underlying all figures and reported metrics, including complete training logs, evaluation results, per-class performance values, and confusion matrices. (ZIP) [file pone.0344262.s006.zip › Dataset/sample_images/chansu/42542225f54879ad011ab8d422b2a2ba.jpeg]

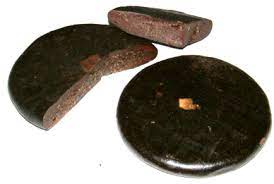

Supplement: S6 File — Numerical data underlying all figures and reported metrics, including complete training logs, evaluation results, per-class performance values, and confusion matrices. (ZIP) [file pone.0344262.s006.zip › Dataset/sample_images/chansu/5.jpg]

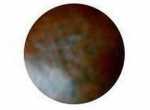

Supplement: S6 File — Numerical data underlying all figures and reported metrics, including complete training logs, evaluation results, per-class performance values, and confusion matrices. (ZIP) [file pone.0344262.s006.zip › Dataset/sample_images/chansu/628c16d03774b803545419e2c56e40c3.jpg]

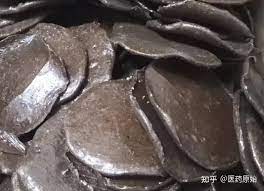

Supplement: S6 File — Numerical data underlying all figures and reported metrics, including complete training logs, evaluation results, per-class performance values, and confusion matrices. (ZIP) [file pone.0344262.s006.zip › Dataset/sample_images/chansu/8.jpg]

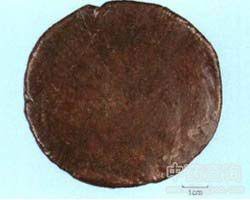

Supplement: S6 File — Numerical data underlying all figures and reported metrics, including complete training logs, evaluation results, per-class performance values, and confusion matrices. (ZIP) [file pone.0344262.s006.zip › Dataset/sample_images/chansu/9.jpg]

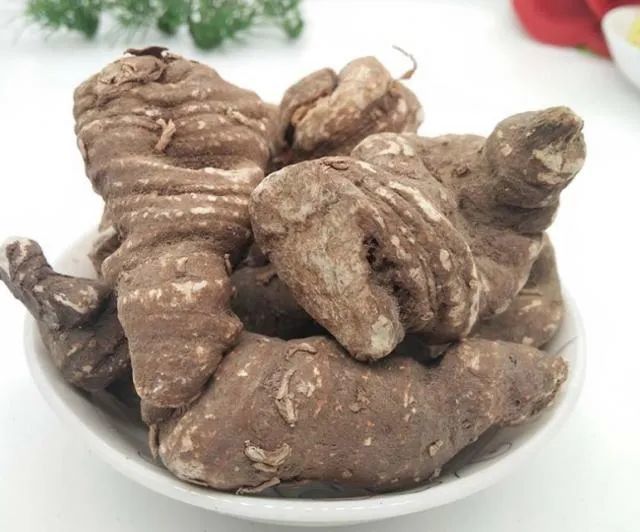

Supplement: S6 File — Numerical data underlying all figures and reported metrics, including complete training logs, evaluation results, per-class performance values, and confusion matrices. (ZIP) [file pone.0344262.s006.zip › Dataset/sample_images/chonglou/1dfc4d2f1c0305272d605414adf6d38c.jpeg]

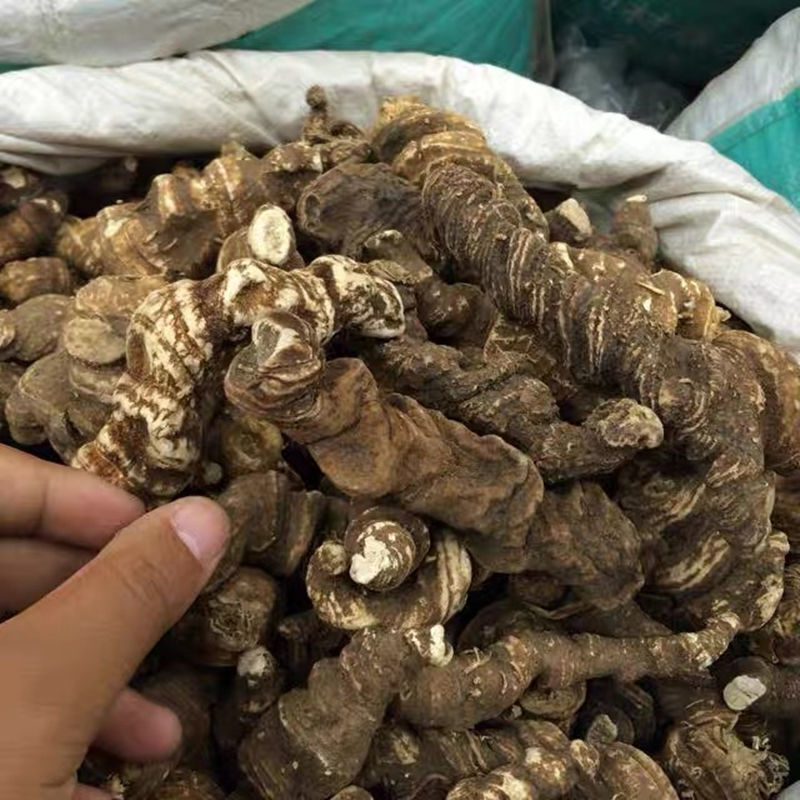

Supplement: S6 File — Numerical data underlying all figures and reported metrics, including complete training logs, evaluation results, per-class performance values, and confusion matrices. (ZIP) [file pone.0344262.s006.zip › Dataset/sample_images/chonglou/270faf7d030b689728a2e557613a01a8.jpg]

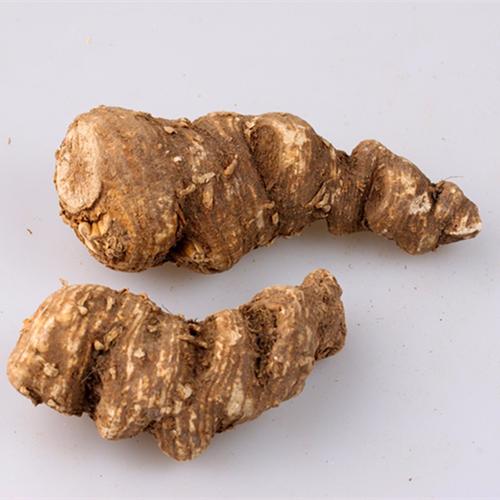

Supplement: S6 File — Numerical data underlying all figures and reported metrics, including complete training logs, evaluation results, per-class performance values, and confusion matrices. (ZIP) [file pone.0344262.s006.zip › Dataset/sample_images/chonglou/278bb9d5d1d5e6eb0cf2c654218d85e9.jpeg]

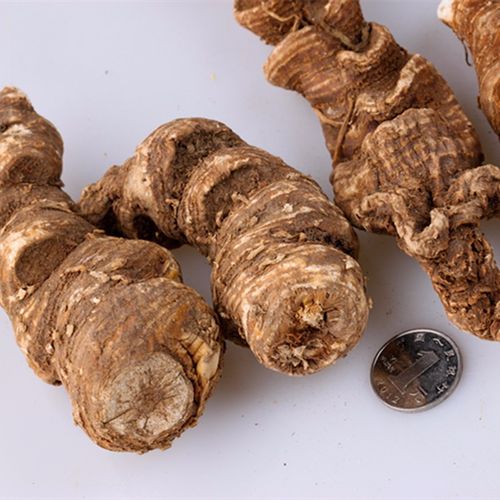

Supplement: S6 File — Numerical data underlying all figures and reported metrics, including complete training logs, evaluation results, per-class performance values, and confusion matrices. (ZIP) [file pone.0344262.s006.zip › Dataset/sample_images/chonglou/6cf2a9ae8d2061eab63dcbc186b7ccb5.jpeg]

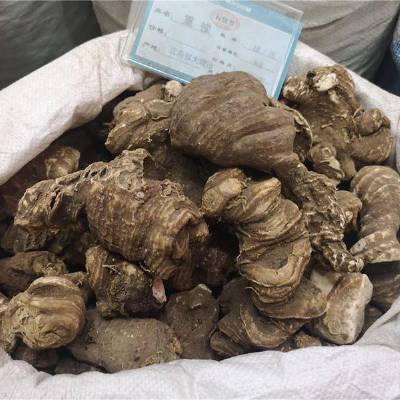

Supplement: S6 File — Numerical data underlying all figures and reported metrics, including complete training logs, evaluation results, per-class performance values, and confusion matrices. (ZIP) [file pone.0344262.s006.zip › Dataset/sample_images/chonglou/78aa67b72bac6fdfcfd8abfcd0ca7d24.jpeg]

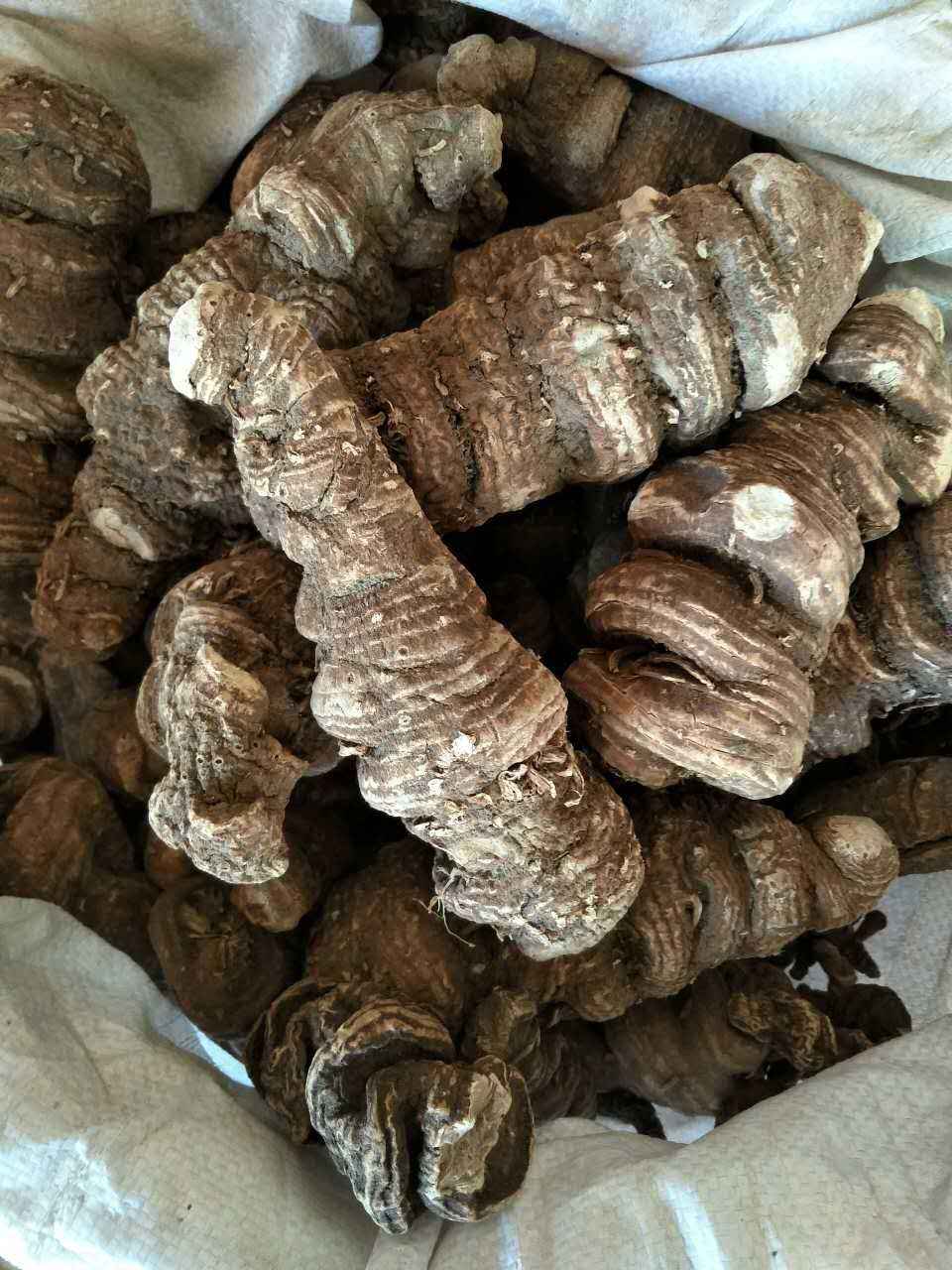

Supplement: S6 File — Numerical data underlying all figures and reported metrics, including complete training logs, evaluation results, per-class performance values, and confusion matrices. (ZIP) [file pone.0344262.s006.zip › Dataset/sample_images/chonglou/80829ee8523cad5e214b7132bde91812.jpeg]

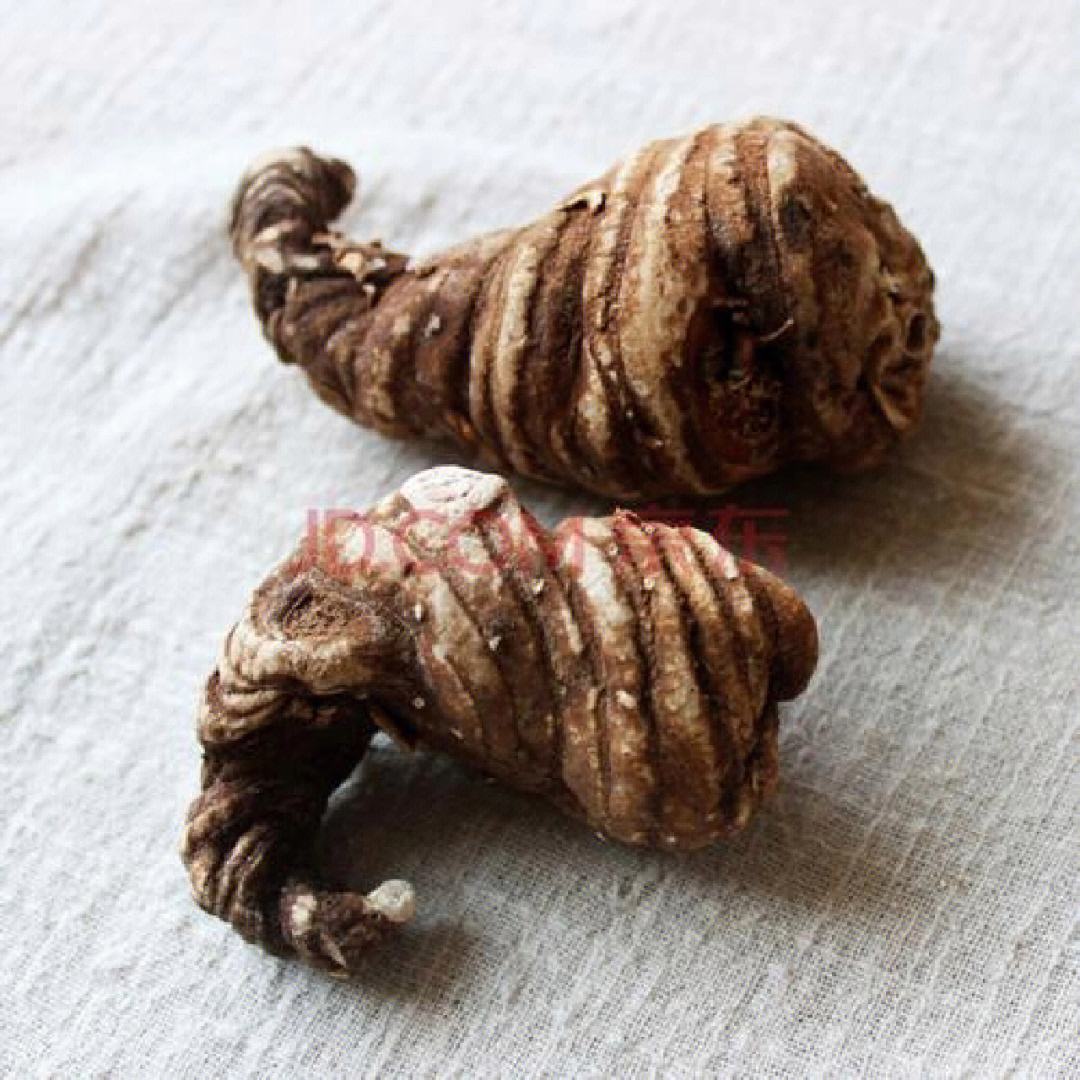

Supplement: S6 File — Numerical data underlying all figures and reported metrics, including complete training logs, evaluation results, per-class performance values, and confusion matrices. (ZIP) [file pone.0344262.s006.zip › Dataset/sample_images/chonglou/8ab27ba1020c5e13c1fad6f9023dbcae.jpeg]

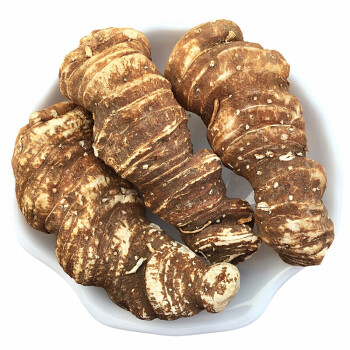

Supplement: S6 File — Numerical data underlying all figures and reported metrics, including complete training logs, evaluation results, per-class performance values, and confusion matrices. (ZIP) [file pone.0344262.s006.zip › Dataset/sample_images/chonglou/b144563444bdd4caa97ff29a4c772846.jpg]

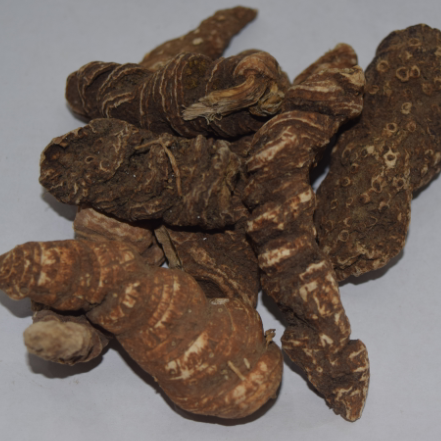

Supplement: S6 File — Numerical data underlying all figures and reported metrics, including complete training logs, evaluation results, per-class performance values, and confusion matrices. (ZIP) [file pone.0344262.s006.zip › Dataset/sample_images/chonglou/d851a7b7aa8f7bd1a105915cdd25d155.png]

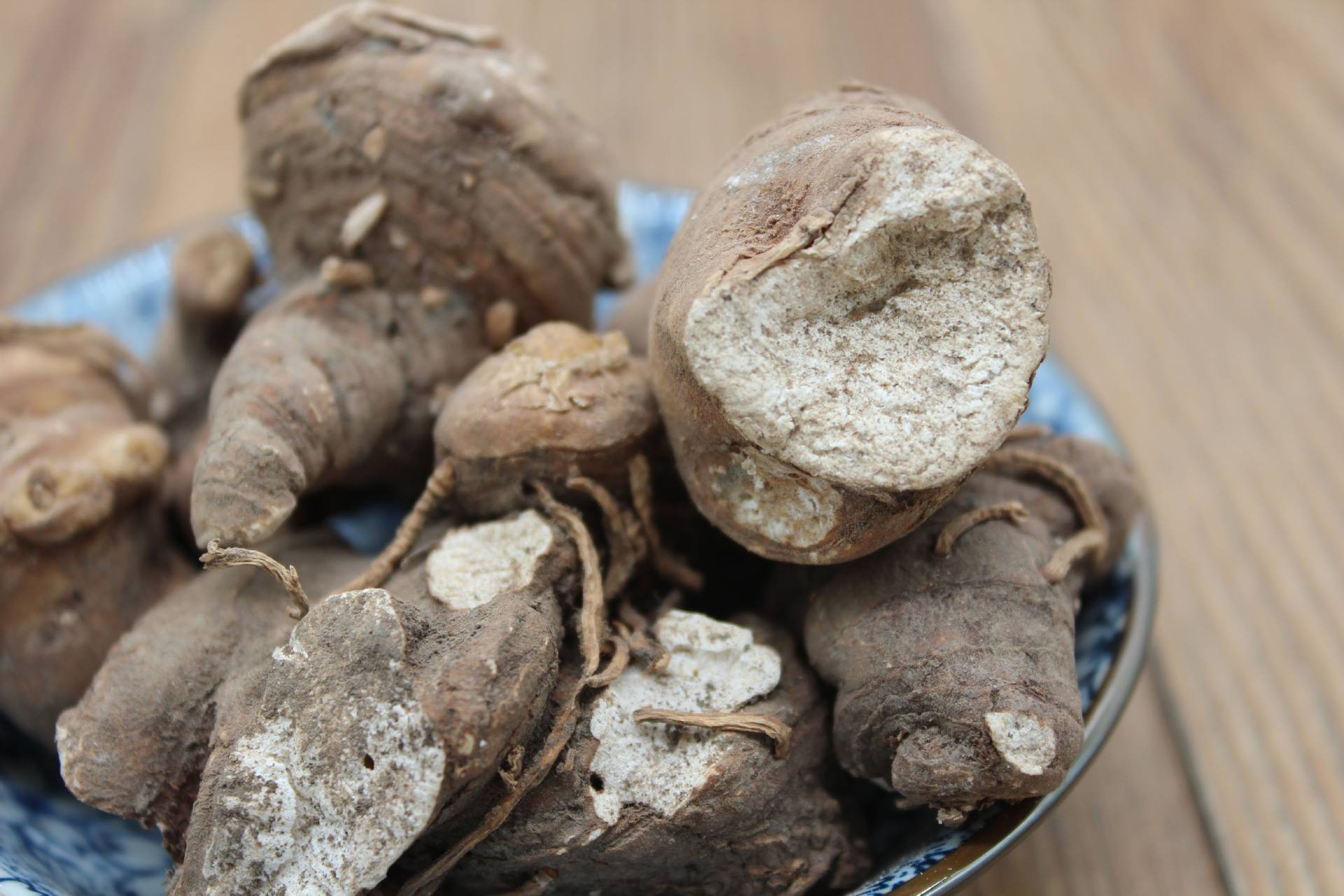

Supplement: S6 File — Numerical data underlying all figures and reported metrics, including complete training logs, evaluation results, per-class performance values, and confusion matrices. (ZIP) [file pone.0344262.s006.zip › Dataset/sample_images/chonglou/ea891dfd9fd422c89f148fc2d6042599.jpeg]

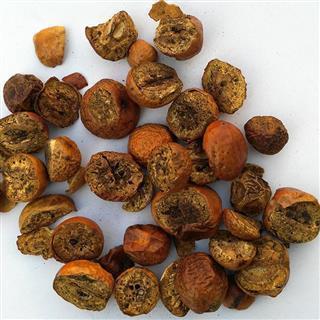

Supplement: S6 File — Numerical data underlying all figures and reported metrics, including complete training logs, evaluation results, per-class performance values, and confusion matrices. (ZIP) [file pone.0344262.s006.zip › Dataset/sample_images/chuanlianzi/0a3e34d57f059b7c226cedcf860a888d.jpeg]

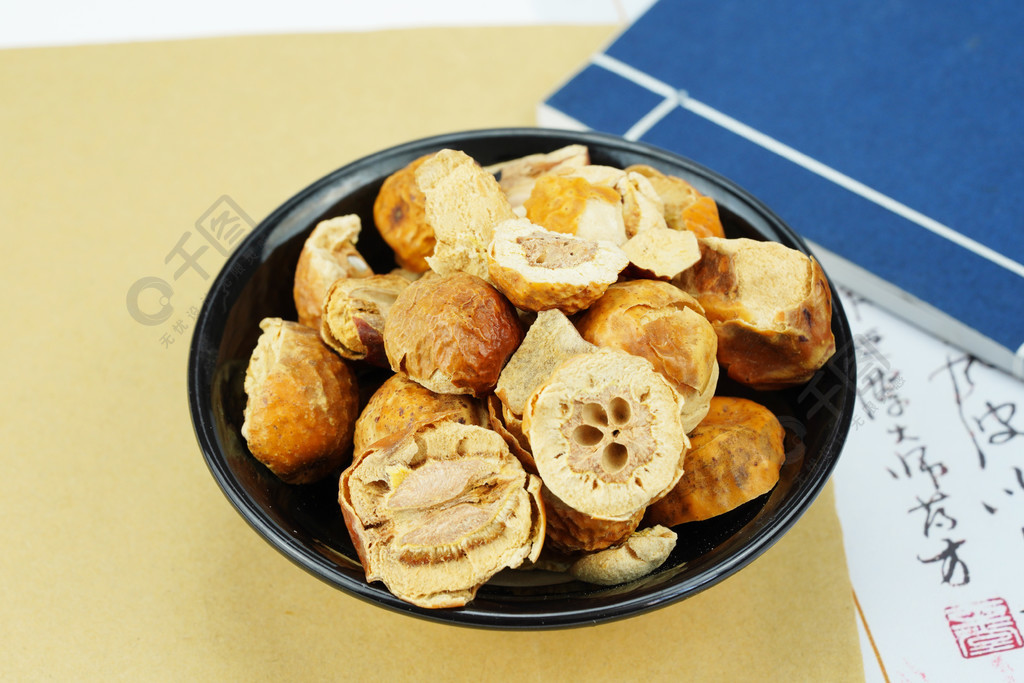

Supplement: S6 File — Numerical data underlying all figures and reported metrics, including complete training logs, evaluation results, per-class performance values, and confusion matrices. (ZIP) [file pone.0344262.s006.zip › Dataset/sample_images/chuanlianzi/344ea08e16cb804efebfcb1bf53e8847.jpeg]

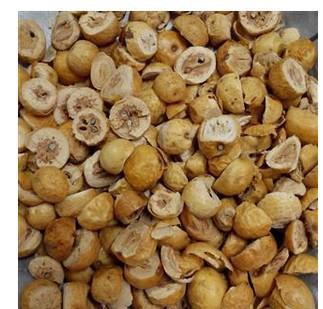

Supplement: S6 File — Numerical data underlying all figures and reported metrics, including complete training logs, evaluation results, per-class performance values, and confusion matrices. (ZIP) [file pone.0344262.s006.zip › Dataset/sample_images/chuanlianzi/3dc414e2171ccbc0f5cbdc8116dabaf3.jpeg]

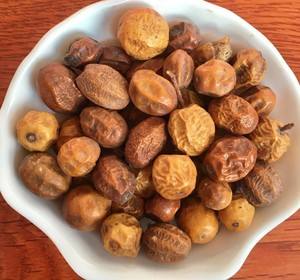

Supplement: S6 File — Numerical data underlying all figures and reported metrics, including complete training logs, evaluation results, per-class performance values, and confusion matrices. (ZIP) [file pone.0344262.s006.zip › Dataset/sample_images/chuanlianzi/53221f88cee82eafeec77f3e62ebdbbb.jpeg]

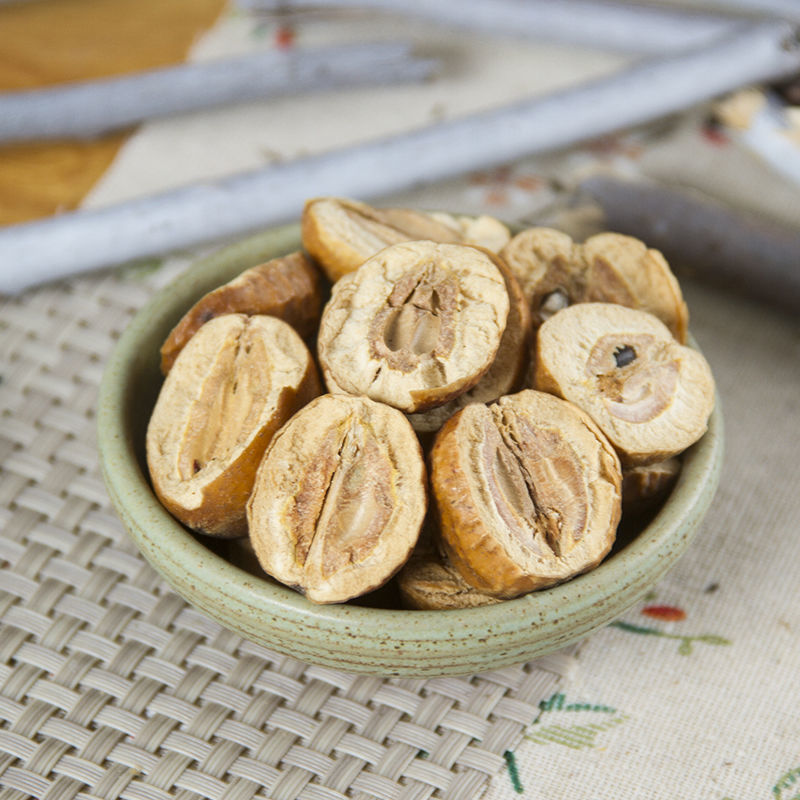

Supplement: S6 File — Numerical data underlying all figures and reported metrics, including complete training logs, evaluation results, per-class performance values, and confusion matrices. (ZIP) [file pone.0344262.s006.zip › Dataset/sample_images/chuanlianzi/5d625fb570c554829286ca3cfb6c05ec.jpg]

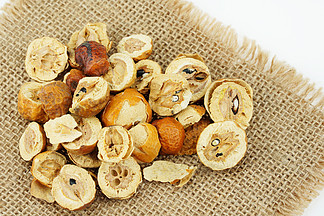

Supplement: S6 File — Numerical data underlying all figures and reported metrics, including complete training logs, evaluation results, per-class performance values, and confusion matrices. (ZIP) [file pone.0344262.s006.zip › Dataset/sample_images/chuanlianzi/6159ed051ca6cb6b1768bf7e32c3816c.jpeg]

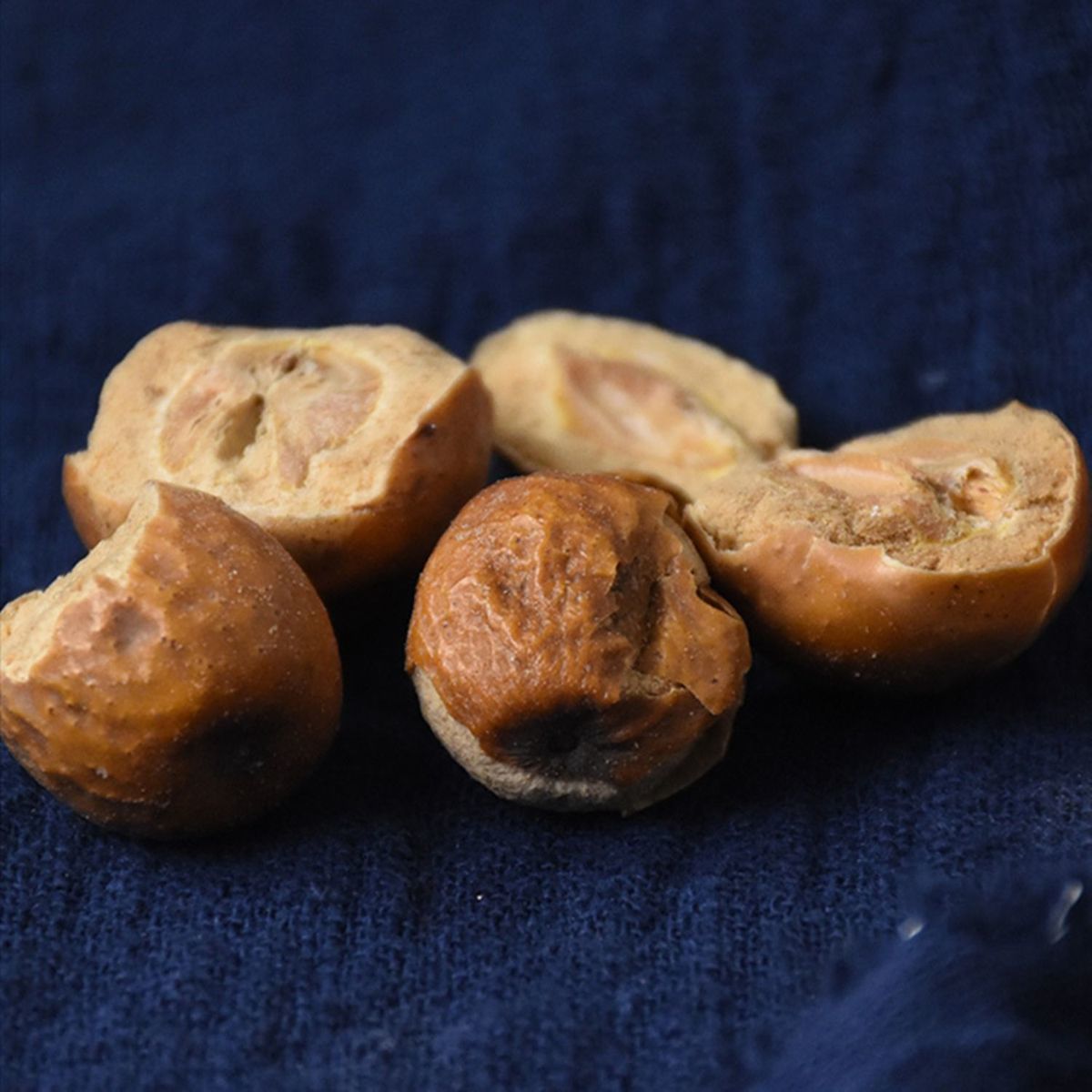

Supplement: S6 File — Numerical data underlying all figures and reported metrics, including complete training logs, evaluation results, per-class performance values, and confusion matrices. (ZIP) [file pone.0344262.s006.zip › Dataset/sample_images/chuanlianzi/67f8944292c040e6ef75c0ee4f275a7e.jpg]

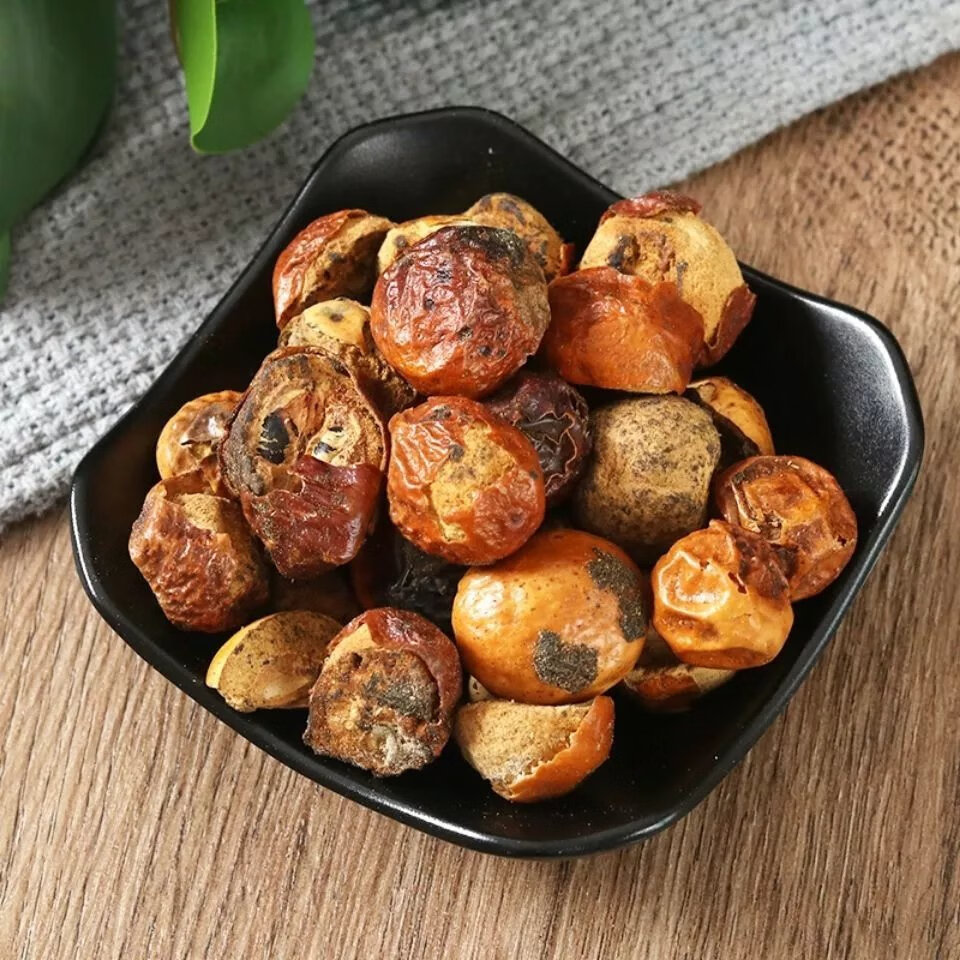

Supplement: S6 File — Numerical data underlying all figures and reported metrics, including complete training logs, evaluation results, per-class performance values, and confusion matrices. (ZIP) [file pone.0344262.s006.zip › Dataset/sample_images/chuanlianzi/8cae338a403e4de37bc85ba6efae214c.jpg]

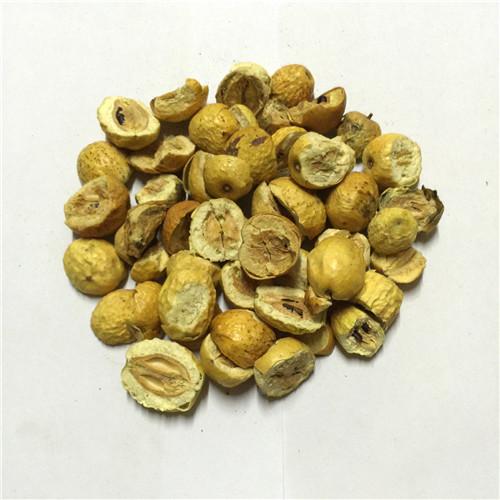

Supplement: S6 File — Numerical data underlying all figures and reported metrics, including complete training logs, evaluation results, per-class performance values, and confusion matrices. (ZIP) [file pone.0344262.s006.zip › Dataset/sample_images/chuanlianzi/91c614842e3f8098607abca31610cef7.jpeg]

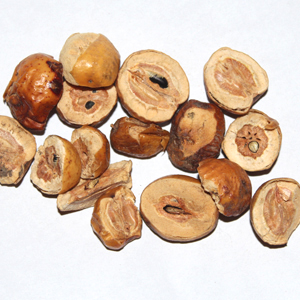

Supplement: S6 File — Numerical data underlying all figures and reported metrics, including complete training logs, evaluation results, per-class performance values, and confusion matrices. (ZIP) [file pone.0344262.s006.zip › Dataset/sample_images/chuanlianzi/9d91066a541aea2179dc0c9f2a09e5d8.jpeg]

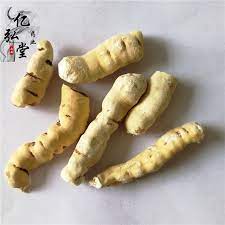

Supplement: S6 File — Numerical data underlying all figures and reported metrics, including complete training logs, evaluation results, per-class performance values, and confusion matrices. (ZIP) [file pone.0344262.s006.zip › Dataset/sample_images/gansui/11.jpg]

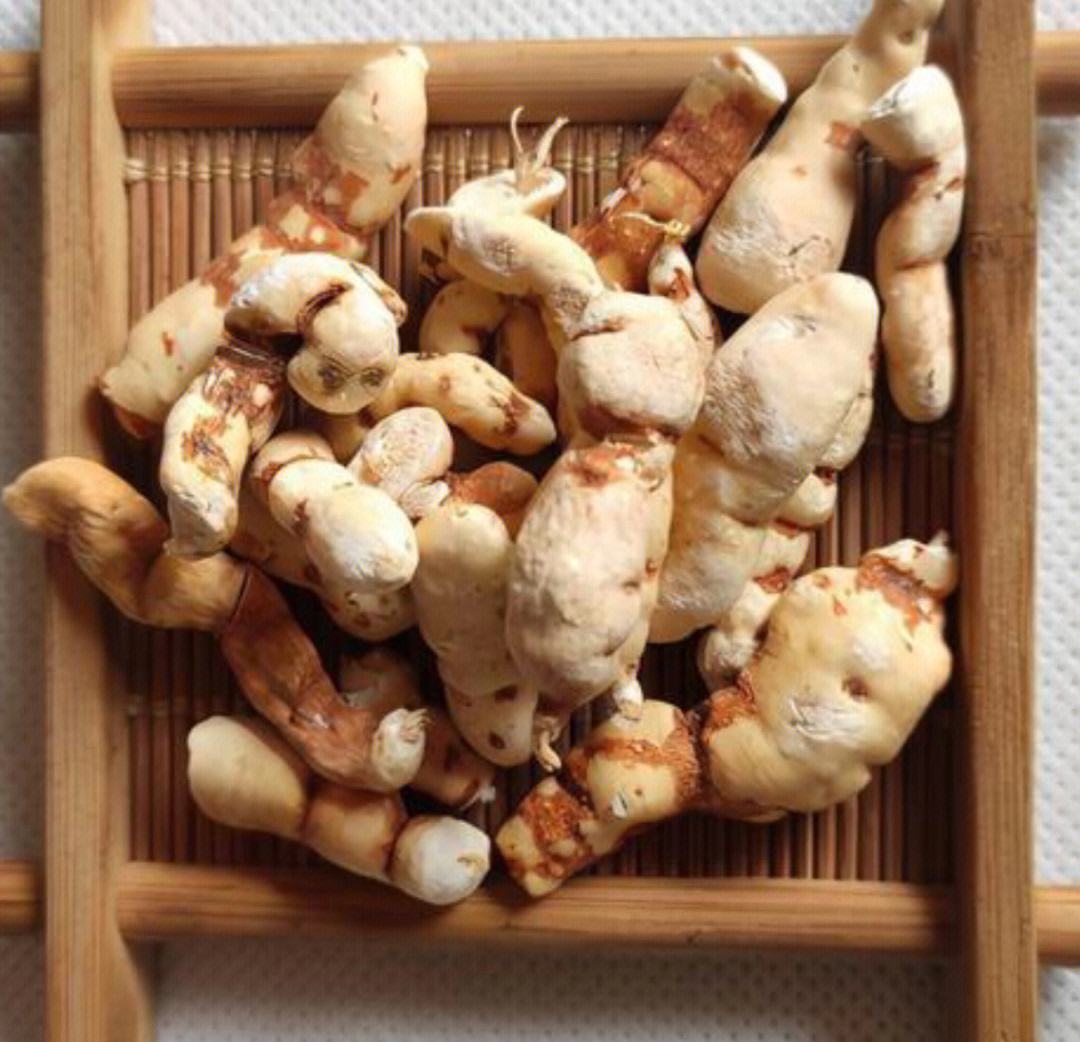

Supplement: S6 File — Numerical data underlying all figures and reported metrics, including complete training logs, evaluation results, per-class performance values, and confusion matrices. (ZIP) [file pone.0344262.s006.zip › Dataset/sample_images/gansui/17c6ef019e17f79f1095b5146d0d37a2.jpeg]

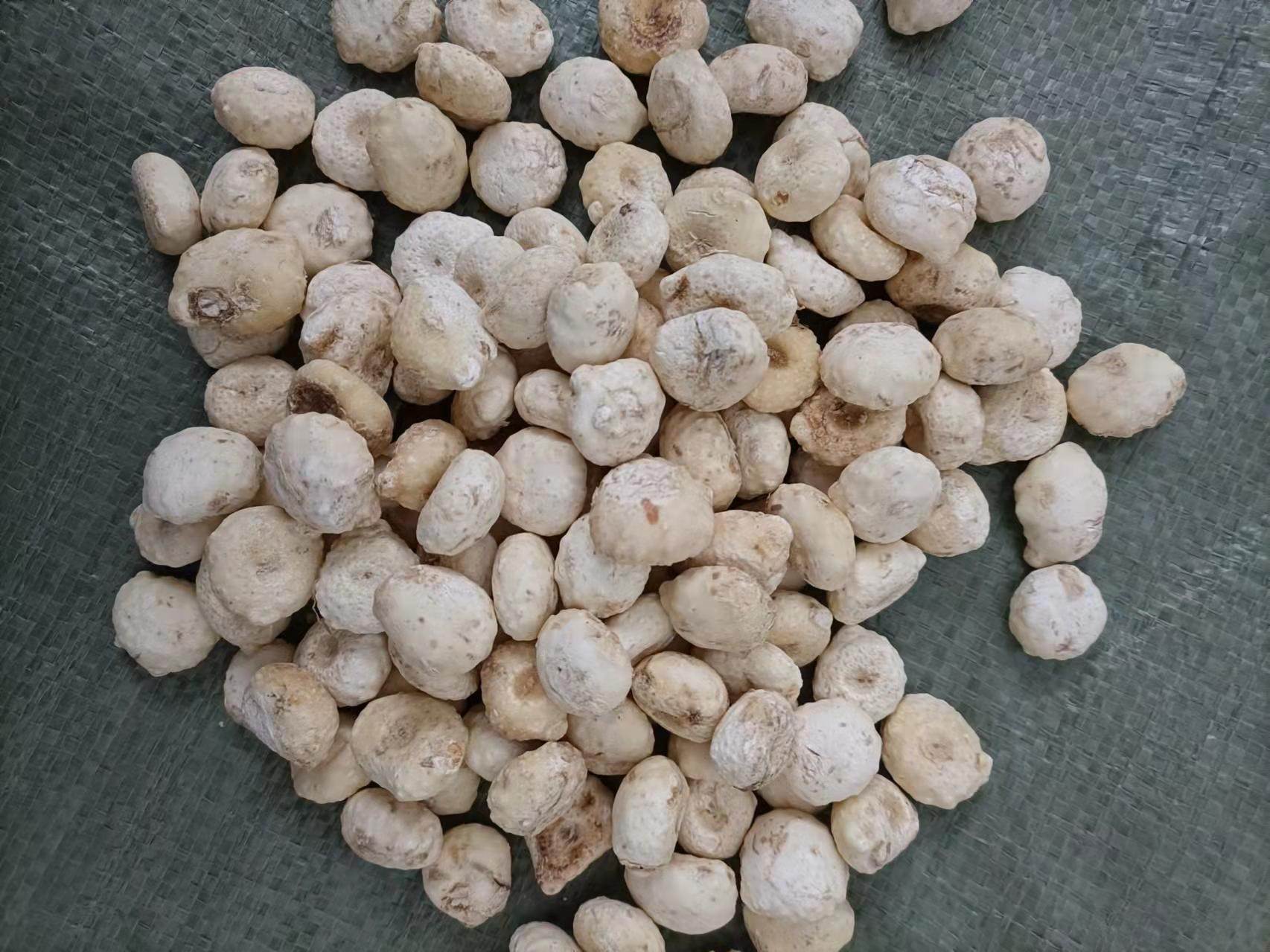

Supplement: S6 File — Numerical data underlying all figures and reported metrics, including complete training logs, evaluation results, per-class performance values, and confusion matrices. (ZIP) [file pone.0344262.s006.zip › Dataset/sample_images/gansui/28a58a9623cc47e07c92e34d08855839.jpg]

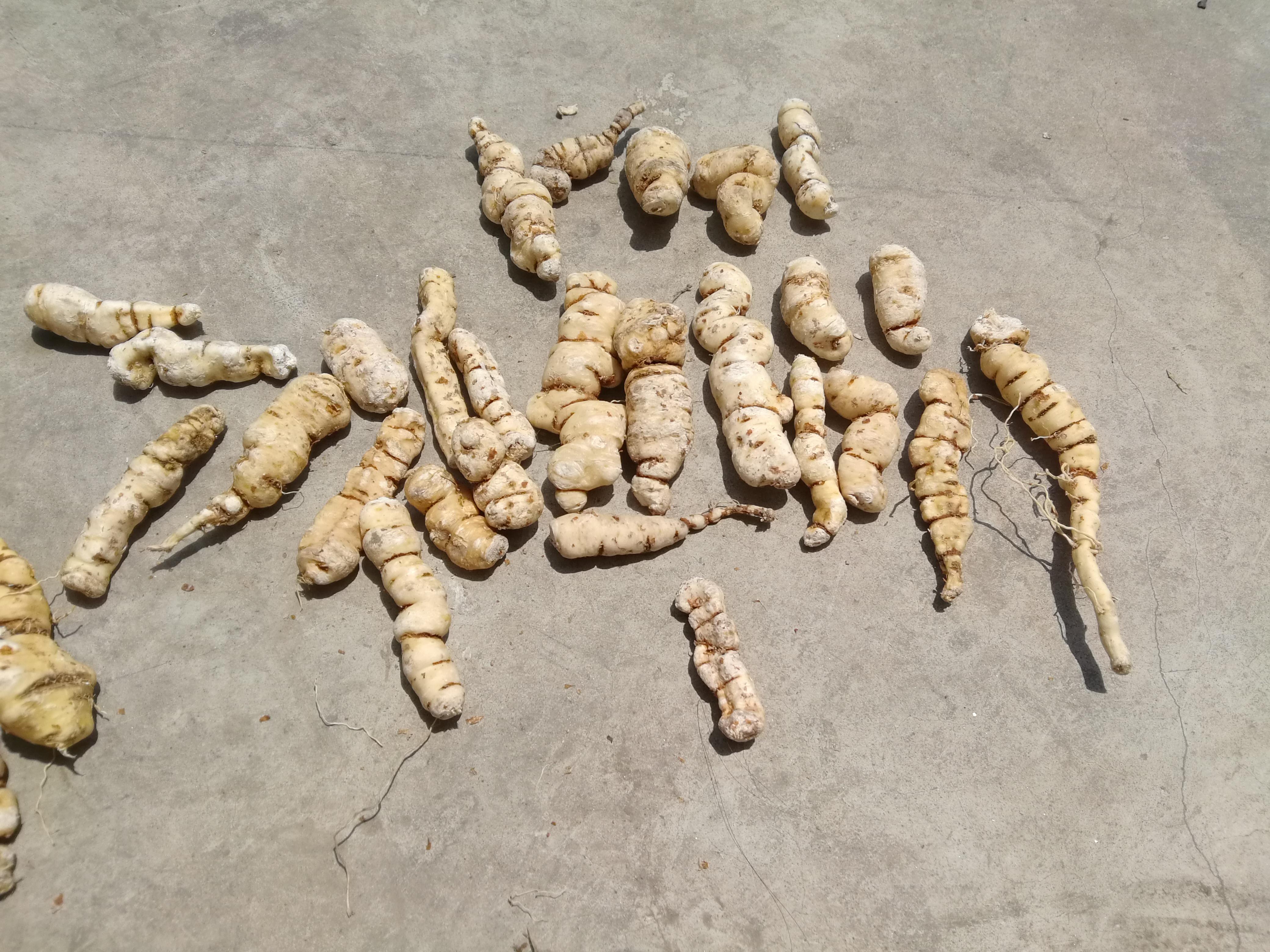

Supplement: S6 File — Numerical data underlying all figures and reported metrics, including complete training logs, evaluation results, per-class performance values, and confusion matrices. (ZIP) [file pone.0344262.s006.zip › Dataset/sample_images/gansui/44031e83591ecfae9c9a94134ec64c0f.jpg]

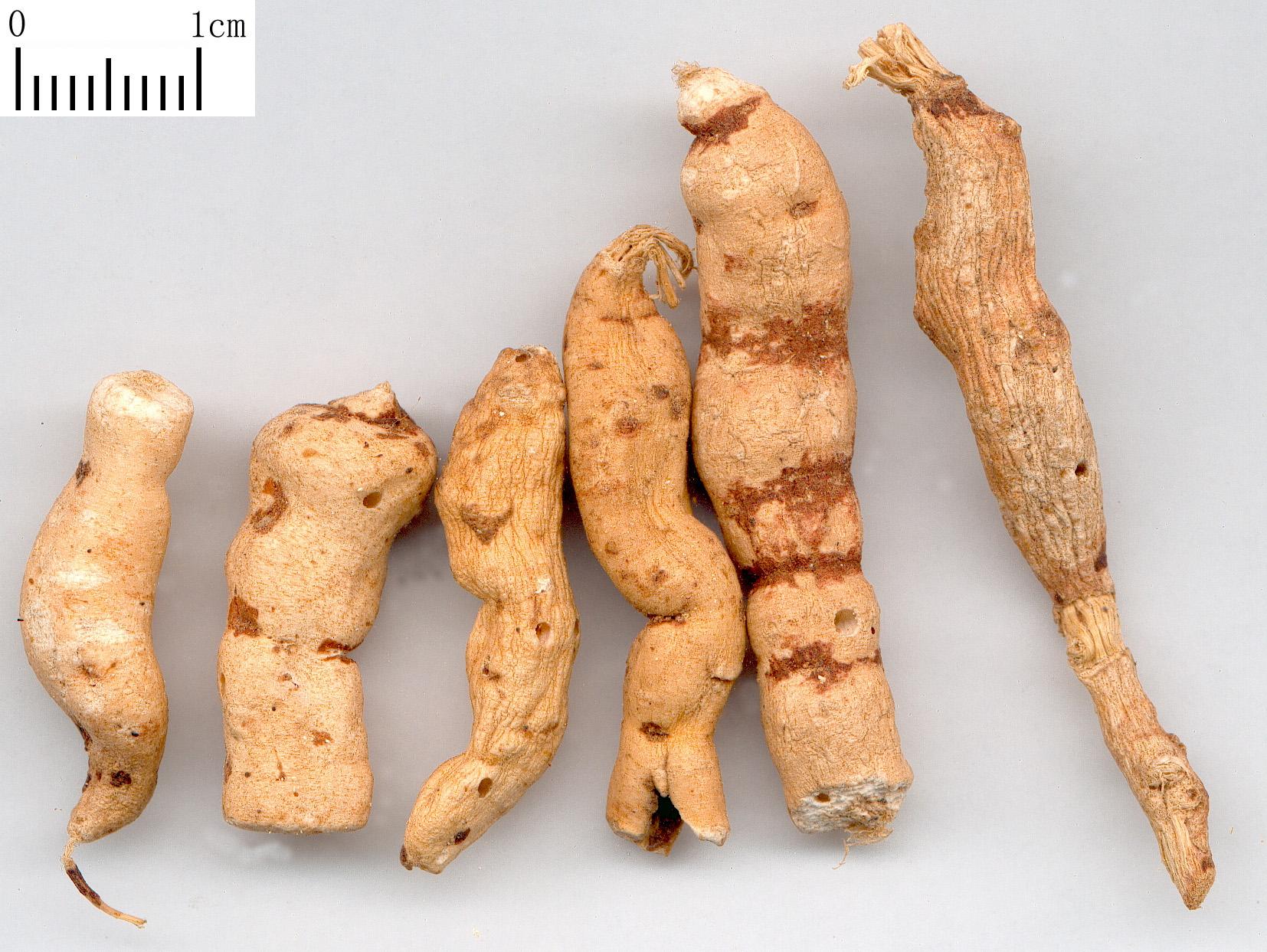

Supplement: S6 File — Numerical data underlying all figures and reported metrics, including complete training logs, evaluation results, per-class performance values, and confusion matrices. (ZIP) [file pone.0344262.s006.zip › Dataset/sample_images/gansui/9d7de752d07d75bccbff307f0ddfe4a4.jpeg]

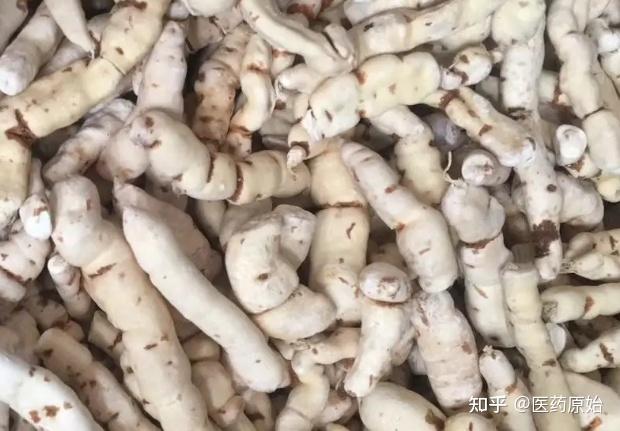

Supplement: S6 File — Numerical data underlying all figures and reported metrics, including complete training logs, evaluation results, per-class performance values, and confusion matrices. (ZIP) [file pone.0344262.s006.zip › Dataset/sample_images/gansui/a8027875821a03fbf13c8ddb4486bd9d.jpg]

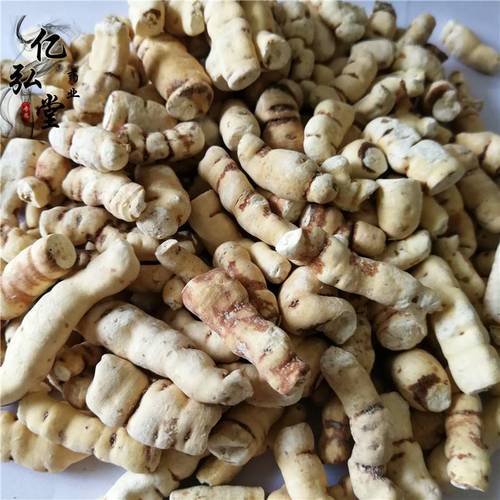

Supplement: S6 File — Numerical data underlying all figures and reported metrics, including complete training logs, evaluation results, per-class performance values, and confusion matrices. (ZIP) [file pone.0344262.s006.zip › Dataset/sample_images/gansui/a96967760ca428a5b21d673a90829d28.jpeg]

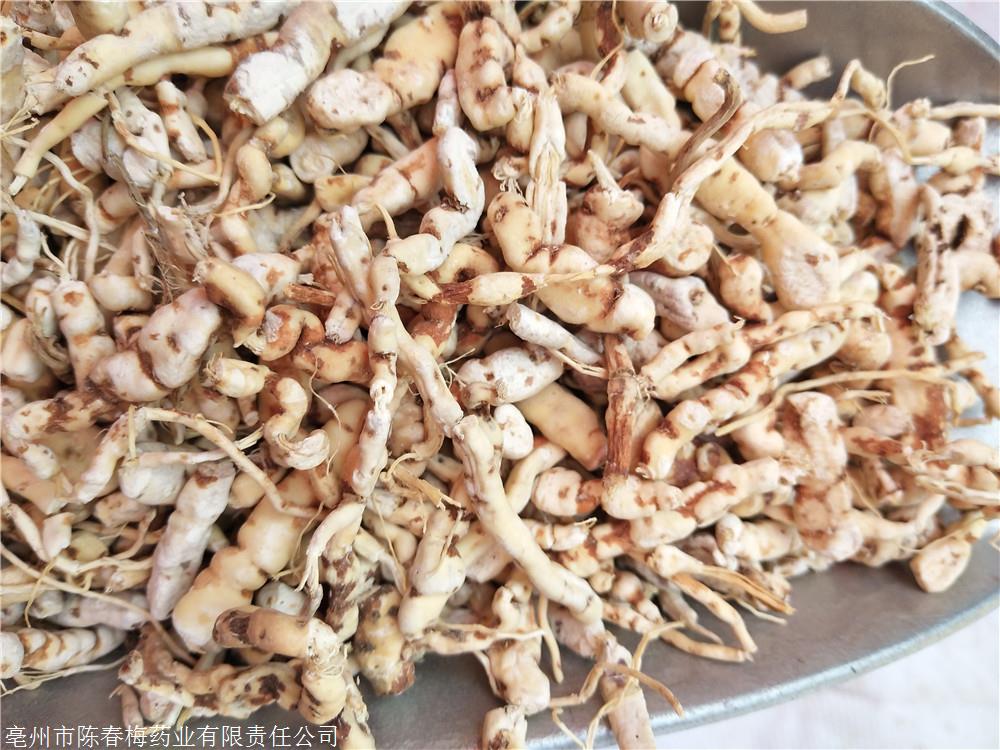

Supplement: S6 File — Numerical data underlying all figures and reported metrics, including complete training logs, evaluation results, per-class performance values, and confusion matrices. (ZIP) [file pone.0344262.s006.zip › Dataset/sample_images/gansui/d6f1cda09c122dc769849cd7af06af53.jpg]

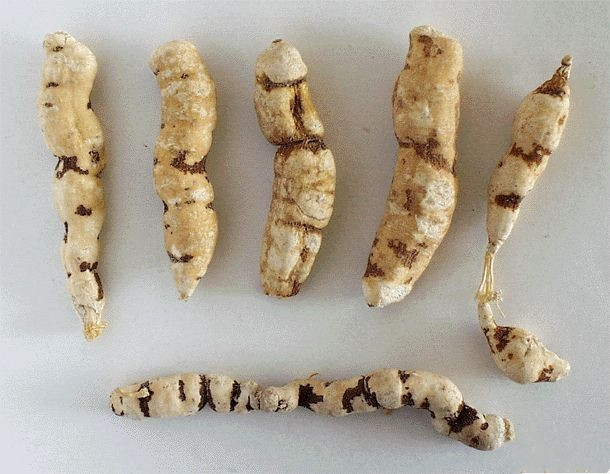

Supplement: S6 File — Numerical data underlying all figures and reported metrics, including complete training logs, evaluation results, per-class performance values, and confusion matrices. (ZIP) [file pone.0344262.s006.zip › Dataset/sample_images/gansui/e1cec5e203ca1a253d9d8c0c4ea5c829.jpg]

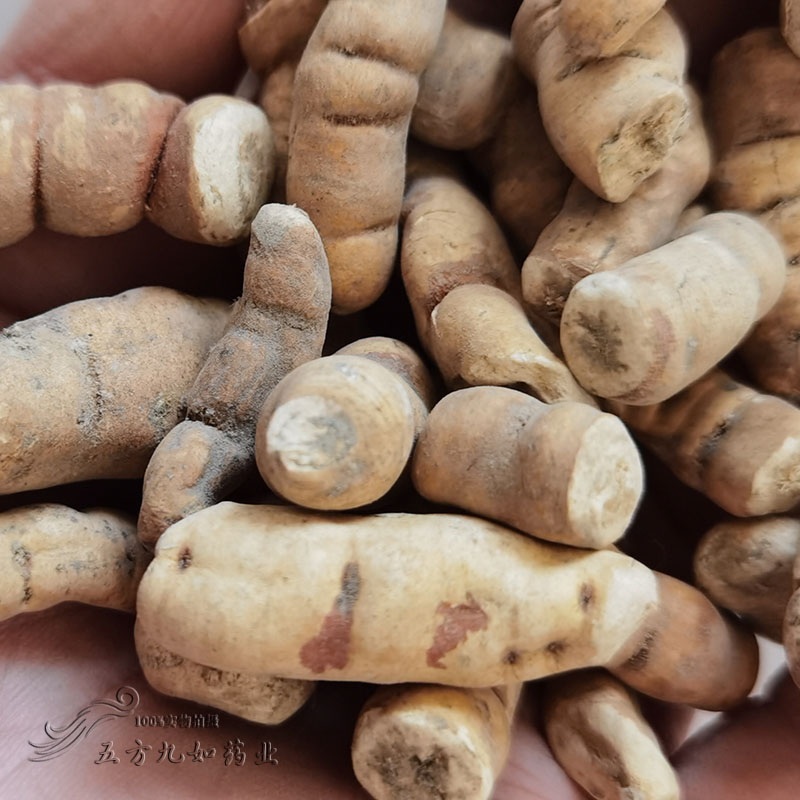

Supplement: S6 File — Numerical data underlying all figures and reported metrics, including complete training logs, evaluation results, per-class performance values, and confusion matrices. (ZIP) [file pone.0344262.s006.zip › Dataset/sample_images/gansui/efdef2f239a4f9208a5b4cfe5ff517ea.jpg]

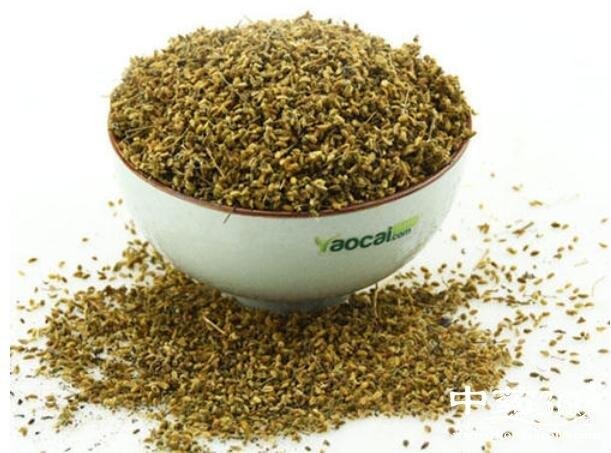

Supplement: S6 File — Numerical data underlying all figures and reported metrics, including complete training logs, evaluation results, per-class performance values, and confusion matrices. (ZIP) [file pone.0344262.s006.zip › Dataset/sample_images/heshi/20f97ff17488bc177d1847327bb9ac0b.jpeg]

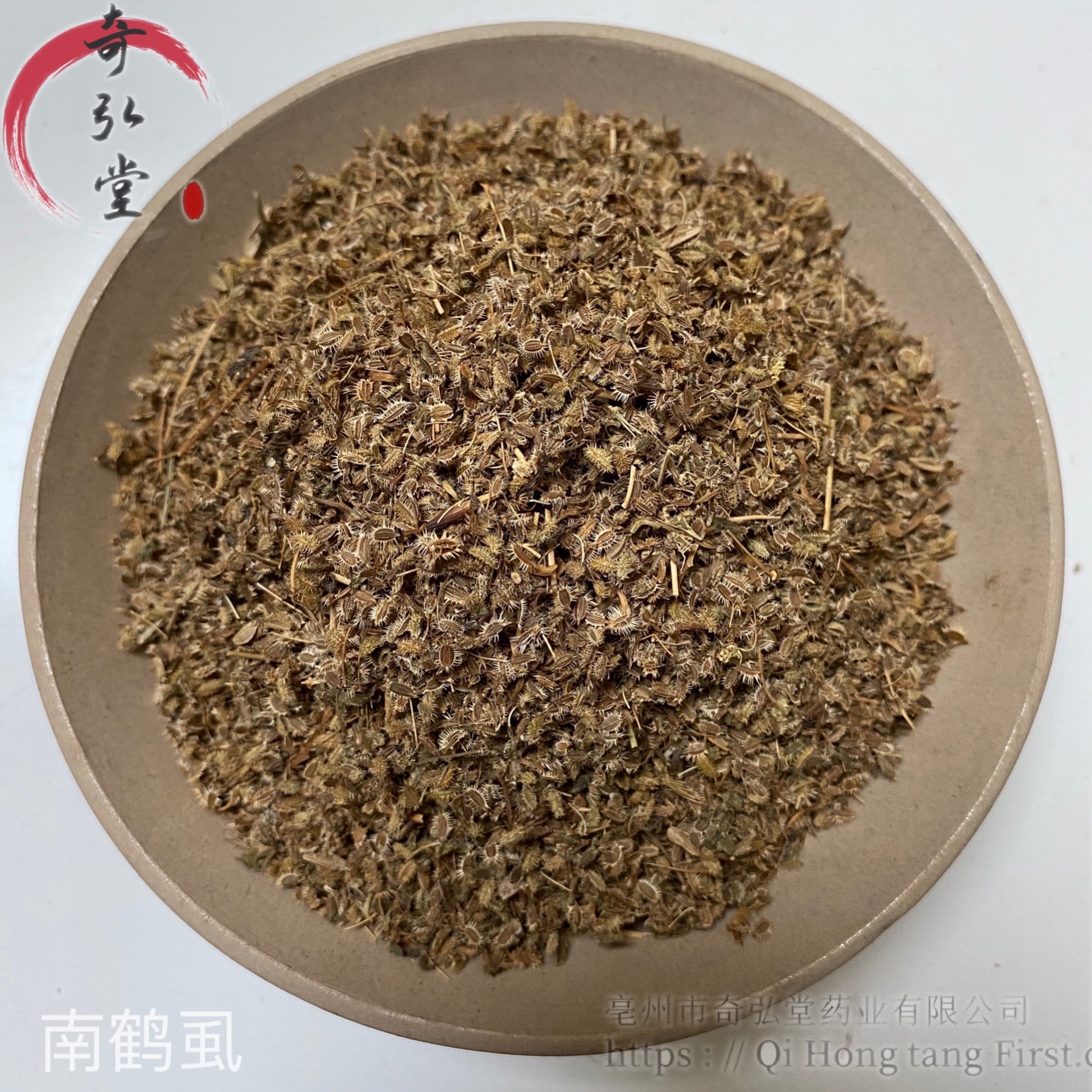

Supplement: S6 File — Numerical data underlying all figures and reported metrics, including complete training logs, evaluation results, per-class performance values, and confusion matrices. (ZIP) [file pone.0344262.s006.zip › Dataset/sample_images/heshi/25ebc26d9ec67168452c6cf783c22254.jpg]

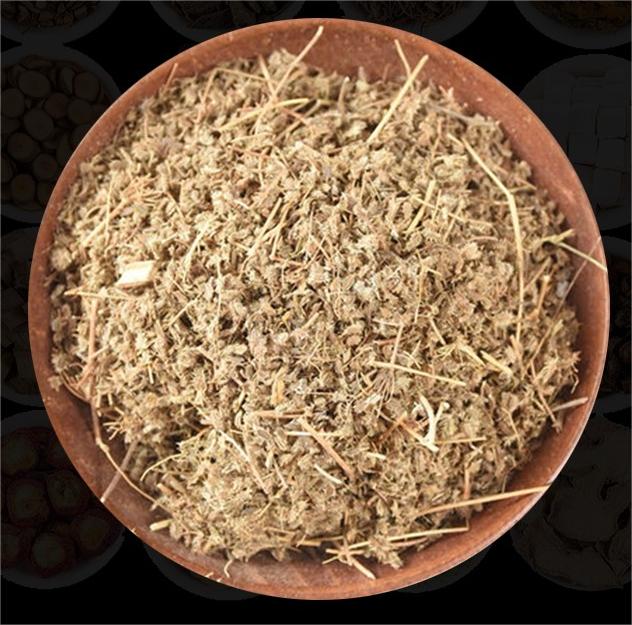

Supplement: S6 File — Numerical data underlying all figures and reported metrics, including complete training logs, evaluation results, per-class performance values, and confusion matrices. (ZIP) [file pone.0344262.s006.zip › Dataset/sample_images/heshi/3ae5286cf73f4138613077d98f0c9789.jpg]

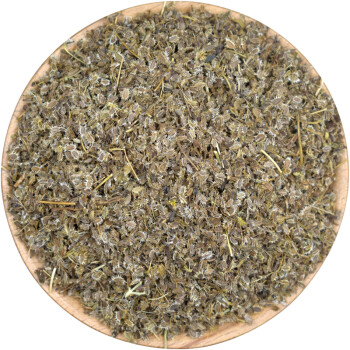

Supplement: S6 File — Numerical data underlying all figures and reported metrics, including complete training logs, evaluation results, per-class performance values, and confusion matrices. (ZIP) [file pone.0344262.s006.zip › Dataset/sample_images/heshi/6b7177eb3b3820833aa17506ce6c6039.jpg]

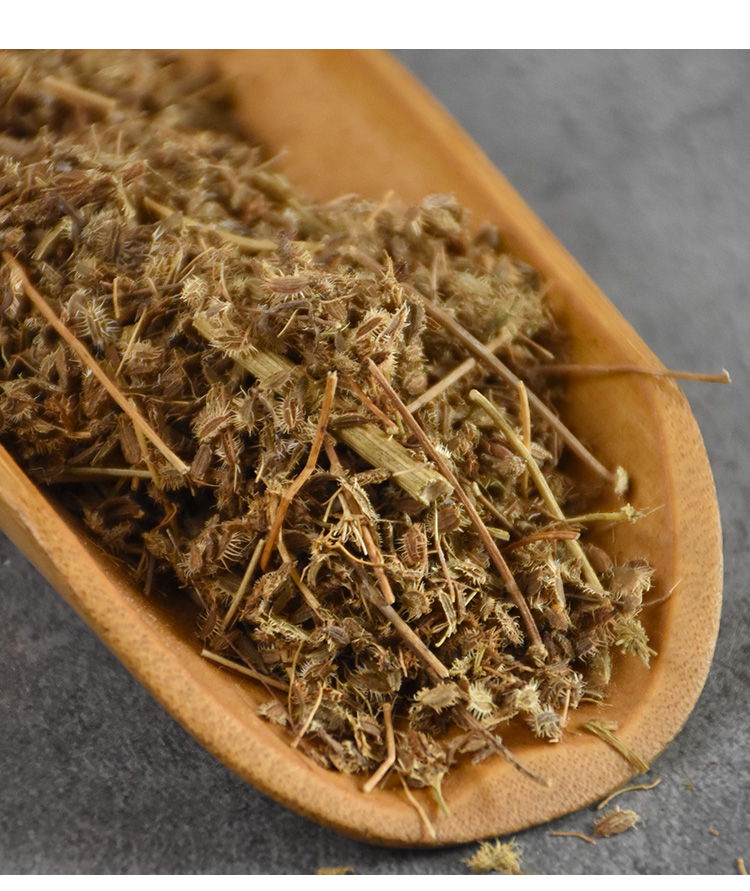

Supplement: S6 File — Numerical data underlying all figures and reported metrics, including complete training logs, evaluation results, per-class performance values, and confusion matrices. (ZIP) [file pone.0344262.s006.zip › Dataset/sample_images/heshi/cf1e2873579fd393bfb9eb58297910e3.jpg]
